# Colorectal Cancer: Genetic Abnormalities, Tumor Progression, Tumor Heterogeneity, Clonal Evolution and Tumor-Initiating Cells

**DOI:** 10.3390/medsci6020031

**Published:** 2018-04-13

**Authors:** Ugo Testa, Elvira Pelosi, Germana Castelli

**Affiliations:** Department of Hematology, Oncology and Molecular Medicine, Istituto Superiore di Sanità, 00161 Rome, Italy; elvira.pelosi@iss.it (E.P.); germana.castelli@iss.it (G.C.)

**Keywords:** colorectal cancer, adenomatous polyp, serrated polyp, cancer stem cells, tumor xenotrasplantation assay, gene sequencing, gene expression profiling

## Abstract

Colon cancer is the third most common cancer worldwide. Most colorectal cancer occurrences are sporadic, not related to genetic predisposition or family history; however, 20–30% of patients with colorectal cancer have a family history of colorectal cancer and 5% of these tumors arise in the setting of a Mendelian inheritance syndrome. In many patients, the development of a colorectal cancer is preceded by a benign neoplastic lesion: either an adenomatous polyp or a serrated polyp. Studies carried out in the last years have characterized the main molecular alterations occurring in colorectal cancers, showing that the tumor of each patient displays from two to eight driver mutations. The ensemble of molecular studies, including gene expression studies, has led to two proposed classifications of colorectal cancers, with the identification of four/five non-overlapping groups. The homeostasis of the rapidly renewing intestinal epithelium is ensured by few stem cells present at the level of the base of intestinal crypts. Various experimental evidence suggests that colorectal cancers may derive from the malignant transformation of intestinal stem cells or of intestinal cells that acquire stem cell properties following malignant transformation. Colon cancer stem cells seem to be involved in tumor chemoresistance, radioresistance and relapse.

## 1. Introduction

Colorectal cancer is one of the most frequent malignancies worldwide, being second in males and third in females for its frequency and ranking fourth and third for cancer-related deaths among males and females, respectively. Colorectal cancer is the second most common cause of cancer death in Europe. It was estimated that more than one million individuals develop worldwide colorectal cancer each year and the disease-related mortality corresponds to about 33% in the developed world. The National Cancer Institute estimated 135,430 new cases of colorectal cancer in USA in 2017, corresponding to 8% of all new cancer cases; the estimated number of deaths in 2017 in the USA was 50,260, corresponding to 8.4% of all cancer deaths. Based on the 2012–2014 data, it was estimated that 4.3% of men and women will be diagnosed with colorectal cancer at some point during their lifetime. From 1992 to 2014 there was a consistent decrease in the incidence of new cases of colorectal cancer. Based on the data observed in the period 2007–2013, it was estimated that about 65% of patients survive five years or more after being diagnosed with colorectal cancer.

From a clinical point of view, colon cancers are usually subdivided as proximal or right-sided when they originate from colon sections proximal to the splenic flexure (cecum, ascending colon and transverse colon), whereas distal or left-sided colon tumors arise distally with respect to this site (descending colon and sigmoid colon). Finally, colon cancers are classified as rectal cancers when they arise within 15 cm of the anal sphincter. Rectal cancers show higher rates of loco-regional relapse and lung metastases, whereas colon cancers have a higher tropism for liver spread and usually have a moderately better prognosis.

Most colon cancers are classified as adenocarcinomas, subdivided according to the grade of the tumor into low-grade and high-grade. Rarer histological subtypes are represented by mucinous adenocarcinoma, adenosquamous carcinoma, signet-cell carcinoma and medullary carcinoma. There is no simple link between the histotype and tumor prognosis. In this context, the available evidence suggests that medullary colon cancer is associated with microsatellite instability (MSI) and seems to be associated with a better prognosis, while signet-ring cell carcinomas have a poor prognosis.

The present review paper offers a comprehensive description of the progress made in the last three decades in the understanding of the molecular basis of colorectal cancers. The recent innovations in molecular biology and cancer genetics have improved the understanding of the pathogenesis of sporadic and hereditary colorectal cancer syndromes. The development of sophisticated molecular techniques for the analysis of the genome has enabled the identification of several genetic alterations involved in the pathogenesis of colorectal cancer. The development of colorectal cancer is dictated by a mixture of genetic and environmental factors.

This progress in the molecular pathogenesis of colorectal cancer has allowed the definition of two pathways of cellular/molecular development of colorectal cancer, starting from two different precursor lesions: adenoma-carcinoma pathway and the serrated pathway, characterized by different genetic lesions. The molecular studies also helped to define the inter-tumor and the intra-tumor clonal/mutation heterogeneity of colorectal cancers and have in part elucidated the mechanisms involved in the acquisition of the metastatic activity of colorectal cancer cells.

The progress in molecular studies has been paralleled by cellular studies that have determined the tissue units of epithelial intestinal development and their cellular organization. These tissue units are hierarchically organized and depend for their survival on two populations of stem cells, located at the base of the crypts. The oncogenic transformation of the intestinal epithelium involves the cancerization of intestinal stem/precursor cells. Cancer stem cells, cells able to initiate and maintain the tumor process, have been identified and characterized in colorectal cancers.

From a genomic standpoint, colorectal cancer is not a single disease, but a heterogeneous group of malignancies arising within the colon. Genomic analysis of metastatic colorectal cancer provides important prognostic and predictive information for the clinician. Particularly, the genomic analysis provides data on the presence of activating mutations in the *KRAS, NRAS* and *BRAF*, thus providing criteria for the selection of patients for the anti-epidermal growth factor receptor (EGFR), Cetuximab or Panitumumab. Mutations in these genes are mutually exclusive and globally occur in about 55–60% of colorectal cancers. Patients with *KRAS*, *NRAS* or *BRAF* mutations do not benefit from anti-EGFR therapies. In addition to providing predictive and prognostic information, multigene sequencing for the molecular profiling of colorectal cancer will provide data to discriminate between microsatellite stability (MSS) and MSI. MSI-high (MSI-H) colorectal cancers result from mutations in mismatch repair (MMR) genes that cause a multifunctioning gene product or from promoter methylation causing the epigenetic silencing of MMR protein expression (MMR-deficient). MSI-H or MMR-deficient colorectal cancers may have alternative therapeutic options based on the administration of some immunological agents.

## 2. Colorectal Carcinogenesis

### 2.1. Normal Intestinal Stem Cells

**Figure 1:**
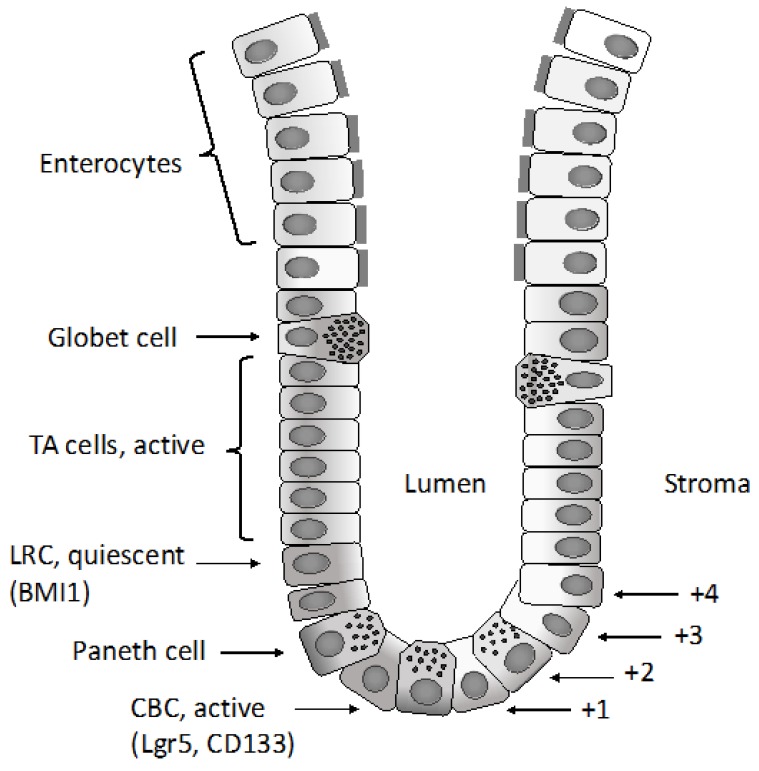
Schematic representation of the large intestine crypt. Each crypt comprises a bottom region, containing crypt base columnar (CBC) cells. These cells are intestinal cycling stem cells, leucine-rich repeat-containing G-protein coupled receptor 5 (LGR5)^+^ and generate all major intestinal lineages, including secretory cells and enterocytes. Crypts also contain Paneth cells, the only mature cells that do not migrate upwards and that remain at the base of crypts, near to LGR5^+^ cells. The +4 region contains a population of quiescent stem cells, identified as Bmi1, LRIG1 or label-retaining cells (LRC). A transit-amplifying (TA) region contains differentiating progenitors/precursors. A top region, corresponding to the tip of villi, contains mature elements (enterocytes, goblet cells, Tuft cells and enteroendocrine cells).

The epithelium of the small intestine is organized into anatomical and functional units of self-renewing crypt-villus (Figure 1). The villi are finger-like protrusions of the gut covered by post-mitotic epithelium and highly maximizing the surface of the absorptive area. Each villus is surrounded by several epithelial invaginations, called crypts, and represents the site of actively proliferating progenitor cells, which sustain the self-renewal of the intestinal epithelium.

Various epithelial cell types compose the intestinal epithelium. The enterocyte is the most frequent cell population present and represents a highly polarized epithelial cell involved in intestinal absorption. Goblet cells secrete mucins and are present both in the villi and crypts. The enteroendocrine cells are involved in the release of a variety of hormones and are located both at the level of the crypts and villi. Tuft cells are also present both in the crypts and villi and are involved in the sensing of the luminal content. Microfold cells have a very peculiar localization at the level of the epithelium recovering the Peyer’s patches, related to their function to act as portals for luminal antigens. Paneth cells are specifically localized at the bottom positions in the crypt in contact with intestinal cells: these cells secrete bactericidal proteins and play an essential role in the maintenance of intestinal stem cells. Finally, intestinal stem cells are present at the bottom of the crypts and are the cellular elements essential for the self-renewal of the intestinal epithelium [1]. In the crypt, the large majority of cells are short-lived and only few specialized cells (Tuft cells, neuroendocrine cells and Paneth cells) are long-lived.

Differentiated cells forming the colon epithelium originate from rare multipotent stem cells resident at the basis of the invaginations of the colon epithelium, commonly known as crypts. The immediate daughter cells of the stem cells proliferate a finite number of times and form a population of transit amplifying cells situated directly above the stem cells. In an intestinal crypt, there are 5–16 intestinal stem cells per crypt and 120–150 transit amplifying cells. Since differentiated epithelial cells of the colon crypts have only a short half-life, a very large number of colon epithelial cells, in the order of 10^14^, must be produced during the mean life of humans. The regulated production of all this large progeny of colonocytes must be orchestrated by primitive cells, known as colon epithelial stem cells, in a tightly regulated pathway that permits the tuning of cell production to physiological needs. The production of colonocytes is ensured through the differentiation of different stem/progenitor cells organized according to a hierarchical pattern. The long-term stem cell function at the level of the colon epithelium is ensured by a population of cells located at the bottom of crypts at the level of a position called +4 [2]. This staminal compartment gives rise to a cell population of differentiating and rapidly dividing progenitors, which in turn generate a differentiated progeny moving up in the crypts [2]. Leucine-rich repeat-containing G-protein coupled receptor 5 (LGR5)^+^ stem cells divide every 24 h and generate transit amplifying progenitors, which migrate upward to differentiate into absorptive enterocytes or goblet and tuft cells. Paneth cells do not migrate upward and reside at the bottom of the crypts, intermingled with LGR5^+^ stem cells. Paneth cells are long-lived and play a very important role in intestinal homeostasis, providing a niche for LGR5^+^ cells and releasing factors essential for the survival and proliferation of these cells, including Wnt ligands and EGF: their loss results in the loss of LGR5^+^ cells, while their co-culture with LGR5^+^ cells stimulates the proliferation of these stem cells. Lineage mapping studies have led to the identification of two types of intestinal stem cells: quiescent and proliferative stem cells. The quiescent stem cells are marked by Lrig1, a pan- erythroblastic leukemia viral oncogene homolog (ERB) inhibitor, and are located at the crypt base and, upon injury, proliferate and divide to repair damaged crypts [3]. In contrast, highly proliferative colonic stem cells are LRG5-positive. The transcriptome profiling of quiescent intestinal stem cells is different from that of proliferative stem cells and is characterized by the expression of genes acting as cell cycle inhibitors or involved in the response to oxidative damage [3]. Several recent studies suggest that LGR5^+^ colon stem cells have a considerable repopulating activity and are able to reconstitute a functional tissue in vivo. Thus, Yui and coworkers have shown that colonic LGR5^+^ stem cells, expanded from a single LGR5^+^ cell, are capable of functional engraftment and of the long-term repopulation reparation of damaged colonic tissue [4]. Similar observations have been made in another study showing that a single mouse LGR5^+^ colon stem cell can be expanded into a three-dimensional organoid that, after transplantation, contributes to the repair of injured epithelia in a mouse model of colitis [5]. Therefore, according to these findings, it was unclear whether there is a single intestinal crypt stem cell located at the bottom of the crypt (position +5) or whether there is a second type of intestinal stem cell located at position +4, just above the bottom crypt cells. A very recent study clarified this issue and proposed a unifying view [6]. In fact, Buczacki and coworkers used an elegant genetic strategy to mark quiescent cells at the level of intestinal crypts: using this approach they marked Paneth cells and progenitor/stem cells LGR5^+^ [6]. In healthy mice, the marked quiescent cells failed to divide; in contrast, when crypts were damaged, these quiescent cells proliferated and gave rise to clones comprising the main epithelial cell types and, therefore, acted as an effective clonogenic reserve of intestinal stem cells [6]. Based on this unifying theory, it is now proposed that cycling LGR5^+^ stem cells are the fuel of crypt self-renewal. These cells, through their differentiation, generate cells with the properties of quiescent +4 LGR5^+^ stem cells; these daughter cells, through differentiation, can generate Paneth cells [7]. It is important to note that Paneth cells are long-lived, while the quiescent +4 cells are short-lived. These quiescent cells for their short half-life could be not considered as true stem cells; however, since they are continuously generated from cycling LGR5^+^ cells, they represent a valuable reservoir of stem cells, able to participate in tissue regeneration when required [7].

Studies carried out in the last years have reconciled the crypt base columnar stem cells (LGR5^+^ cells) model with the +4 model and have shown that cycling LGR5^+^ stem cells ensure the renewal and maintenance of the intestinal epithelium and generate long-lived progenitors located at +4/+4/5; these progenitors can regain stemness and act as facultative stem cells in conditions where their function is required [8]. This view is supported by the observation that only when both stem cell sources are depleted, will severe crypt-loss ensue [9]; in contrast, intestinal homeostasis can be maintained after depletion of LGR5^+^ cells due to the activation of LGR5^−^ reserve stem cells [9].

In addition to +4 stem cells, there is also a population of secretory progenitor cells, including label-retaining cells expressing Paneth and enteroendocrine cell markers and delta-like ligand 1 (Dll1)^+^. Transit amplifying (TA) cells, able to revert to the stem cell state when radiation-induced damage occurs at the crypt [10,11]. A recent elegant study showed that these progenitors, identified according to alkaline phosphatase expression, can differentiate and act as facultative reserve stem cells to replenish the stem cell compartment when LGR5^+^ cells are experimentally ablated [12]. The ensemble of these observations strongly supports the view that several different intestinal progenitor cell populations can function as emergency-reserve stem cells and highlights the existence of a consistent cell plasticity able to protect the stem cell compartment from various types of cell injuries.

The self-renewal and proliferation of LGR5^+^ stem cells both in vitro and in vivo are dependent on direct cell contact between LGR5^+^ cells and Paneth cells: for this reason, LGR5^+^ cells cannot be grown in vitro as single LGR5^+^ cells, in the absence of Paneth cells. Indeed, when cultured as organoids, intestinal stem cells spontaneously differentiate into all epithelial cell types, with stem cells maintained only at the tip of crypts. However, a recent study showed a strategy to bypass these limitations in order to grow in vitro LGR5^+^ stem cells [13]. In fact, Yin and coworkers showed that the addition of two small molecules, Chiron Corporation (CHIR) 99021 and valproic, acts synergistically to maintain the self-renewal of mouse LGR5^+^ stem cells, resulting in the generation of nearly homogenous cell cultures [13]. These culture conditions could be used also for the amplification of multipotent human intestinal cells [13]. A single cell gene expression study provided evidence that LGR5^+^ stem cells are a heterogeneous cell population, composed by a majoritarian cell population of stem cells and a minority population of LGR5^+^ secretory cells [14].

There is also a metabolic cooperation between Paneth cells and LGR5^+^ cells; in fact, LGR5^+^ cells have high mitochondrial activity, required for their stem cell activity; Paneth cells support stem cell function by providing the lactate necessary to sustain the enhanced mitochondrial oxidative phosphorylation in LGR5^+^ cells [15].

As mentioned above, in murine small intestinal crypts, Paneth cells contribute to the cellular niche for LGR5^+^ stem cells with which they are in strict physical contact. These niches are essential for mediating growth and maintenance signals and Paneth cells provide molecules such as WNT3A, EGF and NOTCH ligands, required to maintain intestinal stem cells. However, in the colon there are no typical Paneth cells, but there are regenerating islet-derived family member 4 (REG4)-positive deep crypt secretory cells, present at the bottom of crypts, near LGR5^+^ cells [16]. REG4^+^-sorted cells sustain organoid formation by LGR5^+^ cells, while their ablation disrupts colon homeostasis [16]. Deep secretory cells can be produced by LGR5^+^ cells by WNT activation and NOTCH inhibition [16]. According to these observations, it was concluded that deep crypt secretory cells serve as Paneth equivalents in the colon crypt niche.

**Figure 2:**
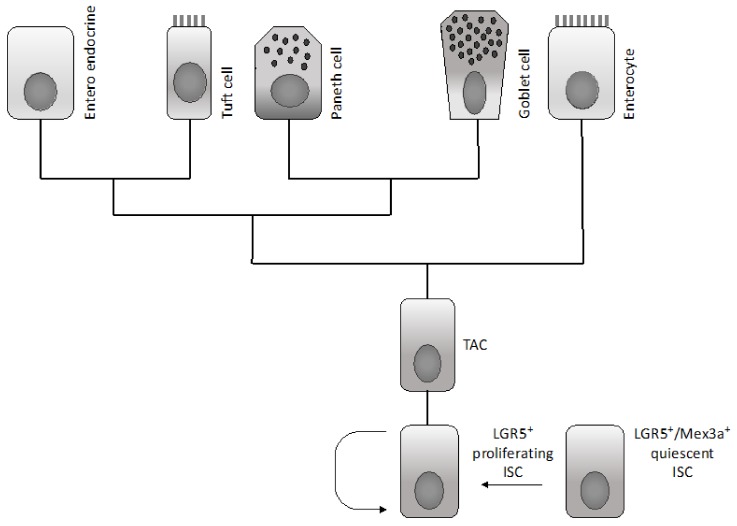
The LGR5^+^ stem cell compartment is heterogeneous, comprising a majority of LGR5^+^ cycling intestinal stem cells and a minority of LGR5^+^/Mex3a^+^ quiescent, chemotherapy- and radiation-resistant intestinal stem cells. TAC: transit amplifying cell; ISC: intestinal stem cell.

A recent study provided evidence that the LGR5^+^ stem cell population is heterogeneous. The expression of the RNA-binding protein Mex3a labels a slowly cycling subpopulation of LGR5^+^ intestinal stem cells that contribute to all intestinal lineages with different kinetics, compared to the predominant cycling population of LGR5^+^ intestinal stem cells (Figure 2) [17]. Single-cell transcriptomic studies showed that LGR5^+^ stem cells adopt two discrete states, one of which is characterized by a Mex3a expression program and low levels of proliferation genes [17]. During homeostasis, Mex3a^+^ cells shift into the rapidly cycling LGR5^+^ cells; chemotherapy and radiation target the cycling LGR5^+^ cell population, but largely spare the Mex3a^+^/LGR5^+^ cell population [17].

The development of an efficient technology for the generation of organoids in vitro is of fundamental importance for a better understanding of the physiology of normal and neoplastic intestinal stem cells. Organoids formed by self-organizing intestinal stem cells resemble their normal physiological counterpart both in their cellular content (generation of various cell lineages), multicellular architecture (tissutal organization) and functional properties [18]. Particularly, an efficient development of organoids requires appropriate extracellular matrix components. Synthetic hydrogel networks are used to form the scaffold for organoid generation; some constituents and properties of this extracellular matrix are required for optimal organoid generation: (i) fibronectin is an essential matrix for intestinal stem cell adhesion and for their survival and proliferation; (ii) high matrix stiffness is required for optimal stem cell expansion through a yes-associated protein 1(YAP1)-dependent mechanism; (iii) soft matrix and laminin adhesion are required for intestinal stem cell differentiation and organoid formation [19].

The ensemble of these studies has suggested that in the intestinal epithelium the epithelial stemness is not a fixed property ascribable to fixed, defined cells, but rather an induced state regulated by signals generated within and around the stem cell niche. This conclusion is directly supported by in vivo imaging studies of mouse crypts, showing that in each crypt there are about 16 LGR^+^ cells, some of which are located at the center-bottom of the crypt, while the others are higher up, being located at the border of the crypt base [20]. The probability of center and border stem cells acting as true stem cells is different: in fact, the central LGR^+^ cells are more likely to act as stem cells than the border LGR^+^ cells [20]. This condition not fixed in time because there is constant flux and constant transfer of cells between these two regions [20].

The WNT pathway plays an essential role in the maintenance, proliferation and differentiation of intestinal stem cells. The *Wnt/β-catenin* target genes show their highest expression at the crypt bottom. The LGR5 and LGR4 proteins expressed on intestinal stem cells function as receptors for the secreted R-spondin proteins, acting as local enhancers of Wnt/β-catenin signaling. In parallel, Wnt/β-catenin signaling acts as a differentiation signal for Paneth cells and controls their localization at the level of crypts. It is important to note that the homeostasis of intestinal cell cells is controlled through not only positive regulators such as the Wnt signaling pathway, but also through negative regulators. In this context, a recent study by Barry and coworkers provided evidence that the YAP1 exerted a growth-suppressive function, restricting Wnt signaling during intestinal regeneration [21]. Cells expressing the YAP protein are located at the base of the crypts. These observations indicate that YAP1 is an important regulator of intestinal stem cells. In line with this function, overexpression of this protein in transgenic mice reduced Wnt-induced target genes and resulted in the rapid loss of intestinal crypts. In contrast, loss of YAP resulted in a hypersensitivity to Wnt, with a consequent expansion of intestinal stem cells and niche cells [22]. LGR5^+^ cells possess specific molecular mechanisms that protect them from the danger of excessive Wnt signaling. In fact, a recent study showed that LGR5^+^ intestinal stem cells express the tumor suppressor RNF43, a stem cell E3-ligase-inducing endocytosis of Wnt receptors: thus, this ligase reduces Wnt signals by selectively ubiquitinating frizzled receptors, thereby targeting these Wnt receptors for degradation [21]. Wnt is required to induce the expression in LGR5^+^ cells of the helix–loop–helix (bHLH) transcription factor Ascl2, able to bind to the LGR5 promoter [23]. Ascl2 expression in LGR5^+^ cells may create a self-perpetuating state of stemness within the intestinal stem cell population, thus reinforcing the stem cell identity of these cells [23].

It is important to point out that LGR5, as well as LGR4 and LGR6, acts as a high-affinity R-spondin receptor and a LGR receptor triggers β-catenin signaling through a process requiring LGR internalization [24]. The LGR5/R–spondin complex acts by neutralizing RNF43 and ZNRF3, two transmembrane E3 ligases that remove WNT receptors from the stem cell surface [24]. It is also important to note that RNF43 and ZNRF3 are themselves encoded by WNT target genes and constitute a negative WNT feedback loop [24].

In addition to the three signaling pathways—WNT, bone morphogenetic protein (BMP) and NOTCH—that play a major role in the homeostasis of intestinal stem cells in the intestinal stem cell niche, the TGF-β pathway also seems to play a major role. In fact, TGF-β signaling modulates the generation of secretory cell progenitors from intestinal stem cells, and, through this mechanism, controls intestinal stem cell differentiation [25].

R-spondin ligands engage different LGR4–6 receptors and markedly potentiate WNT/β-catenin signaling and induce intestinal organoid growth in vitro. However, the R-spondin and WNT-mediated signaling are not interchangeable: WNT proteins are unable to induce the self-renewal of LGR5^+^ intestinal stem cells, but instead confer a basal competency by maintaining R-spondin receptor expression, which enables R-spondin ligands to activate their receptors (LGR 4–6, RNF43 and ZNRF3) to drive stem cell expansion [26]. Therefore, there is a functional cooperation between WNT and R-spondin pathways in mediating self-renewal in intestinal stem cells.

Other studies have identified Bmi1^+^ cells, located just above the base of the crypt (+4 position), as another population of intestinal cells capable of acting as an intestinal stem cell able to proliferate, to expand, self-renew and to give rise to all the differentiated cell lineages [27]. Subsequent studies have clarified the differences between Bmi1^+^ and LGR5^+^ intestinal stem cells: LGR5^+^ cells are sensitive to WNT activators, contribute to homeostatic regeneration and are suppressed by irradiation; Bmi1^+^ cells are quiescent, are apparently insensitive to WNT signaling, contribute weakly to homeostatic regeneration and are resistant to radiation-induced injury [28]. However, after irradiation, Bmi1^+^ cells proliferate rapidly to clonally repopulate contiguous crypts and villi [28]. A recent study showed that Bmi1^+^ cells are enriched in enteroendocrine markers, including Prox1 [29]. Lineage-tracing experiments showed that a part of Prox1^+^ cells exhibited sustained clonogenic growth in vitro and are capable of repopulating activity in vivo [29]. According to these observations, it was concluded that enteroendocrine cells contribute to a reservoir of injury-inducible intestinal stem cells [29]. Another recent study also showed that Bmi1^+^ cells are preterminal enteroendocrine intestinal cells [30]. Importantly, upon the loss of native LGR5^+^ cells, Bmi1^+^ cells revert to LGR5^+^ cells [30].

According to all these observations, a two-stem cell model is emerging involving the existence of an actively proliferating, but injury-sensitive stem cell and a rare, injury-resistant pool of quiescent stem cells [31]. Proliferating LGR5^+^ cells are able to promote a rapid regeneration of epithelial colon cells during basal homeostasis and in response to injury. The reserve, quiescent Bmi1^+^ stem cells display several properties, including resistance to DNA damage; quiescent (G0, quiescent cells); resistance to the stimulation of WNT pathways; capacity to generate all intestinal epithelial cell lineages; and a requirement for the maintenance of the intestinal epithelium in the basal state and for epithelium regeneration after injury [31]. This organization of the intestinal epithelium is to some extent reminiscent of the stem/progenitor organization of the hematopoietic system.

An important mechanism operating at the level of intestinal stem cells prevents the accumulation of mutated cells. This mechanism is the neutral drift of intestinal stem cells. In fact, it was believed for long time that intestinal stem cells divide asymmetrically (i.e., daughter cells adopt divergent cell fates). However, clonal tracing experiments of individual LGR5^+^ stem cells showed that these cells divide symmetrically (i.e., they generate two daughter cells adopting identical cell fates) [32]. This important finding supports a simple principle of the random replacement of intestinal stem cells, where a single intestinal stem cell in a crypt can be replaced by any of the other intestinal stem cells present in the crypt: this process is called neutral drift. These findings also support a model in which resident stem cells double their number each day and stochastically adopt either stem or transit amplifying fates [32,33]. Taking into account this model of stem cell dynamics, some investigators have evaluated the effect of relevant mutations on stem cell dynamics during the initiation of colon cancerogenesis. These studies have shown a limited competitive advantage of common mutations in colorectal cancers and many mutations that occur are lost from the population because of the stochastic replacement of neighboring wild-type lineages [34].

Colon cancer development is mainly related to an intrinsic genomic instability of cells present at the level of human colon crypts. The existence of this genomic instability is directly supported through the analysis of individual human colon crypts derived from healthy individuals of different ages; chromosomal changes—consisting of deletions, duplications and gene conversion events—are detected in individual crypts with increasing age in the absence of any pathological process at the level of the colon epithelium [35]. The accumulation of mutations at the level of intestinal stem cells occurring with aging is a key mechanism promoting colon cancer development. This phenomenon was observed in various animal species, including insects (*Drosophila*). In fact, two mechanisms of genome instability occurring at the level of adult insect intestinal stem cells promote aging-associated changes predisposing to cancer development: loss of heterozygosity deriving from mitotic homologous recombination, resulting in genetic mosaicism; and the somatic deletion of DNA and large structural rearrangements, resembling those observed in cancers and resulting in gene inactivation [36]. Human intestinal stem cell and crypt dynamics were recently characterized, showing findings compatible with the neutral drift model. According to these data, it was concluded that human intestinal cells conform to one-dimensional drift dynamics with a functional number of intestinal stem cells per crypt corresponding to 5–6 in both normal individuals and patients with familial polyposis (germline adenomatous polyposis coli (*APC*)^−/+^). In adenomatous crypts, both the number and the turnover of intestinal stem cells increase [37]. Finally, it was estimated that a normal colon crypt divides once every 30–40 years, and the division rate is increased in adenomas.

In conclusion, the studies carried out on murine intestinal stem cells have shown that LGR5^+^ stem cells behave as intestinal stem cells and in part also Bmi1^+^ stem cells. Disruption of LGR5^+^ stem cells triggers epithelial renewal from Bmi1^+^ cells, from secretory or abortive progenitors, and from Paneth cell precursors, thus revealing a high degree of plasticity within the various cell compartments of intestinal crypts.

The identification of human intestinal stem cells using the LGR5 surface marker is difficult due to the low expression of this receptor on the membrane of human intestinal cells and due to the lack of high-affinity anti-LGR5 antibodies. The characterization of human colonic organoids has led to the identification of a cell population expression tyrosine pseudokinase PTK7 and characterized by high self-renewal capacities and by various biological properties comparable to those observed for murine LGR5^+^ cells [38].

### 2.2. Oncogenic Transformation of Intestinal Stem Cells

**Figure 3:**
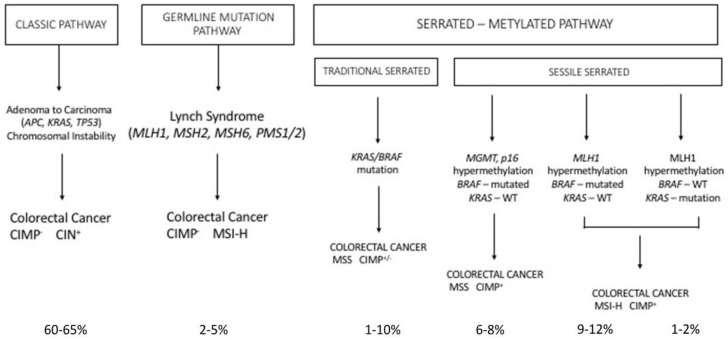
Outline of the three main pathways of colon carcinogenesis. A classic, conventional pathway is initiated by *APC* mutations and progresses through the sequential accumulation of genetic mutations and chromosomal instability (CIN), causing microsatellite stable (MSS) tumors. The germline mutation pathway is related to germline mutation of mis-match repair (MMR) genes, seen in Lynch syndrome (hereditary non-polyposis coli) and leads to microsatellite instability (MSI-H). The sessile-serrated-methylation pathway is heterogeneous: a traditional serrated pathway, related to *KRAS/BRAF* mutations, leading to MSS tumors, with a variable CpG island methylator phenotype (CIMP); a traditional serrated pathway, comprising three subgroups: one leading to MSS and CIMP^+^ tumors, one associated with *BRAF* mutated and *KRAS-WT* and hypermethylation of *MGMT* and p16 gene promoters; the two others leading to MSI-H and CIMP^+^ tumors, one associated with *BRAF*-mutations and *KRAS-WT* and hypermethylation of the *MLH1* gene promoter and the other one associated with *BRAF-WT* and *KRAS*-mutated and hypermethylation of the *MLH1* gene promoter.

Cancer development is commonly regarded as a multistep process involving an initial mutagenic event called tumor initiation: in this initial event, a genomic mutation leads to a malignant phenotype, associated with only a limited growth advantage over the normal counterpart. This initial event is followed by additional mutagenic events and/or epigenetic events, collectively known as tumor promotion, involving the growth of a mutated cell clone and proliferation of tumor cells. This process culminates with clinically detectable tumor development and is known as tumor progression. Colon cancer represents a unique model to explore these different stages of tumor development. Fearon and Vogelstein initially proposed a model, called the adenoma → carcinoma sequence model, in which certain mutations were directly related to different stages of tumor development [39]. In line with this model, tumor initiation was triggered by mutations occurring at the level of the *APC* gene, which is responsible for adenoma formation and the development of a so-called “dysplastic crypts”. After this stage, the occurrence of additional mutations at the level of *K-RAS*, *p53* and *SMAD4*, favor tumor promotion and progression, characterized by the increased growth rate of the adenoma, the expansion of individual particularly malignant clones, with consequent tumor invasion and metastasis (Figure 3).

As mentioned above, the current evidence indicates that mutations at the level of the *APC* tumor suppressor gene initiate the process of colon tumor formation. Germ-line mutations in the *APC* gene cause a hereditary cancer syndrome known as familial adenomatous polyposis (FAP). FAP patients carry heterozygous *APC* mutations. The second allele is frequently lost in cells growing into colon adenomas and polyps. *APC* function in the normal colon is related to a negative regulation of Wnt signalling through the targeting of β-catenin for proteosomal degradation. In cells harboring a mutated *APC* gene, due to the absent inhibitory effect exerted by Wnt signalling, β-catenin accumulates and, after its translocation in the nucleus, acts as a co-activator of T-cell factor (TCF)-lymphocyte enhancer factor (LEF). The β-catenin/TCF-LEF complex acts in turn as a transcriptional activator of key cell-cycle regulatory genes, *cyclin D1* and *c-Myc*. Therefore, according to this model of APC function, its loss induces an immediate activation of Wnt signalling and a subsequent dysregulation and nuclear accumulation of β-catenin. Following nuclear translocation, β-catenin displaces transcriptional corepressor, allowing direct binding to the transcription factor, TCF-LEF and the consequent transcription of Wnt-target genes including *LGR5*, *c-Myc*, *Axin2* and *cyclin D1* [40]. Recent studies carried out on human colon cells have shown that *APC* loss induced intestinal differentiation defects, whereas proliferation defects and the nuclear accumulation of β-catenin require the additional activation of *KRAS* [41]. The effects of *APC* mutation-induced intestinal differentiation defects depend on the transcriptional co-repressor C-terminal binding protein-1 (*CtBP1*) [41]. Therefore, following *APC* loss, *CtBP1* contributes to adenoma initiation as a first step, whereas *KRAS* activation and β-catenin nuclear localization promote adenoma progression to carcinomas as a second step [41].

A murine model of the conditional suppression of APC provided clear evidence that *APC* disruption is required for colon tumor development and maintenance [42]. In this model, *APC* suppression determines the formation of adenomas both at the level of the colon and small intestine that—in the presence of additional mutations at the level of *TP53* and *KRAS* genes—induces the progression of these tumors to colon cancers. A particularly intriguing finding was that *APC* restoration in these developed tumors rapidly induces cell differentiation and tumor regression, without tumor relapse [42]. An additional surprising finding was that tumor regression was characterized at the histological level by the re-establishment of a normal crypt-villus tissutal organization, and of a normal self-renewal and differentiation capacity of stem cells [42]. The results obtained in this APC-driven model indicate a fundamental role of *APC* loss in colon cancerogenesis and suggest that colon cancer cells can revert to normal intestinal cells given that APC expression is restored in tumor cells [42].

Patients with familial adenomatous polyposis harbor the *APC* gene mutation in cells throughout their bodies, but they predominantly develop colon cancer; this observation suggests that the *APC* gene mutation requires a specific cellular context, or an organ-specific microenvironment to induce the development of tumors. These observations were confirmed in a reprogrammed model of colorectal cancer, showing that *APC* mutations have distinct, cell-autonomous effects on gene expression in different cell types [43].

The large majority of colon cancer patients carry inactivating APC mutations. It is important to note that in rare cases of colon cancers, the wild-type of the *APC*, *Axin1* or *Axin2* gene is mutated. Patients with *Axin2* germ-line mutations display a predisposition to colon cancer development. Both Axin1 and Axin2 act as negative regulators of β-catenin-dependent WNT signaling.

The analysis of mutations that are benign, invasive and metastatic tumor cells have in common allowed the determination of the times separating these different tumor stages. It was estimated that about 17 years are required for a benign adenoma to develop into a carcinoma, but only two years are required for it to acquire the capacity to metastasize to the cells forming a colon cancer [44]. Furthermore, few new molecular events are required for the acquisition of metastasizing properties by colon cancer cells. The early gene mutations responsible for initial adenoma mutation are represented by *APC* mutations, and for adenoma enlargement by *KRAS* and *BRAF* mutations. Additional mutations for cancer transitions are represented by *TGF-β*, *PI3KCA*, *TP53* mutations [44]. It is important to also note that it was estimated that the rate of acquisition of point mutations in colon cancer cells is similar to the rate observed in the normal tissue [44].

Among the various mutated genes that promote colon cancer progression, a relevant role is certainly played by *TP53*, both for its frequent mutations in colorectal cancer and for its relevant biologic function. Using mice with an intestinal epithelial cell-specific *p53* deletion, evidence was provided that *p53* loss alone was insufficient to cause colon carcinogenesis initiation, but markedly increased carcinogen-induced tumor incidence and determined the development of invasive cancer, with metastatic spreading [45]. In addition to this initial contribution of *p53* loss in the early stages of colon cancer development, at the later stages of colon cancer development, *p53* loss causes an enhancement of tumor progression related to the induction of an inflammatory pro-tumorigenic microenvironment and the induction of the epithelial–mesenchymal transition [45]. This late effect of *p53* indicates that this master tumor suppressor gene exerts its function on colon cancer through a function independent of its well-established role in cell cycle regulation, apoptosis and senescence [45].

Several studies suggest a role of *PIK3CA* mutations in colon carcinogenesis. A recent study reported a FCPIK3CA murine cancer model, based on the expression of a constitutive phosphatidyl-inositol-3 kinase (PI3K) in the intestinal epithelium: this constitutive PI3K activity resulted in tissue hyperplasia and the formation of invasive mucinous adenocarcinomas [46]. In a second study, the same authors generated mice in which the expression of a constitutive PI3K and the loss of *APC* were induced simultaneously in colon cells: these mice developed many aggressive colon adenocarcinomas, thus indicating the capacity of *PI3KCA* mutations to synergize with *APC* loss [47].

The advances in the long-term growth of intestinal LGR5^+^ cells have allowed the unique opportunity to develop an in vitro system of genetic reconstitution of tumorigenesis in primary intestinal cells [48]. The lentiviral transduction of intestinal organoids was performed to inactivate the *APC* gene: the transduced cells showed a constitutively activated WNT signaling pathway and proved to be tumorigenic when inoculated into severe combine immuno deficiency (SCID) mice; these cells also acquired the ability to form tumorspheres in vitro and to give rise to secondary tumors on re-transplantation [48]. Inactivation of *p53* or *PTEN* or *KRAS* activation in these cells promoted tumor development only in the context of *APC* suppression [48].

As mentioned repeatedly above, *APC* loss of function is a key event in colon carcinogenesis, representing the first event in tumor initiation. The idea that at the adenoma precancerous stage, only initiation mutations occurred, was experimentally demonstrated. In this context, Nicholaev and coworkers recently reported the exome sequencing of 24 human colon polyps. The exome analysis showed that driver initiation mutations at the level of either *APC*, *CTNNB1* or *BRAF* genes were mutually exclusive. According to the presence of these mutations, the adenomas were subdivided into three groups: group 1 with *APC* mutations, comprising 20 adenomas, of which one was a hyperplastic polyp, 16 were adenomas and three were adenocarcinomas (all the mutations occurring at the level of the *APC* gene introduced premature stop codons); group 2 with *CTNNB1* mutations occurred only in one adenoma with tubulovillous histology; group 3 with *BRAF* mutations occurred in three adenomas, with serrated histology [44]. In the adenomas of group 2 and 3, no additional mutations at the level of other cancer driver genes were identified; in contrast, in the adenomas with APC mutations additional cancer driver genes (such as *KRAS*, *NRAS*, *TP53*, *GNAS*, *AKT1*, *ARID1A* and *SOX9*) were observed, whose number correlated with the degree of dysplasia and invasiveness. Finally, the analysis of the number of single nucleotide mutations allowed the calculation of the mutation rate in normal colon tissue and in the adenoma tissue and the definition of the existence of a mutator phenotype in the adenomatous tissue [44].

As will be discussed later in the section on tumor heterogeneity, an alternative model of colon cancer evolution, the “Big Bang” model was more recently proposed: this model, based on the concept of punctuated evolution, implies that the majority of genomic alterations accumulate during the early stages of carcinogenesis, before the development of a big tumoral mass [49]. According to this model, small colorectal polyps display different fates, with some growing and some regressing in size and others remaining stable in their size: the polyps growing are in time endowed with peculiar genetic/functional properties because they are “born to be bad” and possess multiple genetic abnormalities [50]. As will be discussed in the section on tumor heterogeneity, the development of gene sequencing techniques allowed the evaluation of the mutational landscape of individual colon polyps, showing their consistent heterogeneity, and that even small polyps have multiple pathogenic mutations in crucial driver genes (*APC*, *KRAS*/*NRAS*, *BRAF*, *FBXW7* and *TP53*).

The type of mutation occurring at the level of the APC genes has a considerable influence on the cellular phenotype induced by the mutation. Somatic mutations occur at the level of the *APC* in the large majority of colorectal cancers, while germ-line mutations of the *APC* gene occur in the FAP, characterized by the formation of a very large number of polyps in the colorectum, with a high risk of cancer transformation of these adenomas (Table 1). At the histological level, most adenomatous lesions start as initial dysplastic crypts, described as aberrant crypt foci, considered as the precursor lesions of polyps. The birth incidence of germline *APC* mutations is between one in 9000 to 18,000. Individuals with classic FAP start developing polyps in late childhood to teen years. FAP accounts for about 1% of all colorectal cancers. Most of the germline mutations of *APC* gene observed in FAP are nonsense or frameshift mutations leading to the synthesis of a truncated protein. In FAP, a positive correlation was observed between the type of mutation and the penetrance of the mutated genes, estimated according to the number of polyps present in the colorectum: thus, mutations in the codons 1250–1464 are associated with the highest number of polyps, while mutations in the 5′ and 3′ regions are associated with a mild phenotype (with <100 adenomatous polyps); these two phenotypes are known as classic FAP and attenuated FAP (AFAP) [51]. In contrast, the mutation of the *APC* gene in sporadic colorectal cancers occurs at the level of an *APC* gene region—codons 1281–1256, forming the so-called mutation cluster region (MCR)—involved in β-catenin downregulation [52]. Without preventive surgical colectomy, the heavy burden of colonic polyps associated with classic FAP confers a lifetime high risk for colorectal cancer >90%. In contrast to classic FAP, in which polyps develop in adolescence, individuals with AFAP tend to develop polyps later in life. In addition to *APC* mutations, colorectal cancers also exhibit aberrant *APC* expression due to loss of heterozygosity (LOH); LOH may also be acquired in colorectal adenomas as a consequence of somatic mutation. Recently, Christie and coworkers analyzed the entire spectrum of mutated events involving the *APC* gene in a large population (624 patients) of colorectal cancer patients [53]. The results of this interesting study showed that: (i) protein-truncating mutations occurred in 69.4% of these patients; (ii) LOH at *APC* was detected in 32.1% of colorectal cancers, mostly caused by chromosomal deletion; and (iii) 74.8% of cancers displayed either a LOH or at least one *APC* truncating mutation [53]. Most truncating APC mutations occurred within a mutation cluster region, leaving intact 1–3 20 amino acid repeats (AARs). The majority of colon cancers have a missense coding region (MCR) mutation plus one LOH or another mutation 5′ to MCR. Colorectal cancers occurring in proximal and distal intestinal regions have different preferred APC genotypes, with a total of 2–3 and 0–2 intact 20 AARs, respectively [53]. The main mechanism through which *APC* mutations contribute to colon neoplasia is mainly related to an activation of the WNT signaling pathway and, through this mechanism, the induction of cell proliferation. The other important consequence of *APC* mutations is related to the induction of chromosomal instability, determining abnormalities in chromosome segregation with consequent aneuploidy and increase of the rate of LOH [54].

The most prevalent genetic changes in colorectal cancers are biallelic APC mutations; less frequently, the WNT pathway may be activated in colorectal cancers by another genetic mechanism consisting of the gain-of-function mutations of the gene encoding β-catenin (*CTNNB1*). *APC* mutations remain constantly represented in the progression from adenomas to colorectal cancers, while *CTNNB1* mutations become less frequent in the tumor spectrum from small adenomas to colorectal cancers. The comparison of the gene expression profile in murine tumors associated with *APC* loss, compared to tumors with *CTNNB1* mutations showed a large spectrum of gene expression overlapping in the two types of tumors, but also a unique set of changes in gene expression due to the loss of chromatin-associated APC and absent in *CTNNB1*-mutant tumors, where such binding is retained [55]. Adenomas from APC^Min/+^ mice are characterized by the increased expression of Serpine 2, in comparison with adenomas with *CTNNB1* mutations [55].

Therefore, the adenoma → carcinoma sequence model indicates that colon cancer tumor progression is dictated by growing genomic instability. Subsequent studies have shown that mutations observed in colon cancer are associated with two types of genomic instability and harbor mutations of different sets of genes: chromosomal instability and microsatellite instability. Chromosomal instability includes the presence of different numerical or structural chromosome changes and is observed in about 70% of colon cancers and has been related to mutations of set of genes following the adenoma → carcinoma sequence model [56]. In contrast, microsatellite instability is observed in about 15% of cases and is characterized by mutations or variations in the length of microsatellite sequences, occurring as a result of defective DNA mismatch repair genes. Besides defects in DNA mismatch repair genes, microsatellite instability has been related to mutations of a peculiar set of genes involving *BAX*, insulin-like growth factor 2 receptor (*IGF2R*) and transforming growth factor receptor 2 (*TGFβR2*) [56]. Colon cancers associated with microsatellite instability have an improved prognosis, compared to colon cancers associated with chromosomal instability.

The adenoma → carcinoma sequence model can be revisited, taking into account the existence of cancer stem cells. These cells are regarded as the cells that initiate and maintain the tumor bulk. According to this view, it was hypothesized that the first mutational hit occurs at the level of a colonic stem cell that, being long-lived, has the opportunity in time to accumulate additional oncogenic mutations and epigenetic changes. Once transformed, cancer stem cells are able to undergo either symmetric or asymmetric cell division, thus generating both other cancer stem cells and progenitors, which, in turn, generate a cancer cell progeny [57].

This cancer stem cell origin of colon cancer recently received some direct experimental support in studies of tumorigenesis. Thus, it was demonstrated that the transformation of stem cells through the loss of *APC* is an extremely efficient route towards initiating intestinal adenomas [58]. Furthermore, observations of LGR5 expression suggest that a stem cell/progenitor cell hierarchy is maintained in early stem-cell-derived adenomas [58]. The ensemble of these observations supports the view that a colon cancer stem/progenitor cell is the cell of origin of colon cancer. Lineage retracing experiments have directly supported the suggestion that LGR5^+^ cells may represent the cells of origin of mouse intestinal adenomas that fuel these tumors [58]. These LGR5^+^ cells represent 5–10% of the cells in the mouse adenomas and generate other LGR5^+^ cells, as well as other cell types composing the adenoma [58].

As mentioned above, the initial events in colon cancer tumorigenesis, corresponding to the stage just after tumor initiation, should lead to the expansion of a clone of mutated stem cells. Thus, after tumor initiation, stem cell overpopulation should be observed. The hypothesis of stem cell overpopulation was originally developed from a mathematical model of colon tumorigenesis [59], in which stem cell overpopulation was found to be the key event at the cellular level that links the initiating molecular event (an *APC* mutation) to the earliest tissue abnormality, a proliferative change in the mutant colonic crypts of FAP patients [60]. Stem cell overpopulation not only initiates colon tumorigenesis, but also drives tumor growth [61,62]. The study of the expression of markers for crypt base cells (i.e., putative stem cell markers) provided direct biological evidence in favor of the stem cell overpopulation hypothesis [63]: in fact, immunohistochemical studies during adenoma development in familial adenomatous polyposis provided evidence of an expansion of the stem cell crypt base cell population. In line with these observations, the percentage of LGR5^+^ cells was markedly higher in adenomas than in normal colon mucosal crypts [64,65].

Baker and coworkers have analyzed the spatial distribution of LGR5+ cells at the level of various adenomatous lesions showing that conventional adenomas display extensive expression of LGR5 and this expression is no longer restricted to the base of adenoma crypts; in contrast, in hyperplastic polyps and in serrated lesions, the basal localization of LGR5^+^ cells is retained, although their number is increased [66]. These findings may reflect differences in the origin and progression of these two pre-cancerous lesions [66]. These findings were confirmed in another recent study [67]; in addition, in this study, it was observed that in serrated lesions other stem cell markers, such as EPHB2 and OLFM4, were distributed in a diffuse manner [67]. In addition to LGR5^+^ cells, the adenomatous epithelium also displayed an increased proportion of ALDH1-positive cells [68]. While in the normal colon positive cells, stem-like markers were shown in the base of normal crypts, in the adenomatous epithelium, the cells positive for stem-like markers were expressed as a patchy destruction on the surface of adenomatous crypts [68].

In line with these observations on the distribution of LGR5^+^ cells in various types of adenomas, Shih and coworkers reported that the dysplastic cells present at the top of the crypts displayed genetic alterations (*APC* mutations) and neoplasia-associated patterns of gene expression. In contrast, cells located at the base of the crypts did not contain neoplasia-related genetic alterations and are not clonally related to the contiguous adenomatous cells [69]. According to these observations, a theory of development of colon adenomas following a top-down mechanism was proposed [69]. In contrast, in patients with a familial predisposition to colon polyposis, the dysplastic cells occupy entire single crypts (monocryptal adenoma): this observation implies a bottom-up model in which the stem cells present at the bottom of the crypt represent the cells responsible for tumor initiation [70]. Thus, it was proposed that polyps in familial adenomatous polyposis are initiated by and expand through crypt fission. The crypt fission process implies the division of a single crypt into two daughters. This process is particularly active in the early postnatal life, is well detectable in young individuals, and decreases with age. Crypt fission is driven by an expansion of the stem cell pool and is reactivated in cancer. Crypt fission is initiated at the level of the regions of the intestinal stem cell niches rich in Paneth cells, separated by a cluster of LGR5^+^ cells [71].

LGR5 expression was still higher in colon carcinomas than in adenomas. Furthermore, other recent studies have shown that LGR5 expression is essential for promoting the survival of human intestinal adenoma cells: in these cells, LGR5 expression is enhanced by prostglandin E2 (PGE2)—PGE2 levels are elevated in most intestinal adenomas due to cyclo-oxygenase 2 overexpression; in turn, PGE2 stimulates Wnt activation [72]. Since in FAP patients, non-steroidal anti-inflammatory drugs cause adenomas to regress, it is tempting to suggest that this effect could be mediated through the lowering of PGE2 levels, which in turn reduce LGR5 expression and the survival of LGR5^+^ adenoma stem cells [72].

### 2.3. Hereditary Syndromes Associated with Frequent Colorectal Cancer

In addition to FAP caused by germline mutations of the *APC* gene and characterized by hundreds to thousands of adenomatous polyps in the colorectum, other colon adenomatous syndromes are related to the germline mutations of other genes (Table 1). Particularly, MUTYH-associated polyposis (MAP) is an autosomal recessive inherited disorder caused by germline mutation of both alleles of the *MUTYH* gene (also known as *MYH* gene), encoding a DNA glycosylase involved in the base excision repair of the mismatches caused by oxidative DNA damage [73]. Both truncating as well as missense germline variants are frequently observed in MAP patients. Patients with MAP display a phenotype similar to that observed in patients with attenuated FAP, with a number of colorectal adenomas comprising between 10 and a few hundred [73]. It accounts for about 15–20% of cases of APC-negative adenomatous polyposis. MAP patients have an approximately 28-fold increased lifetime risk to develop colorectal cancer compared to the general population and the disease penetrance at 60 years varies from 43 to 100%.

Another form of familial polyposis, called polymerase proofreading-associated polyposis (PPAP) is caused by monoallelic germline mutations in *DNA polymarase E subunit* (*POLE*) and *POLD1* affecting the encoded exonuclease (proofreading) domain of DNA polymerases ε and δ [74]. In mutants, proofreading activity is lost, while polymerase activity is maintained. Mutation carriers’ tumors are MSS but tend to acquire base substitution mutations [74]. Furthermore, hypermutant, microsatellite-stable colorectal cancers seem to be caused by somatic *POLE* exonuclease domain mutations [74]. Pathogenic somatic mutations are observed in 1% of colorectal cancers and are mutually exclusive with a mismatch repair deficiency; these tumors usually display increased CD8^+^ lymphocyte infiltration [75]. A recent study identified homozygous germline nonsense mutations in the base-excision repair gene *NTHL1* in patients with adenomatous polyposis *APC* and *MUTYH*-wild-type. In one of the affected members, progression to colorectal cancer was observed; furthermore, the affected women developed an endometrial malignancy [76]. The affected tumors showed a mismatch repair-proficient and non-hypermutated profile, enriched for cytosine-to-thymine transitions [76].

Finally, another set of germline mutations favors the development of colon cancer, the so-called Lynch syndrome (LS). LS (also known as hereditary nonpolyposis colorectal cancer, HNPCC) is an autosomal, dominantly inherited syndrome caused by germline mutation in one of the mismatch repair genes, *MLH1*, *MSH2*, *MSH6* and *PMS2* or the *EPCAM* gene (Figure 3) [77]. Because of this genetic defect, LS tumors (colorectal cancers) are characterized by microsatellite instability [77]. It is the most common hereditary syndrome causing colorectal cancer (CRC), with about 2–5% of all CRCs caused by LS. Lynch syndrome is characterized by a high penetrance, early-onset colorectal cancer and an increased risk of extra-intestinal cancers. Patients with LS develop few colon adenomas, rapidly developing to carcinomas; in addition to colorectal cancer, patients with LS have an increased risk for many extracolonic malignancies, particularly endometrial cancer. The clinical presentation of patients with LS is variable, depending on the *MMR* gene affected in the germline: patients with *MLH1* and *MSH2* mutations have classic LS, while patients with *PMS2* mutations have a later clinical presentation.

Hereditary mixed polyposis syndrome (HMPS) is a rare condition characterized by the development of mixed-morphology colorectal tumors and is determined by a 40 Kb genetic duplication, resulting in the aberrant expression of the gene-encoding mesenchymal bone morphogenetic protein antagonist, GREM1 [78]. Epithelial GREM1 expression disrupts intestinal morphogen gradients, altering the normal cell fate of the intestinal crypt: thus, LGR5^+^ cells present outside the stem cell niche reacquire stem cell properties, proliferate and form ectopic abnormal crypts [79].

Serrated polyposis is a clinically defined syndrome characterized by multiple serrated polyps in the colo-rectum and an increased risk of developing colorectal cancer, with some unknown underlying genetics. The serrated polyps have a flat morphology and are difficult to detect with routine imaging procedures. Sessile serrated adenoma/polyp is the main precursor lesion of the serrated pathway, in which the BRAF mutation can lead to colorectal cancer with the high MSI, CIMP-high or MSS, CIMP-high phenotype. It was estimated that about 15% of all colorectal cancers arise through the serrated neoplasia pathway.

The identification of various cancer susceptibility genes and of their germline mutations in some individuals developing CRC allowed the evaluation of the impact of these genes on the occurrence of CRCs in large sets of patients. Thus, Yurgelund et al. evaluated the impact of 25 genes associated with inherited cancer risk in 1058 individuals with CRC. About 10% of these 1058 partecipants carried one or more pathogenic germline mutations, including 3.1% with Lynch syndrome, 7% with non-LS gene mutations, including 2.2% with mutations in high-penetrance genes (*APC*; biallelic *MUTYH*), and 3.6% with moderate-penetrance CRC risk gene mutations (monoallelic *MUTYH*, *APCI1307K*, *CHEK2*) [80]. A similar study carried out on early-onset colorectal cancers showed a 16% frequency of cancer susceptibility gene mutations [80].

### 2.4. The Serrated Pathway

Serrated polyps, previously called hyperplastic polyps, are a heterogeneous family of neoplasms, characterized by the characteristic saw tooth morphology, but differing in their malignant potential and molecular profile; in fact, this group includes both the hyperplastic polyps and serrated adenomas; serrated adenomas can be subdivided into the traditional serrated adenoma and the sessile serrated adenoma/polyp (Figure 3).

The majority of colorectal tumors follow a conventional pathway that is initiated by activating mutations of the WNT pathway; however, 10–15% of colorectal cancers are believed to initiate via activating mutations in the BRAF oncogene, which amplifies MAPK signaling and drives the serrated neoplastic pathway to colorectal cancer (Figure 3). While a single activating mutation of WNT/β-catenin pathway is sufficient to trigger the neoplastic transformation of intestinal stem cells, the BRAF mutant transgene promotes either the senescence or differentiation of intestinal stem cells [81,82]. The oncogenic activation of BRAF can eventually lead to tumor generation in mouse models, but with a slow kinetics of tumor development [83]. In fact, concurrent *CDX2* silencing, combined with *BRAF^V600E^* expression in the mouse intestinal epithelium, determine the development of intestinal tumors, including carcinomas and the tumors recapitulate histological features of human serrated morphology colorectal carcinomas [84]. The capacity of loss of *CDX2*—a differentiation-inducing transcription factor—to cooperate with the BRAF mutant to promote intestinal cell transformation is not surprising since *CDX2* abnormalities have been associated with BRAF-driven serrated tumor development [85,86,87]. A recent study better clarified the cellular and molecular contexts in which mutant *BRAF* is able to drive the oncogenic transformation of intestinal stem cells. In fact, it was shown that *BRAF* activation promotes the differentiation of the human epithelium, intestinal stem cell loss, and inefficient oncogenesis [88]. However, in genetic backgrounds associated with reduced levels of CDX2 or SMAD4, intestinal cell homeostasis is restored, stem cell activity is rescued and an oncogenetic process is driven [88]. In line with these observations, in human patients, reduced levels of differentiation in normal tissue are associated with increased susceptibility to serrated colon tumors [88].

Other studies based on animal models have further contributed to clarifying the molecular pathogenesis of serrated carcinomas. In fact, it was shown that the transgenic expression of oncogenic BRAF in mouse intestine cells induced serrated hyperplasia, but unexpectedly also induced the depletion of the intestinal stem cell pool, a mechanism regarded as a tissutal protection against oncogene activation [83]. Transgenic expression of stabilized β-catenin, together with oncogeneic BRAF, prevented intestinal stem cell loss [83]. Interestingly, BRAF^V637E^ knock-in mice allowed to demonstrate a reduced intestinal stem cell pool during the serrated hyperplastic stage but an increased number of intestinal stem cells in dysplastic lesions, were characterized by additional mutations that activated the WNT/β-catenin pathway [83]. The progression of serrated adenomas to cancer requires the overcoming of the suppressor activities of p53 and p16. A recent study reported a mouse model of colon serrated adenomas based on the development of conditional BRAF V637E (the equivalent in the mouse of the human V600E mutation): these mice developed serrated polyps, characterized by hyperplasia that, with time, progressed to dysplasia [89]. In 16% of the mice, the dysplasias progressed to invasive carcinomas [90]. However, cancers developed in these mice with a long latency: the introduction of p53 mutation or p16 inactivation in these mice accelerated cancer progression [89].

Aberrant crypt foci (ACF) are the earliest morphologically identifiable lesions observed in the human colon. All the observations suggest a direct association between the presence of recurrent adenomas and the presence of ACF, but the role of ACF as a precursor lesion of adenomas still remains unclear. ACF are not detectable through routine conventional endoscopy due to their small size (usually, <5 mm in diameter). High-definition chromoendoscopy is a suitable technique to identify colonic ACF, allowing their biopsy, microdissection and subsequent cellular and molecular analysis [90]. The analysis of BRAF and KRAS mutations in hyperplastic ACF provided evidence that the BRAF^v600E^ mutation was frequent among ACF with serrated morphology; conversely, KRAS mutations were rare in serrated lesions, but frequent in non-serrated lesions [91]. Recently, ACF were identified in the proximal colons of about 40% of individuals undergoing high-resolution chromoendoscopy and more frequently in patients with synchronous proximal adenomas; somatic mutations of APC, BRAF, KRAS, NRAS and ERBB2 were detected in 37% of proximal ACF [92]. These observations suggest that ACF may acquire molecular and histological changes representative of and seemingly predicting their probability to develop more advanced neoplasia. Particularly, APC mutations were observed only in ACF with histologic dysplasia, while mutations to MAPK signaling components—such as BRAF, KRAS, NRAS and ERBB2 mutations—were observed in hyperplastic ACF; MSI was specifically associated with hyperplastic ACF. These findings suggest the existence in ACF of two distinct signaling pathways involved in colorectal carcinogenesis: the traditional adenoma–carcinoma sequence and the alternate serrated pathway. Given the very early stage of ACF lesions, it is reasonable to assume that the two different pathways of colon carcinogenesis already diverged at the earliest stages of tumor initiation [92].

## 3. Somatic Genetic Abnormalities in Colon Cancer

### 3.1. General Studies

Colorectal cancer is a highly heterogeneous disease that comprises different tumor phenotypes. The classification of colon cancer according to major carcinogenetic pathways identified three different categories: (i) chromosomal instability tumors; (ii) microsatellite instability tumors; (iii) CpG island methylation phenotype tumors. These three different groups correspond to peculiar biological, clinical and pathological features of colorectal cancer [93].

Chromosomal instability is the most frequent cause of acquired genetic alterations occurring in colorectal cancers. Chromosomal instability (CIN) is characterized by widespread numerical chromosomal aberrations, subchromosomal aberrations, amplifications and loss of heterozigosity. One of the major negative results of CIN consists of the loss of tumor suppressor genes. Pathogenetic mutations occur at the level of key genes involved in colorectal cancerogenesis, such as *APC*, *KRAS*, *PI3KCA*, *SMAD4* and *TP53*. The frequency of these mutations and their possible impact on tumor development will be outlined in detail below. It is important to note that the meta-analysis of many studies involving the treatment of thousands of colon cancer patients showed that CIN was associated with a worse prognosis [94]. It is of interest to note that the loss of heterozygosity at the long arm of chromosome 18 is the most frequent cytogenetic abnormality observed in colon carcinoma.

Microsatellite instability is characterized by somatic alterations in the specific areas of the genome containing microsatellites (short sequences of nucleotide bases, repeated multiple times). The MSI group of colon cancer involves about 15% of colorectal cancers and is characterized by variable, altered lengths through insertions or deletions of repeated nucleotide sequences (microsatellites). In this tumor subtype two mutations are frequently observed at the level of *TGFβR2* and of the *activin receptor type 2*. Other frequent mutations are observed at the level of the *BAX* and *Caspase 5* genes. Deficiencies in the mismatch repair mechanism represent the key mechanism responsible for MSI. The inactivation of the MMR mechanism results from germline mutations (about 20% of cases) or epigenetic silencing (about 80% of cases) of components (*MLH1, MSH2, PMS2, MSH6*) of the MMR machinery. MSI patients have a better prognosis than microsatellite stable patients.

Epigenetic mechanisms play a key role in the genesis of the CpG island methylator phenotype. CpG clusters are normally present as clusters at the level of some promoter regions and are usually unmethylated. When these CpG islands are methylated, usually at the level of tumor suppressor genes, the transcriptional inactivation of these genes is observed. Two patterns of CpG island methylation have been reported in colorectal cancer: low-level of methylation increasing with age and high-level of methylation of a peculiar subset of CpG islands, resulting in gene silencing. CIMP tumors, and particularly CIMP high tumors, form a distinct subgroup characterized by peculiar clinical and pathologic features, that is, by a molecular phenotype characterized by wild-type (WT) *P53*, MSI and mutated *BRAF*.

More recently, Jass and coworkers proposed a classification of colon cancer based on five main criteria: CIN; MSI; CIMP, methylation status of 0–6-island methylguanine DNA Methyltransferase (*MGMT*); mutational status of *KRAS* and of *BRAF* [95]. According to these criteria, five subgroups were proposed: (i) the largest group including CIMP^−^, chromosomally unstable, MSS colorectal cancers (57% of cases); (ii) CIMP low, *KRAS*-mutated, *MGMT*-methylated, MSS/MSIlow (about 20% of cases); (iii) CIMPhigh, *BRAF*-mutated, MSI high (about 12% of cases); (iv) CIMP high, *BRAF*-mutated, chromosomally stable, MSS/MSI low (about 8% of cases); and (v) CIMP^−^, *BRAF* wt, chromosomally stable, MSI high (about 3% of cases) [95]. These various subgroups have been analyzed for their possible prognostic impact, providing evidence that three major subgroups can be identified, whose prognosis progressively improves: (i) *BRAF*-mutated; (ii) CIMP-high and CIMP-low (43%); and (iii) CIMP^−^ (50%) [96]. It is important to note that *BRAF* mutations are strongly associated with CIMP-high cancer which, in turn, correlates strongly with MSI-high cancer.

As mentioned above, some studies have supported the concept that proximal and distal colon cancers should be considered separately in etiological and pathogenetic studies and should be considered as two different entities. This two-colon concept was proposed as a dogmatic view and needed to be supported by various types of experimental evidence. Clinical studies have failed to convincingly demonstrate that the prognosis of proximal and distal colon cancers is different. A recent study based on the analysis of molecular markers in two large cohorts of patients provided some evidence in favor of a regional distribution of molecular abnormalities in colon cancer; particularly, the frequencies of CIMP-high, MSI-high and *BRAF* mutations increased in a statistically linear fashion from the rectum to the ascending colon [97]. Interestingly, this analysis also showed that cecal cancers seem to represent a peculiar subtype, characterized by a high frequency of *KRAS* mutations [97].

Many studies have contributed to identifying the key genes or pathways playing a crucial role in the initiation and progression of colorectal cancer; these include WNT, RAS-MAPK, PI3K, TGF-β and DNA mismatch-repair pathways [98].

**Figure 4:**
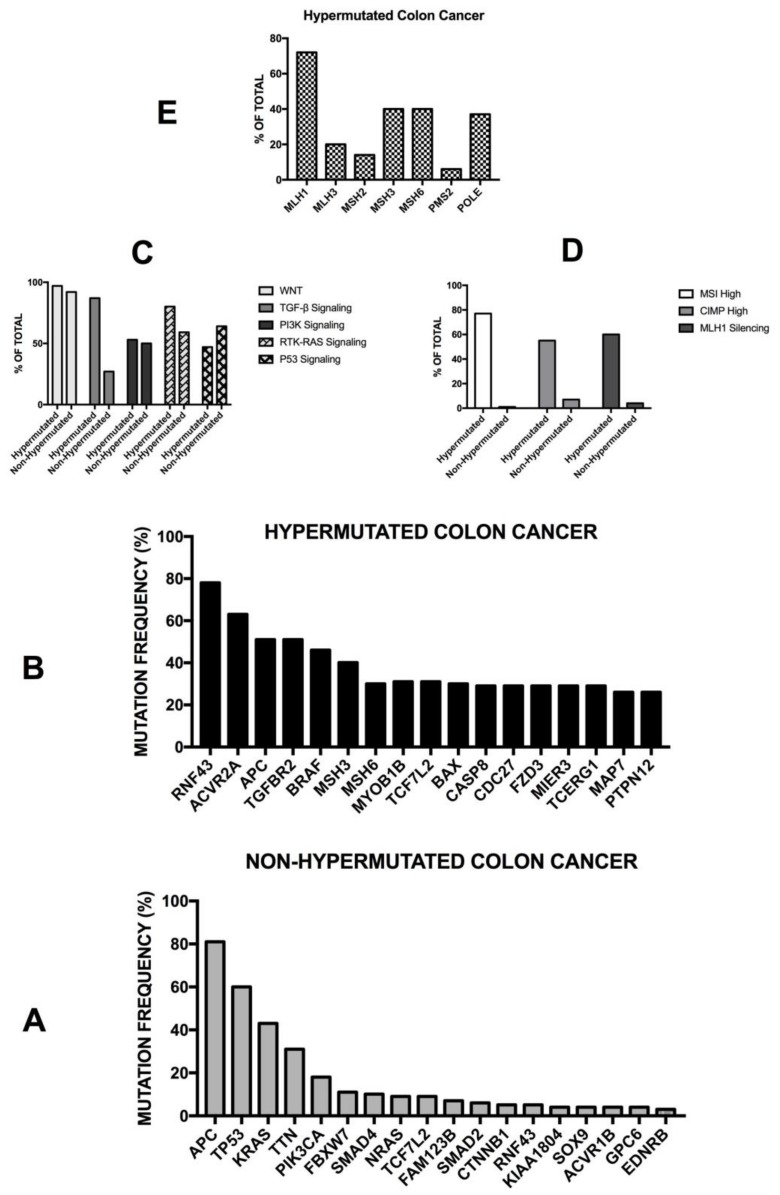
Genes most frequently mutated in colorectal cancer. (**A**) Frequently mutated genes in non-hypermutated colon cancer. (**B**) Recurrently mutated genes in hypermutated colon cancers. (**C**) Frequency of signaling pathway alterations observed in hypermutated and non-hypermutated colorectal cancers. (**D**) Frequency of microsatellite instability (MSI-High), CpG island methylator phenotype (CIMP-High) and *MLH1* gene epigenetic silencing in hypermutated and non-hypermutated colorectal cancers. (**E**) Mutations in mismatch repair genes and POLE among the hypermutated colorectal cancers. The figure shows the data reported in the The Cancer Genoma Atlas (TCGA) study [99].

The Cancer Genome Atlas (TCGA) recently provided a first comprehensive molecular characterization of human colon and rectal cancers (Figure 4) [99]. The genome-scale analysis of a large number of colon cancer samples provided several interesting findings: (i) 16% of colon cancers were hypermutated (about 75% of these exhibited high microsatellite instability, associated with hypermethylation and *MLH1* silencing, while the remaining 25% showed mutations of polymerase epsilon and mismatch repair genes); (ii) the non-hypermutated colon cancers displayed a consistent pattern of genomic mutations, involving 24 frequently mutated genes (including *APC, TP53, SMAD4, PI3KCA, KRAS, SOX9, FAM123B* and *ARID1A*); (iii) in the hypermutated cancers, *ACVR2A* (63%), *APC* (51%), *TGFBR2* (51%), *BRAF* (49%), *MSH3* (46%), *MSH6* (40%), *MYO18* (31%), *TCF7L2* (31%), *CASP8* (29%) were frequently mutated; (iv) two genes that were frequently mutated in the non-hypermutated cancers were significantly less frequently mutated in hypermutated cancers—*APC* (81% vs. 51%) and *TP53* (60% vs. 20%) (Figure 4). Importantly, the hypermutated tumors can be subdivided into two subgroups: an ultramutated subgroup (corresponding to 3% of all tumors), characterized by the presence of a mutation that inactivates the proofreading function within the exonuclease domain of the polymerase E DNA replicating enzyme; and a hypermutated subgroup (corresponding to 13% of all tumors), characterized by MSI. In addition to these mutations, recurrent alterations are represented by amplifications of *ERBB2, MYC* and *IGF2* and chromosomal translocations involving the fusion of *NAV2* genes with the WNT pathway member *TCF7L1* [99]. It is important to note that 93% of hypermutated cases had a deregulated WNT signaling pathway [99]. The TGF-β signaling pathway is more frequently altered in hypermutated (87%) than non-hypermutated (27%) tumors. Similarly, the rat sarcoma/receptor tyrosine kinase (RAS/RTK) signaling pathway is more mutated in hypermutated (80%) than in non-hypermutated (59%) tumors. The TCGA study reported the identification of three transcritpomic subtypes of colon cancer, called (i) microsatellite instability/CpG island methylator phenotype (MSI/CIMP); (ii) invasive; and (iii) chromosome instability. A subsequent analysis of the proteomes of colorectal cancers, characterized previously by TCGA, identified five proteomic subtypes, identified as A to E [99]. All hypermutated and MSI-high tumors pertain to the B and C subtypes—subtype B tumors are associated with the TCGA CIMP-high methylation subtype and lack TP53 and chromosome 18q loss, and the C subtype is significantly associated with a non-CIMP subtype. The other subtypes—A, D and E—are associated with CIN; in particular, subtype E is significantly associated with TP53 mutations and 18q loss and with HNF4A amplification (and higher abundance of HNF4A protein) [99]. Copy number alterations were frequent, particularly among non-hypermutated, MSS tumors; although these copy number alterations show strong cis and trans effects on the mRNA level, and only a few of them also extend to the protein level. Interestingly, the chromosome 20q amplicon is associated with the largest changes at the mRNA and protein level and is associated with *HNF4* (hepatocyte nuclear factor 4, alpha), *TOMM34* (translocase of outer mitochondrial membrane 34) and *SRC* (SRC proto-oncogene, non-receptor tyrosine kinase) overexpression [100].

Other recent studies have led to the identification of other recurrent genetic alterations occurring in colon cancer. Among them, particularly relevant are the recurrent gene fusions involving the genes encoding either *R-Spondin2* or *R-Spondin3* and occurring in about 10% of colon cancer [90]. Interestingly, spondin fusions are mutually exclusive with *APC* mutations, thus suggesting that they had a role in the activation of Wnt signaling and tumorigenesis [101]. In line with this interpretation, the spondin fusion proteins consistently potentiate the Wnt signaling [101].

Genomic sequencing of colorectal adenocarcinomas has identified a recurrent *VTIA-TCF7L2* fusion, occurring in about 3% of patients [102]. The *TCF7L2* gene encodes TCF4, a tumor suppressor gene which cooperates with β-catenin in colorectal carcinogenesis [103]. Recently, Kloosterman and coworkers have performed a systemic analysis of oncogenic gene fusions in 278 primary colon cancers [103]. Overall, 2.5% of all tumor samples were defined as harboring a relevant gene fusion, corresponding to kinase fusions in 1.8% of patients. RSPO2, NTRK3, ERAS and BRAF were observed [103]. R-spondin fusions were more rarely detected than in previous studies. Interestingly, this study reported a novel fusion involving USP9X-ERAS formed by chromotrypsis and determining a high expression of ERAS, a constitutively active RAS protein, normally expressed only in embryonic stem cells [103].

As mentioned above, the TGF-β family is frequently mutated in colorectal cancers. This pathway involves two membrane receptors—TGFBR1 and TGFBR2—which are activated on the binding of their TGF-β ligand, with the consequent phosphorylation of the receptor-associated SMAD2 and SMAD3; in turn, the activated SMAD2 and SMAD3 bind the common mediator SMAD4, with the consequent relocation of this molecular complex at the level of the nucleus, resulting in the final regulation of target genes. All the different members of the TGF pathway are frequently mutated in colorectal cancers. In sporadic colorectal cancers, the *TGFBR2* and *SMAD4* mutations are found in about 15% and 10% of patients, respectively. It is important to note that TGFBR2 mutations are particularly prevalent in microsatellite unstable tumors, with about 80% of these tumors harboring frameshift mutations at the poly-adenosine tract in the exon 4 [104]. Recently, the frequency of mutation of all *SMAD* members was investigated in a large cohort of patients: the frequency of *SMAD* mutations was 8.6%, *SMAD2* 3.2% and *SMAD3* 4.3%. The large majority of these mutations were predicted to be mutagenic, since they were predicted to reduce protein stability. *SMAD2* and *SMAD3* mutations were mutually exclusive with *SMAD4* mutations [105]. The biological consequences of reduced/inactivated TGF-β signaling in colorectal cancers are unclear. A simple interpretation would be that mutations of *TGF-β receptor* prevent signaling to SMAD proteins and the loss of TGF-β-mediated transcriptional activity prevents cells from responding to TGF-β signals, inhibiting cell proliferation. However, although it is evident that alterations of the TGF-β signaling or a restoration of TGF-β signaling could play a beneficial effect on colorectal cancer growth, the complexity and the heterogeneity of the response of colorectal cancer cells to a stimulation or inhibition of TGF-β signaling is demonstrated by the study of some experimental models. In tubular adenoma organoids, TGF-β activation induced apoptosis, consistent with its role in suppressing tumor growth; the expression of *BRAF^V600E^* mutant into tubular adenoma organoids, mimicking the serrated adenoma pathway, changed the response to TGF-β from apoptosis to the epithelial to mesenchymal transition [106]. These observations showed the variability of the response of colorectal cancer cells to TGF-β and indicate that TGF-β drives the transformation of sessile serrated adenomas into the mesenchymal subtype 4 colorectal cancer [106].

### 3.2. PIK3CA Mutations

As mentioned above, *PIK3CA* mutations are frequent in colon cancer. In fact, mutations of the *PI3KCA* gene are present in approximately 15–20% of colon cancer patients and the activation of the PI3K pathway plays an important role in colon carcinogenesis. Studies that use pyrosequencing assays generally show higher frequencies of *PI3KCA* mutations than Sanger sequencing studies. The prevalence of *PI3KCA* mutations in colon cancer increases continuously from the rectum (10%) to the cecum, supporting the colorectal continuum paradigm. Activation of PI3K signaling enhances prostglandin-endoperoxide synthase 2 (PTGS2) activity and prostaglandin E2 synthesis, determining the inhibition of apoptosis in colon cancer cells. Aspirin may suppress colon cancer cell growth and induce apoptosis by blocking the PI3K pathway. In vitro studies have shown that aspirin induces the apoptosis of colon cancer cell lines bearing PI3KC mutations (3140A > G or 1633G > A) and this effect was clearly more pronounced in these cells than in colon cancer cells with the PI3KC wild-type [107]. In line with this hypothesis, a recent clinical study based on the analysis of a large set of patients clearly showed that aspirin administration resulted in an improvement of survival selectively among patients with *PI3KCA* mutations [108]. In spite of this effect of aspirin administration, the incidence of *PI3KCA* mutations was similar in both colon cancers that arose in aspirin users compared with aspirin non-users prior to diagnosis. Given these observations, several clinical trials of aspirin, as well as of the PTSG2 selective inhibitor Colecoxib, for colorectal patients are underway. The results of trials performed in 2419 colorectal cancer patients diagnosed between 1997 and 2008 were recently published, providing clear evidence that the administration of aspirin or any nonsteroidal anti-inflammatory after tumor diagnosis was associated with an improved overall survival among participants with KRAS wild-type tumors, but not among tumors with KRAS-mutant tumors [109]. In this study, the best benefit was associated with aspirin-only use [109]. The benefic effect induced by aspirin does not seem to be related only to its anti-inflammatory and direct anti-tumor effects, but also to the activation of T-cell-mediated anti-tumor immunity; in fact, Hamada and coworkers observed in the context of the Nursey’ Health Study and Health Professionals Follow-Up Study that the survival benefit associated with aspirin administration is stronger in colorectal cancers with a lower CD274 (PD-L1) expression [110]. These observations suggest a differential antitumor effect of aspirin according to immune checkpoint status [110].

It is important to note that the molecular and clinical disease of colon cancers with *PI3KCA* mutations is heterogeneous in that three categories have been observed: patients with exon 9 mutation; patients with exon 20 mutations; patients with both exon 9 and exon 20 mutations [111]. The first two categories of patients displayed a prognosis comparable to that of patients with wild-type *PI3KCA*, while patients with double mutations have a poor prognosis [110]. *PI3KCA* mutation in colorectal cancer is associated with phosphorylated AKT expression, *CTNNB1* inactive status and *KRAS* mutations. Particularly, in a study of NGS of 206 metastatic colon cancers, concomitant *KRAS* mutations were seen in 51% of *PI3KCA*-mutated tumors; furthermore, concomitant mutations of *KRAS, BRAF* or *NRAS* mutations were detected in 82% tumors with a *PI3KCA* exon 9 mutation and 58% of tumors with a *PI3KCA* exon 20 mutation [112]. For stage II–III colon cancer, *PIK3CA* mutation was significantly associated with tumor recurrence and poor survival [113]. Very interestingly, Cohen and coworkers recently reported a high frequency of *PIK3CA* mutations in a subset of colorectal cancer patients defined as double somatic mismatch repair mutations [114]. Double somatic describes cases with at least two somatic alterations in mismatch repair genes, which results in a molecular phenotype that mimics Lynch syndrome cancers; however, at variance with the Lynch syndrome cancers, the double somatic cancers have no detectable germline mutations in the *MMR* genes [114]. Among colorectal cancers, double somatic cases have a frequency of *PI3KCA* mutations much higher than Lynch syndrome, *MLH1* hypermethylated and MSS tumors (Figure 4) [114]. These findings were validated in a cohort of colorectal patients from TCGA [114].

### 3.3. BRAF Mutations

*BRAF*^V600E^ mutations are observed in about 10% of colon cancer patients. The frequency of BRAF mutations was markedly higher in hypermutated (47%) than in non-hypermutated (3%) colon cancers. A systematic review and meta-analysis of many clinical studies carried out in the last decade clearly showed that *BRAF* mutations in colon cancer were associated with a more than two times higher risk of mortality [115]. More recent studies have confirmed the reduced overall survival in metastatic *BRAF*-mutated colorectal cancers (18.2 months), compared to that observed in metastatic *BRAF*-WT colorectal cancers (41.1 months) [116]. However, for patients with early-stage colorectal cancers, the overall survival between *BRAF* mutant and wild-type cancers differed non-significantly [117]. Interestingly, the frequency of *BRAF* mutations changed considerably in the various tumor sites: in fact, it markedly increased from the rectum to the ascending colon [97,118]. At variance with melanoma, colon cancers harboring *BRAF* (V600E) oncogenic lesions are not responsive to treatment with vemurafenib [119]. In colon cancer cells possessing *BRAF* (V600E) mutations, only simultaneous BRAF and EGFR inhibition achieved a significant inhibitory effect on tumor cells [119]. The combination of a BRAF inhibitor with an EGFR inhibitor was recently tested in *BRAF*-mutated colon cancer patients with very modest therapeutic results [120]. In fact, the combined administration of RAF and EGFR inhibitors initially induces tumor regression in most patients, but acquired resistance invariably develops, typically within six months. In a recent randomized clinical trial of Cetuximab and Irinotecan—with or without Vemurafenib—in BRAF^V600E^ colorectal cancer patients (SWOG 1406), there was an improved response rate in the triplet arm, but this was associated with only modest levels of progression-free survival (4.4 months) [121]. Recent studies have explored the mechanism of resistance of BRAF^V600E^-mutated colorectal cancers to BRAF-inhibitors, showing that the mechanism of resistance implies alterations in elements of the RAS-RAF-MEK-ERK pathway. In this context, the genetic amplification of wild-type RAS is a recurrent mechanism of resistance, determining increased receptor kinase-dependent activation of RAS [122]. *RAS* gene amplification causes resistance to RAF/EGFR inhibition by increasing cellular RAS/GTP levels to levels sufficient to drive BRAF^V600E^ dimerization; in fact, currently approved RAF inhibitors inhibit RAF monomers, but not dimers [122]. The use of inhibitors able to block ERK signaling driven by both RAF monomers and dimers were able, in combination with an EGFR inhibitor, to suppress the growth in vemurefanib/cetuximab-resitant colorectal tumors [122]. Recently, a third class of BRAF mutants was described, corresponding to those that have impaired kinase activity or are kinase-died: these mutants bind more tightly than WT BRAF to RAS-GTP and their binding to WT CRAF is enhanced, leading to increased ERK signaling; in colorectal cancer with class 1 BRAF mutants, RAS is typically activated by receptor tyrosine kinase signaling [123]. Therefore, there are three types of BRAF mutants: activating BRAF mutants cause feedback inhibition of GTP-bound RAS, are RAS-independent and signal as either monomers (class 1) or constitutively active dimers (class 2); class 3 mutants induce RAS-signaling dependent activation and are sensitive to ERK-dependent feedback [123]. BRAF mutations are strongly associated with a CIMP that determines the silencing of various genes, including tumor suppressor genes, containing CpG islands in their promoters [124]. About 20% of BRAF-mutant colorectal cancers display MMR/MSI-H, due to *MLH1* gene epigenetic silencing. MSI-H tumors are usually hypermutable, display thousands of DNA mutations, often contain many infiltrating T cells, and are candidates for immunotherapy. Clinical trials of immune checkpoint inhibitors have shown remarkable activity in patients with metastatic colorectal cancer with MSI-H, although responses are not dependent on the BRAF mutation status [125,126].

Interestingly, some colorectal cancer patients possess non-^V600^BRAF mutations, occurring in 2.2% of all patients tested and accounting for 22% of all identified BRAF mutations [127]. Tumors with non-^V600^BRAF mutations, compared with tumors with ^V600^BRAF mutations, were found in patients who had fewer high-grade tumors (13% vs. 64%, respectively) or right-sided primary tumors (36% vs. 81%, respectively); the median overall survival was significantly longer in patients with non-^V600^BRAF mutations compared with those with both ^V600^BRAF mutations and WT BRAF metastatic colorectal cancer (60.7 vs. 11.4 vs. 43 months, respectively). Non-^V600^BRAF mutations define a clinically distinct subtype of colorectal cancers with a good prognosis [127].

### 3.4. KRAS Mutations

*KRAS* is very frequently mutated in colon cancer, its mutation being detected in about 40% of cases; KRAS mutations are mutually exclusive of NRAS and BRAF mutations; in more than 90% of these cases *KRAS* mutations occur at the level of codons 12 and 13; more rarely, these mutations involve exon 3 and exon 4. The presence of *KRAS* mutations was associated with resistance to anti-EGFR therapy. The prognostic impact of *KRAS* mutation was recently analyzed in a large pool of colon cancer patient wild-types for the *BRAF* gene (to exclude from the analysis patients with mutated *BRAF*, who have a poor prognosis). This type of analysis provided evidence that codon 12 *KRAS* mutations, but not codon 13 *KRAS* mutations, are associated with a negative prognosis [128]. Numerous cell lines from *KRAS* mutated colon cancers have been isolated and analyzed for their dependency for growth/survival from mutated *KRAS*, showing that about 50% of these lines are KRAS-dependent [129]. The careful comparison of *KRAS*-dependent and *KRAS*-independent colon cancers led to the identification of *MAP3K7*, encoding the TGF-β-activated kinase (TAK1), as a driver of cell survival of *KRAS*-mutated-dependent, *APC*-deficient cells [130]. In these KRAS-dependent colon cancer cells, KRAS activates bone morphogenetic protein 7 (BMP-7) signaling, leading to TAK1 activation, β-catenin nuclear localization and transcriptional upregulation of Wnt target genes [130]. These cells are exquisitely sensitive to the blockade of TAK1 expression, indicating that this protein may represent an important potential target for these tumors [130]. *KRAS* mutated colorectal cancers are usually well/moderately differentiated tumors, are usually associated with classical adenocarcinoma subtype and usually have a microsatellite stable phenotype; *KRAS* exon 3–4 mutated colorectal cancers are more frequently associated with mucinous/rare histological subtypes [131]. The essential role of *KRAS* in colorectal carcinogenesis is supported by some animal models. Thus, mutant *KRAS* expression in the context of the *APC*-mutant colonic epithelium is sufficient to induce dysplastic adenocarcinomas [132]. More recently, Boutin and coworkers, using an experimental mouse model in which mutations in *APC, P53* and *KRAS* are spatially and temporally regulated, have shown that KRAS expression is required for tumor maintenance, even in a situation where KRAS activation is not an initiating event: loss of KRAS expression caused the apoptosis of primary and metastatic colon adenocarcinomas, while no apoptosis was induced in simple adenomas [133].

### 3.5. HER2 Mutations

About 7% of colorectal cancer patients have *HER2* somatic mutations or *HER2* gene amplifications. *HER2* activating mutations cause EGFR resistance in colorectal cell lines; patient-derived xenografts show durable regression when treated with dual *HER2*-target therapy with trastuzumab plus tyrosine kinase inhibitors [134]. A phase 2 clinical trial based on the dual HER2 blockade in CRC patients with *HER2*-positive tumors confirmed some clinical activity of this drug combination, with 4% of patients displaying a complete response and 26% a partial response to treatment [135].

Exome sequencing studies of colorectal patients have shown that in about 10% of these patients, mutations of the *AMER1* gene were observed; these mutations determine a loss of function of the *AMER1* gene [136]. The subsets of colorectal tumors lacking *AMER1* expression showed inhibition of the Wnt pathway and exhibited a mesenchymal phenotype [136]. No germline mutations of the *AMER1* gene were observed in patients with familial polyposis [137].

### 3.6. Molecular Subtypes

The various pathways of colorectal cancer development result in tumor subtypes that can be classified according to combinations of the main molecular abnormalities, including MSI, CIMP, CIN, BRAF mutations and KRAS mutations. In these studies, colorectal cancers can be subdivided into five different subtypes based on combinations of tumor markers: type 1 (MSI-H, CIMP^+^, positive for *BRAF* mutation and negative for *KRAS* mutation); type 2 (MSS or MSI-low, CIMP^+^, positive for *BRAF* mutation, negative for *KRAS* mutation); type 3 (MSS or MSI-low, non-CIMP, negative for *BRAF* mutation, positive for *KRAS* mutation); type 4 (MSS or MSI-low, non-CIMP, negative for both *BRAF* and *KRAS* mutations); and type 5 (MSI-H, non-CIMP, negative for both *BRAF* and *KRAS* mutations) [138]. Alternatively, colorectal cancer can be subdivided into five subtypes according to MRR status and detection of *BRAF* and *KRAS* mutations; three subtypes are MRR proficient—*BRAF*-mutant (about 7%), KRAS-mutant (about 35%) and lacking either *BRAF* or *KRAS* mutations (49%); and two subtypes are MMR-deficient—the sporadic type with BRAF mutation and/or hypermethylation of *MLH1* and the familial type, lacking *BRAF* mutations or hypermethylation of *MLH1* [139]. Most colorectal tumors develop according to a sequential pathway of development, including normal intestinal mucosa to adenoma to carcinoma. This pathway involves the accumulation of inhibitory mutations at the level of tumor-suppressor genes and activating mutations at the level of oncogenes and are associated with colorectal tumors exhibiting MSS, non-CIMP, CIN, absent *KRAS* and *BRAF* mutations, and *APC* mutations. These tumors usually have a better prognosis than *BRAF*-mutated colorectal cancers, but a poorer prognosis than MSI-high colorectal cancers [138,139]. About 20–30% of colorectal carcinomas develop via a serrated neoplasia pathway, thus defined according to the saw-toothed pattern of precursor polyp lesion; these tumors are usually *BRAF* or *N-KRAS*-mutated and are characterized by CIMP. Colorectal cancers exhibiting a serrated phenotype, characterized by mutated *BRAF*, CIMP^+^ and MSS status are associated with a very poor prognosis. However, colorectal patients with mutated *BRAF*, CIMP^+^ and MSI-high have a better prognosis than those with traditional adenoma-to-carcinoma tumor pathway tumors, thus indicating that even among patients with a serrated phenotype, there is a marked variability in clinical outcome, according to the MS status [138,139]. This observation strongly indicates the absolute need for a multimarker approach to classify colon cancer patients and to select them for optimal treatments. Another group of colorectal cancers is represented by *KRAS*-mutated, CIMP-low and MSS: these tumors seem to originate through an alternative pathway. Another typical feature of this group of tumors is *MGTM* promoter hypermethylation. It is unclear which precursor lesion is indicative of these tumors, but it is clear that they are associated with a poorer prognosis than the traditional, adenoma-to-carcinoma sequence [138,139]. Several recent clinical trials, carried out on large sets of colon carcinoma patients, have assessed the impact of MMR status and *BRAF/KRAS* mutations on outcome. In these studies, the patients were treated with the standard chemotherapy FOLFOX (folinic acid, fluorouracil, and oxaliplatin) without or with an anti-EGFR or anti-vascular endothelial growth factor (VEGF) agent. The deficient MMR phenotype is a favorable prognostic factor in patients with stage III colon cancer receiving standard FOLFOX adjuvant chemotherapy [140,141]. In resected stage III patients receiving adjuvant FOLFOX, *BRAF* and *KRAS* mutations are associated with shorter overall survival in patients with MSS, but not in those with MSI tumors [142]. In patients with a recurrence of stage III colorectal cancer, deficient MMR was associated with better survival, but this benefit was limited to patients with proximal tumors; mutations in BRAF were associated with worse survival and worse survival for *BRAF* or *KRAS*-mutant tumors was more strongly associated with distal tumors [143].

The development of studies characterizing the molecular abnormalities occurring in colorectal cancers raised the important problem of the minimal number of driver mutations required for the development of colorectal cancer. By using an approach that combines conventional epidemiological studies with genome-wide sequencing data, Tomasetti and coworkers reached the important conclusion that three sequential mutations are required to develop colon adenocarcinomas [144].

As mentioned above, about 6% of colorectal cancers have a familial germline genetic component: 3% are related to Lynch syndrome, 2% to familial adenomatous polyposis and 1% to other hereditary syndromes including the TACSTD1 deletion of *MSH2*. Lynch syndrome is caused by the germline mutation of one of the genes involved in the DNA nucleotide mismatch repair system—usually *MSH2* and *MLH1*—while most of cases of FAP are related to germline mutations of the tumor suppressor *APC* gene. Lynch syndrome is autosomal dominant with a high lifetime risk of about 60% of developing colorectal cancer. At variance with sporadic colorectal cancers with MSI, the cancer development in Lynch syndrome originates from an adenomatous polyp. Typical pathological features of colorectal cancer associated with Lynch syndrome consist in the presence of multiple colorectal cancers exhibiting typical MSI-high histology. At the molecular level, the Lynch syndrome is distinguished by sporadic MSI-high colorectal cancers because acquired CpG methylation of the *MLH1* promoter and V600E mutation of the *BRAF* gene are frequently observed in the latter, but not in the former. Lynch syndrome is caused by genetic defects at the level of MMR components, particularly by germline mutations of one of the *MMR* genes (*hMSH2*, *hMLH1*, *hPMS2*, *hMSH6*, *EPCAM*), followed by the somatic inactivation of the second allele. When the second allele becomes mutated, cancer can develop. More than 85% of germline mutations occur at the level of either *hMSH23* or *MHL1*; mutations at the level of *MSH6* occur in 7–8% and *PMS2* in about 1% of cases. More than 95% of the tumors developing in patients bearing *MMR* gene mutations exhibit MSI. MSI is typically characterized by a widespread instability occurring at the level of short repeat microsatellite sequences present in coding and non-coding regions, due to MMR deficiency. In the MSI pathway observed in Lynch syndrome, CRC formation is driven by the acquisition of mutations at the level of the coding sequences of genes, such as growth factor receptors (*TGFBR2* and *IGF2R*), genes involved in apoptosis (*BAX*) and DNA repair (*MSH3* and *MSH6*) [145,146]. Recent studies have shown that virtually all (99%) CRCs developing in Lynch syndrome patients display MSI-H; the most mutated genes exhibiting microsatellite repeats are *ACVR2A, TGFBR2, EGFR, BPMR2, E2F4, MSH3, BAX* and *TCF7L2* [147]. In these CRCs developed in Lynch syndrome patients *BRAF* mutations are absent, while *KRAS* are frequent (about 40%). Binder and coworkers recently performed a whole genome DNA-sequencing and RNA-sequencing analysis of LS-associated colorectal cancers [148]. The results of this study support the existence of two subgroups of LS-related colorectal cancers: G1, characterized by a higher number of somatic mutations, a higher amount of microsatellite slippage, a different mutational spectrum and a strong immune response associated with the expression of HLA and immune checkpoint genes and the invasion of CD4^+^ T lymphocytes; G2 tumors show weaker MS instabilities and fewer mutations. According to these findings, it was proposed that G1 tumors are programmed for escape from a highly immunogenic microenvironment, bypassing human leukocyte antigens (HLA) presentation and T-cell exhaustion, while G2 tumors develop in a less immunogenic microenvironment, where their progression is promoted by inflammation [148]. However, this subdivision of LS-associated colon cancers in two subgroups was based on the analysis of only a limited number of tumor samples and therefore must be validated through the analysis of a larger number of cases. Colorectal cancers developing in patients with LS are preceded by the formation of colon adenomas. A total of 79% of these adenomas display MMR-deficiency and 21% were MMR-proficient, while all LS-associated colorectal carcinomas are MMR-deficient [149]. Sequencing studies showed that LS-associated MMR-deficient adenomas display a lower frequency of *APC* or *CTNNB1* mutations (37%), compared to that observed in LS-associated MMR-proficient adenomas (72%) or in sporadic adenomas [149]. Frequent frameshift mutations of *RNF43* are observed in LS-associated adenomas [148]. Lynch-syndrome-associated adenomas display a mutational profile similar to that observed in LS-associated colon cancers [149].

The methylation profile of CRCs occurring in Lynch syndrome patients is different from that observed in sporadic MSI-H colon cancers: the latter display a typical hypermethylation pattern markedly higher than that observed in sporadic MSI-H colorectal cancers and comparable to that observed in sporadic MSS colorectal cancers [150]. Furthermore, about 30% of colorectal cancers occurring in Lynch syndrome patients display a hypomehtylation pattern and a negative prognosis [150].

**Figure 5:**
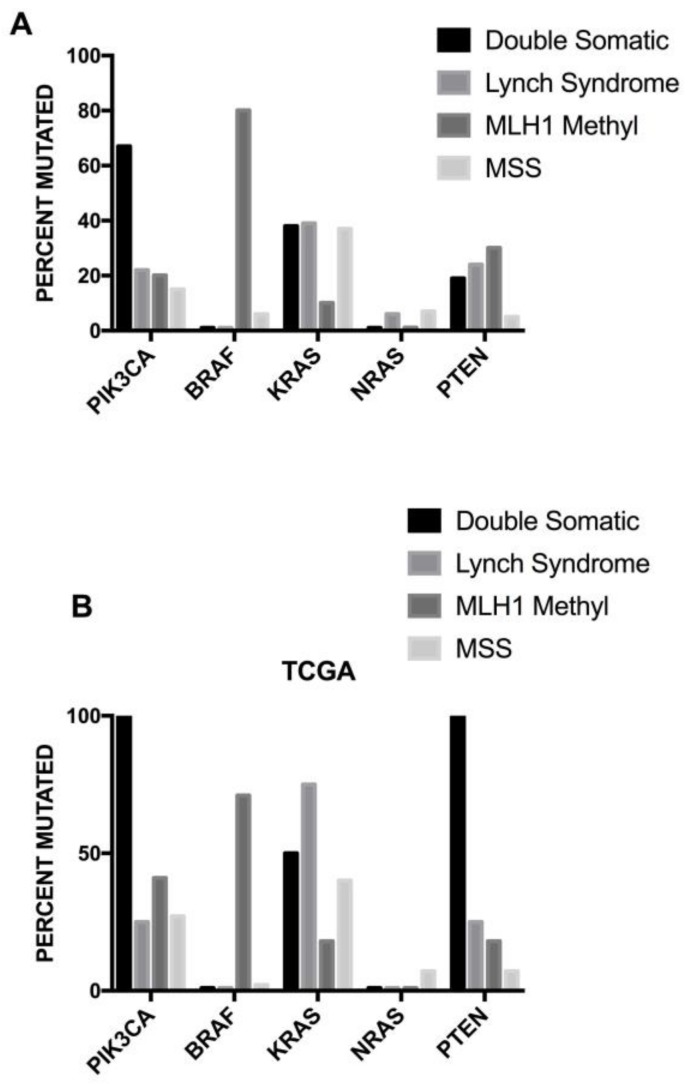
Distribution of somatic mutations in colorectal cancers subdivided into four tumor groups: double mutant (i.e., colorectal cancer samples containing two or more somatic mutations in genes encoding mismatch repair proteins), Lynch syndrome, MLH1-hypermethylated, microsatellite stability (MSS). The upper panel (**A**) shows the data reported by Cohen and coworkers [114] and the lower panel (**B**) the data reported in the TCGA study [99].

Recent studies have compared the mutational pattern of various types of colorectal cancers displaying high MSI. In particular, these studies compared various types of CRCs, including those occurring in Lynch syndrome patients, CRCs with MLH1 methylation, CRCs with double somatic alterations in mismatch repair genes (in these cases there no germline mutations in the MMR gene), compared to sporadic MSS colorectal cancers [110]. These three groups of CRCs are characterized by a high number of mutations, with the highest levels observed in double somatic and Lynch syndrome cancers (Figure 5). A high rate of BRAF mutations was observed only in the MLH1 methylated group; KRAS mutations were similar (around 40%) in the three MSI-H groups, but markedly lower in the MSS group; a high rate of PIK3CA mutations was observed selectively in the double somatic group (Figure 5).

### 3.7. Molecular Mechanisms of Resistance to Anti-EGFR Therapy

As mentioned above, anti-EGFR antibodies are used for the treatment of colorectal cancer. No univocal genetic determinants of sensitivity to EGFR inhibition have been identified. Retrospective studies based on the analysis of many trials suggest that colorectal cancer patients with a left-sided primary tumor (with RAS wild-type disease) exhibit improved median overall survival (mOS) and median progression-free survival (mPFS) following anti-EGFR therapy in the first-line setting. This effect appears to be independent of the higher rate of *BRAF* mutations in right-sided tumors. In contrast, a trend towards worsened outcomes for right-sided tumors when treated with anti-EGFR agents was noted. The peculiar dependency of colorectal cancer on EGFR signaling does not seem to be related to the presence of EGFR abnormalities present in these tumors, but to the peculiar function of this receptor in the colon physiology. EGFR plays a key role in the control of mucosal regeneration and inflammatory responses [151]. EGFR signaling increases the propensity to the oncogenic transformation of colon epithelial cells upon chronic inflammatory stimulation [151]. Multiple mechanisms are responsible for primary or acquired resistance to EGFR blocking therapy, related to three different mechanisms: (i) reduced antibody-receptor interaction; (ii) activation of parallel substitute pathways; and (iii) constitutive activation of downstream signaling pathways (related to the presence of *KRAS* or *NRAS* mutations, *BRAF* and *PIK3CA* mutations, *KRAS* amplifications) [151]. It is important to note that most of the genetic causes of EGFR resistance converge to determine hyperactivity of the MAPK extracellular signal-regulated kinase/extracellular signal-regulated kinase (MEK–ERK) axis; therefore, the inhibition of this signaling pathway may represent an important strategy to prevent or retard the emergence of resistance to EGFR inhibitors. In a clinical setting, of CRC patients who responded to treatment with anti-EGFR Cetuximab and Panitumumab (among *KRAS* wild-type tumors, only 12–17% patients had durable responses to anti-EGFR monotherapy), invariably developing resistance within several months after initial response. Attempts to target tumor cells after the already acquired resistance failed. Thus, recently, it a preventive approach aiming to prevent the development of resistance by treating colon cancer cells with EGFR inhibitors plus MEK inhibition was proposed: this combined treatment, evaluated in xenotransplantation models of colorectal cancer, showed that the combined treatment resulted in a more effective induction of apoptosis than single agents alone, and in a limitation of the emergence of resistant clones [152]. The exome analysis of a wide group of CRC patients undergoing EGFR blockade showed additional mechanisms of resistance in addition to those commonly reported, including mutations other than those commonly reported, such as mutations of *ERBB2*, *EGFR*, *FGFR1*, *PDGFRA* and *MAP2K1*; furthermore, amplifications and mutations in the tyrosine kinase receptor adaptor gene *IRS2* have been identified in tumors displaying sensitivity to EGFR blockade [153].

### 3.8. Molecular Abnormalities in Colitis-Associated Colorectal Cancer

Inflammatory bowel diseases, such as ulcerative colitis (UC) and Crohn’s disease (CD), increase the risk of developing colon cancer. Up to 15 years ago the risk of developing cancer in chronic inflammatory bowel diseases was considered to be high, in the order of 0.5–1% per year. After this time, a progressive decrease in the risk of cancer development was noted with actual estimates of 1.5 to 2 times higher risk than the general population of colorectal cancer development [154]. The genetic pathways of tumor development in UC and CD have not been conclusively determined. The analysis of genetic alterations indicates etiologic similarities, but also differences between UC and CD. UC-associated neoplasia frequently shows*TP53* or *KRAS* mutations, while mutations of the *APC* gene are rarely observed [155,156,157,158,159]. Loss of the heterozygosity of chromosome arm 18q, targeting the *SMAD4* gene, is also relatively frequent in UC [158]. These initial studies showed also that the same mutations in *KRAS, TP53* and *CDKN2A* genes are observed in neoplasia were also present in non-tumor, non-dysplastic and dysplastic epithelium [160]. This phenomenon corresponds to the co-called “field cancerization”, which consists in the reconditioning of a large area of histologically normal epithelium for the future development of neoplastic lesions. Field cancerization may help to explain the high frequency of synchronous and metachronous neoplastic lesions in patients with intestinal inflammatory bowel disease. The mechanisms though which inflammation promotes intestinal neoplastic transformation are unclear, but an obvious mechanism could be related to the induction by inflammation of repeated cycles of epithelial wounding and repair, a condition that favors the occurrence of spontaneous mutations; furthermore, the inflammatory microenvironment could provide a clonal advantage to the mutated clones able to rapidly repopulate the healing mucosa. However, whatever the mechanism is through which inflammation promotes intestinal neoplasia, it was clearly supported by observational studies that colon cancer risk is clearly linked to the severity, duration and extent of inflammation.

Two recent studies have explored the genetic landscape of colorectal cancer associated with inflammatory bowel disease (here defined as colitis-associated cancer, CAC). The first study carried out by Robles and coworkers resulted in a number of interesting observations. The number of somatic mutations of CAC cancers is comparable to that observed in sporadic, non-hypermutated colorectal cancers [161]. The comparison of the mutation rate of various genes observed in CAC, compared to those already reported for sporadic non-hypermutated CRC, showed similarities, but also many discrepancies: (i) the mutation frequency of genes such as *BRAF, PIK3CA* and *SMAD4* is similar in CAC compared to sporadic, non-hypermutated CRC; (ii) the frequency of *APC* gene mutations was markedly lower in CAC, compared to sporadic, non-hypermutated CREC and the same applies for *KRAS*; (iii) a group of mutations involving the genes responsible for cell motility and cytoskeleton remodeling (*RAC1, DOCK2, DOCK3, PREX2* and *RADIL*) is predominantly mutated in CAC; (iv) *TP53* is similarly mutated in both CACs and non-hypermutated CRCs; and (v) some epigenetic regulators and chromatin modifiers, such as *EP300* and *TRRAP*, are preferentially mutated in CACs [161]. The lower frequency of APC mutations in CACs is surprising and deserves some comment; in spite of this lower frequency of APC mutations, other events contribute to the activation of the Wnt/β-catenin pathway in CACs, including the inactivation of mutations of SOX9 and of some genes associated with the activation of β-catenin (such as *TCFZL2, FZD8, AX1N1*), thus explaining the frequent (41%) nuclear β-catenin accumulation observed in CACs; these observations support the hypothesis that Wnt/β-catenin activation in CAC can ensue even in the absence of APC mutations. Finally, although CACs and CRCs have similar rates of *TP53* mutations, the identity and molecular distribution of these mutations is different in these two types of colorectal tumors [156].

Another recent study reported the extensive genomic characterization of 47 CACs [162]. This study in large part confirmed the findings of the other study, with the notable exception that in this study the frequency of *TP53* alterations was higher among CACs than in sporadic CRCs, and the frequency of KRAS mutations was similar in CACs and in sporadic CRCs [162]. In addition, this study provided evidence of some remarkable differences between CACs developed in CD and in UC patients: the frequency of both *APC* and *IDH1* mutations was higher among CD- than UC-derived colon cancers [161]. Finally, this study also showed that *c-Myc* and *IDH1* mutations were more frequent in CACs than in sporadic CRCs [162]. All these studies have contributed to postulating a model of inflammation-related colon cancer development. This model of inflammation-related colon cancerogenesis implies progression through various stages of tissutal alterations from mucosal inflammation (colitis) to indefinite dysplasia, low-grade dysplasia, high-grade dysplasia and, finally, carcinoma. In this model, some key genetic alterations are progressively acquired during disease progression: *p53* mutations and p53 LOH at early stages, *KRAS* mutations at the high-dysplasia stage and *APC* and *GSK3β* mutations at the carcinoma stage [163]. Following this model, *TP53* alterations (gene mutations and loss of heterozygosity at the *p53* locus) are an early event in the progression of CACs. This hypothesis is supported by various lines of evidence: (i) the loss of heterozygosity at *p53* correlates with malignant progression [164]; (ii) *p53* mutations are observed in colonic mucosa without dysplasia and in inflammed mucosa without cancer [157]; (iii) in bioptic specimens of CD and UC analyzed at various times during disease progression, p53 mutations were shown to occur early, before aneuploidy [164]; (iv) *p53* mutations precede the loss of heterozygosity of *TP53*, which increases with disease progression from samples without dysplasia to high-grade dysplasia and finally, to cancer; and (v) in experimental murine models mutant *p53* prolongs nuclear factor kappa-light of activated B cells (NF-kB) activation and promotes chronic inflammation and inflammation-associated colorectal cancer [165]. P53 mutations were also observed in CD and an association between these mutations and dysplasia and the progression of dysplasia was observed [166]. The role of Wnt/β-catenin pathway in inflammatory bowel disease (IBD)-associated colon cancer development is less clearly defined. One view suggests that this pathway is less involved in IBD-dependent carcinogenesis: this view is supported by the observation that APC loss is a less frequent event in IBD-associated colon cancer than in sporadic CRC and occurs late in the development of colitis-associated dysplasia and cancer [167]. Therefore, APC is unlikely to have a gatekeeper function in colitis, and alternative mechanisms drive the Wnt activation signaling that drives cell proliferation or, alternatively, continuous inflammation and reparation provide a proliferative drive sufficient to substitute for APC lesions. However, as shown by Robles et al. [161], several genetic abnormalities, not directly involving the *APC* gene, lead to Wnt/β-catenin activation in IBD-derived cancer cells.

Other mutations, such as *KRAS* mutations, are less frequent in inflammatory colon cancers and usually occur at later stages of disease progression [168]. However, in some cases KRAS mutations are observed in early colitis lesions, suggesting that they may in some instances initiate mutations [156,169]. Chromosomal instability develops early in UC and can be detected in histologically non-dysplastic tissue and seemed to precede the development of dysplasia in these patients [170,171]. A higher level of genomic instability was found in UC patients with dysplasia compared to patients with UC without dysplasia; genomic instability may be triggered by oxidative-mediated DNA damage [172]. High-microsatellite instability was observed in about 15% of sporadic CRCs not associated with IBD. Similar frequencies of MSI-high were reported in intestinal bowel disease-associated neoplasias (IBD-Ns), ranging from 9 to 15% [172,173,174]. Compared with sporadic MSI-H CRCs, IBD-associated colorectal cancers are characterized by some peculiarities, mainly represented by the heterogenous mismatch repair defects involving *MLH1, MSH2, MSH6,* or *PMS2*, and a low frequency of MLH1 promoter methylation [173,174]. In line with sporadic MSI-H CRCs, the IBD-associated cancers displayed frequent *BRAF* and *TGFBR2* mutations [173,174].

### 3.9. Molecular Abnormalities Associated with the Serrated Pathway

The progress of molecular studies on the characterization of the molecular abnormalities of colorectal cancers, together with histological studies, have shown that the serrated pathway is responsible for the development of about 30–35% of all colon cancers. Serrated polyps can arise in a sporadic or familial polyposis setting and predispose colorectal cancer development, particularly in those with MSI due to *MLH1* promoter methylation (*MLH1*^me+^), while germline DNA mismatch repair gene mutations cause MSI colorectal cancer without *MLH1* promoter methylation. Serrated lesions were classified into three different types by the World Health Organization (WHO): hyperplastic polyps (HP), sessile serrated adenoma/polyps (SSA/P) and traditional serrated adenoma (TSA). SSA/P with dysplasia are rarely encountered and represent an intermediate step to malignant progression, frequently associated with loss of MLH1 expression [175]. The SSA and TSA pathways are strongly associated with colorectal cancer development and recent studies have greatly contributed to clarifying the cellular and molecular pathways involved in the progression along these pathways. The earliest mutations observed in precursor TSA lesions are BRAF and KRAS mutations. There is evidence that these two genes are responsible for the evolution of two different TSA subtypes: (i) *BRAF* mutant TSAs are more frequently proximal, are associated with a precursor polyp (SSA) and are more frequently a CpG island. *CDKN2A* silencing is essential for the molecular progression of these TSAs; in spite of the presence of *BRAF* mutations, these TSAs display a microsatellite-stable phenotype and they originate very aggressive colon carcinomas; (ii) *KRAS* mutant TSAs are not associated with a precursor polyp and usually are not of the high CpG island methylator phenotype. *β-catenin* and *TP53* mutations are essential for the molecular progression of these TSAs, which display a microsatellite-stable phenotype [176,177]. In conclusion, there are two types of serrated polyps from which BRAF mutant cancers arise. The most common is sessile serrated adenoma, which occurs predominantly in the proximal colon. These lesions are characterized by abnormal crypt architecture, without cytological dysplasia. Serrated polyps have a BRAF mutation and a CIMP phenotype, but not MLH1 silencing and a MSI condition. Development of cytologic dysplasia in a sessile serrated adenoma is associated with a rapid progression to malignancy and concomitantly with this progression in neoplastic transformation, where the development of MSI and MLH1 methylation occur. It was recently estimated that 75% of sessile serrated adenomas with methylate MLH1 are MSI, and progress to mutant *BRAF* MSI cancers; however, about 25% do not silence the *MLH1* gene and become *BRAF* mutant MSS cancers [178]. The methylation of the *MLH1* gene seems to be related to the presence of a gene polymorphism at the level of the *MLH1* gene (MLH1-93 G/a polymorphism): the *MLH1*-93 AA genotype is significantly associated with promoter hypermethylation and MLH1 loss in the context of a sessile serrated adenoma [179]. The second type of serrated polyp with malignant potential is the traditional serrated adenoma (TSA), which is a relatively rare polyp occurring in the distal colon [176]. *BRAF* mutation is present in the majority (<60%) of these polyps and the majority display a CIMP phenotype [176]. Silencing of *MLH1* is extremely rare in TSA and thus these polyps are a source of MSS cancers [176].

Another early abnormality observed in TSAs is represented by Gremlin1 (*GREM1*) overexpression. GREM1 is a ligand sequestering BMP antagonist, overexpressed in hereditary mixed polyposis syndrome due to a gene duplication event. GREM1 levels are markedly increased in TSAs and not in other colon polyps [146]. Studies in mouse models have suggested that aberrant GREM1 expression disrupts intestinal morphogen gradients and promote the persistence of LGR5^−^ intestinal progenitor cells in foci distant from the crypt base, rendering these cells prone to tumor-causing somatic mutations [180]. According to these findings, it was proposed that *GREM1* cooperates with *BRAF/KRAS* mutations to drive the formation and proliferation of TSAs [79,180].

**Figure 6:**
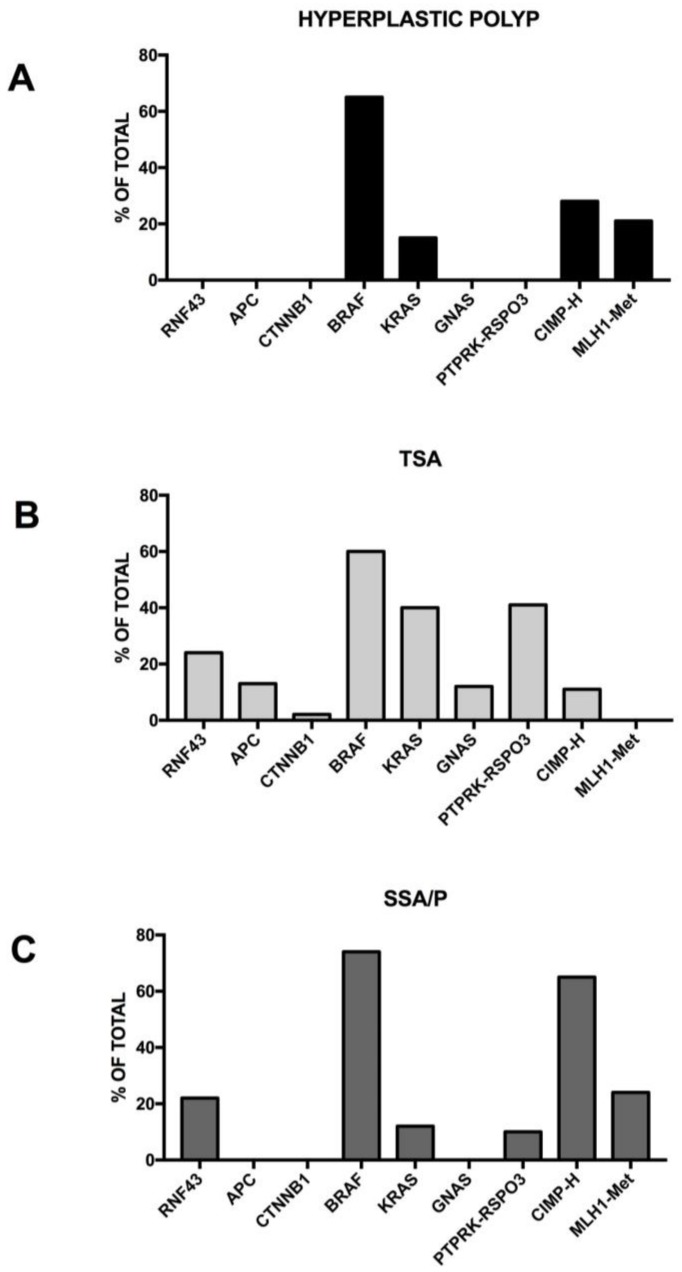
Frequent genetic alterations, CIMP-H and MLH1-methylated in hyperplastic polyps (**A**), traditional serrated adenomas (TSA) (**B**), and sessile serrated adenomas/polyps (SSA/P) (**C**). The data are reported by Sekine and coworkers [184].

Recent studies have shown that most TSAs contain genetic alterations in the WNT pathway, represented by *PTPRK-RSPO3* fusions, *RNF43* mutations and *APC* mutations. Recent studies have reported the recurrent inactivating mutations of the tumor suppressor gene *RNF43* (an E3 ubiquitin ligase that acts as a WNT inhibitor by targeting WNT receptors for degradation) occurring in about 18% of sporadic colorectal cancer patients [181]. Interestingly, the frequency of *RNF43* mutations is much higher in MSI (79%) than in MSS or MSL colorectal cancers, thus suggesting that mismatch repair deficiency induces the development of a permissive environment for the frequent *RNF43* frameshift alterations involving coding 659 and 117 [182]. More recent studies have analyzed the frequency of *RNF43* mutations among CRC patients with *BRAF* mutations associated or not with MSI. Thus, the frequency of *RNF43* mutations among BRAF mutant/MSI CRCs (87%) is markedly higher than in *BRAF* mutant/MSS CRCs (24%); RNF43 mutations are rare (4%) among *BRAF* wild-type/MSS CRCs [183]. Several studies have analyzed the occurrence of *RNF43* mutations in TSAs and SSAs: some studies reported a higher frequency of *RNF43* mutations in TSAs than in SSAs [184,185], while other studies reported the contrary (Figure 6) [186]. These discrepancies are seemingly related to the difficulties in the classification of these adenomas and to the property of some SSA lesions to evolve into TSA lesions. In MSI colorectal cancers, *RNF43* mutation is more frequent in the *MLH1^me+^* than *MLH1^me−^* group, whereas *APC/β-catenin* mutation is more frequent in the latter [186]. Both in the MSI and MSS colon carcinoma groups, there is a strong tendency for the co-occurrence of *RNF43* with *BRAF* mutations [185]. Hashimoto and coworkers compared the occurrence of WNT pathway gene mutations in sessile serrated adenomas with or without dysplasia: RNF43, APC and ZNF43 mutations were identified in 50%, 9% and 7% sessile serrated adenomas with dysplasia, but mutations of these genes were rarely observed in sessile serrated adenomas without dysplasia [187]. BRAF or KRAS mutations were observed in both non-dysplastic or dysplastic sessile serrated adenomas; most MLH1-deficient sessile serrated adenomas with dysplasia displayed RNF43 mutations less frequent in sessile serrated adenomas with retained MLH1 expression [187].

R-Spondin fusions, including protein tyrosine phosphatase receptor type K-R-Spondin (PTPRK-RSPO3) and eukaryotic transplation initiation factor subunit E/R-Spondin 2 (EIF3E-RSPO2) fusion, have been reported in 4–10 colorectal cancers [100,180]. PTPRK-RSPO3-positive CRCs were found to be positive for BRAF or KRAs mutations and were MSS [101,188]. PTPRK-RSPO3 fusions were recently reported in 31% of TSA and were absent in conventional-type adenomatous and SSA, as well as hyperplastic polyps [188]. Since PTPRK-RSPO3 fusions were selectively observed in TSAs, it was hypothesized that PTPRK-RSPO3 fusion-positive colorectal cancers are issued from TSAs [188]. In line with this hypothesis, PTPRK-RSPO3-positive colorectal cancers are positive for KRAS or BRAF mutations and are microsatellite-stable. R-Spondin, fusion-positive colon cancers display WNT pathway activation; R-Spondins are able to amplify WNT signaling and to drive tumor proliferation. In fact, targeting RSPO3 in PTPRK-RSPO3-fusion positive tumor xenografts inhibits tumor growth and promotes tumor cell differentiation [189]. In these cells RSPO3 blocking exerts an inhibitory effect on the expression of genes expressed in the stem cell compartment [189]. According to these findings, it was suggested that a stem cell compartment drives PTPRK-RSPO3-positive tumor cell growth and targets RSPO3, inhibiting this tumor stem cell compartment [189]. Interestingly, the study of serrated polyps (SSA/Ps) allowed the definition of a unique gene signature: this signature identifies 1422 differentially-expressed genes relative to controls [169]. Serrated polyposis syndrome and sporadic SSA/Ps exhibited an almost complete gene overlap (about 96%). This gene signature allowed the identification within colon cancers of a subtype of colon cancers; furthermore, a 51-gene panel in SSA/P showed a similar expression in a subset of colon cancers with high MSI [189]. Finally, a seven-gene panel showed high sensitivity and specificity in identifying BRAF-mutant, CIMP-high, and MLH1-silenced colon cancers [190].

Recent studies suggest that the loss of expression of the homeobox protein CDX2 could represent an important molecular abnormality observed in colorectal cancer of the serrated type. CDX2 is a master regulator of intestinal development and oncogenesis and its expression is highly specific to the intestinal epithelium. Thus, Kim and coworkers have explored a large group of MSI-H colorectal cancers for CDX2 protein levels and observed that about 14% of these tumors exhibited CDX2 loss of expression [191]. CDX2 loss in these tumors was correlated with lymph node metastasis, poor differentiation, MLH1 loss, BRAF mutations, and CIMP-H status [190]. Landau and coworkers provided evidence that CDX2 loss was observed not only in MSI-H BRAF-mutated colorectal cancers, but also in a subgroup of MSS BRAF-mutated colorectal cancers, characterized by an aggressive clinical phenotype and increased Cytokeratin 7 expression expression [86]. Bae and coworkers screened 713 colorectal cancers for CDX2 expression using immunohistochemistry (IHC) and discovered that about 6% of these tumors exhibited CDX2 loss: at the histological level, CDX2 loss was associated with poor differentiation, an increased number of tumor-infiltrating lymphocytes, luminal serration and mucin production, and at the molecular level, CDX2 loss was associated with CIMP-H, MSI-H and BRAF mutations [87]. CDX2 loss was associated with a negative prognosis, according to both the univariate and multivariate analysis [87].

### 3.10. Molecular Abnormalities in Synchronous Colorectal Cancer

Synchronous colorectal carcinoma (sCRC) occurs in 2–5% of newly diagnosed CRC patients and is defined as more than one primary colorectal cancer detected in a single patient at the time of diagnosis. Hereditary CRC syndromes and inflammatory bowel diseases have an increased risk of developing sCRCs. Recent studies have analyzed the molecular defects of sCRCs. Some studies have suggested that sCRCs are frequently associated with the CIMP phenotype [192] and with an increased prevalence of BRAF mutations [193]. Furthermore, the CIMP phenotype correlated between synchronous cancer pairs from the same patients [164]. Furthermore, sCRC patients seem to have a higher proportion of MSI tumors than patients with single tumors [192]. However, Malesci and coworkers reported that MSS cancers presenting with the phenotype of multiple advanced lesions account for the vast majority of sCRCs and do not have BRAF mutations [194]. A recent study based on the analysis of a relatively large group of sCRC tumors confirmed that the majority of these tumors are MSS [195]. Interestingly, a discordant mutation status was observed for KRAS and TP53 in various tumor foci in single patients with sCRCs [196,197,198]. Recent studies have analyzed in more detail the genetic heterogeneity of sCRCs, providing evidence of the existence of a strong inter-tumoral and intra-tumoral genetic heterogeneity [199,200]. Thus, 48% of the sCRCs displayed double and even triple mutations in KRAS, APC, TP53, PIK3CA and TGFBR2 [199]. This inter- and intratumor heterogeneity must be carefully considered in the selection of therapy and in the monitoring of the resistance to therapy [199,200]. Interestingly, sCRC patients displayed a higher occurrence of germline inherited damaging mutations in immune-related genes compared to patients with solitary CRC [200]. According to these findings it was suggested that inherited damaging alterations of immune-related genes may increase the frequency of independent cancer-initiating events [200].

### 3.11. Molecular Abnormalities Associated with Metastatic Disease

**Figure 7:**
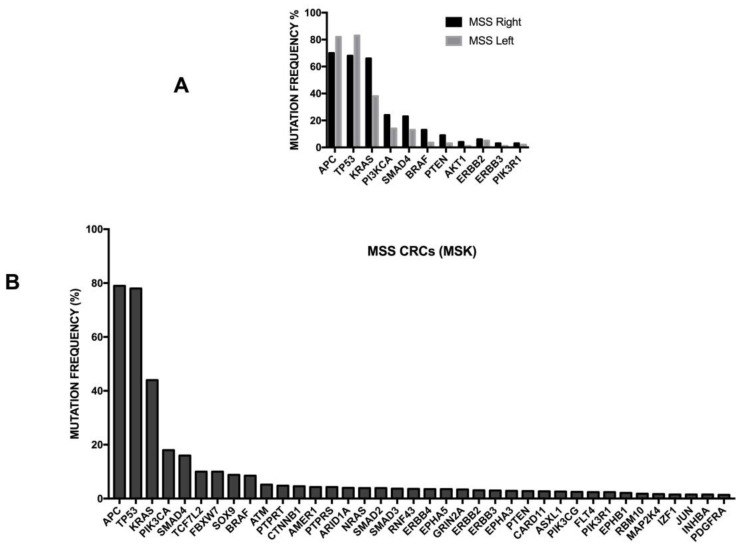
Recurrently altered genes in advanced colorectal patients. (**A**) Frequency of mutations of the whole population of MSS colorectal cancers. (**B**) Frequency of mutations in MSS colorectal cancer subdivided into left and right according to tumor location. The figure shows the data reported by Yaeger et al. [201].

At the end of this chapter analyzing the studies of the molecular abnormalities of colorectal cancers it is interesting to report the results of a recent study reporting the molecular analysis of a large set of colorectal cancers, mostly derived from patients with advanced metastatic disease [201]. This analysis allowed the evaluation of the genomic landscape—including mutational, copy number and rearrangement analysis—of more than 1000 colorectal cancers. This new, large-scale analysis allowed a better definition of the genetic alterations occurring during the metastatic stage of colorectal cancer. The cases of colorectal cancer analyzed were classified into three different molecular groups: POLE mutant (0.7%); MSI-H/hypermutated (8.7%) and MSS (90.5%). MSS tumors were mostly localized at the level of left colon, while MSI-H and POLE mutant tumors were predominantly observed in the right colon. At the histological level, MSS tumors were predominantly of the conventional type and are moderately differentiated, while MSI-H and POLE mutant tumors had a predominantly mucinous histology and were frequently scarcely differentiated [201]. The analysis of mutated genes in MSS colorectal cancers showed the occurrence of 47 recurrently mutated genes, the most frequently mutated being *APC* (79%), *TP53* (78%), *KRAS* (44%), *PIK3CA* (18%), *SMAD4* (16%), *TCF7L2* (10%) and *FBXW7* (10%) (Figure 7) [201]. This analysis also identified new recurrently mutated genes—including *PTPRS, PIK3GG, FLT4, MAP2K4, IK2F1, JUN, TBX3, FOXP1, INHBA* and *CDKN1B*—affecting from 1 to 4% of tumors [201]. The analysis of the frequencies of gene mutations in early-stage tumors, primary tumors or metastases from metastatic colon cancers showed that some of these mutations are stage-related: FBXW7 mutations are more frequent in early-stage and primary metastatic tumors than in metastases; ERBB2 mutations are more frequent in early-stage tumors than in metastases; and the frequency of TP53 mutations progressively and moderately increases from early-stage tumors to metastases [201]. The analysis of WNT pathway alterations showed that, in addition to APC, other recurrently mutated genes in the WNT pathway were CTNNB1 and RNF43, thus providing evidence that the overall WNT pathway alteration in MSI-H tumors is 93% and in MSS tumors is 85% [201]. In MSI-H tumors, CTNNB1 mutations activate mutation at the level of hot-spot regions, while in MSS tumors, the most frequent CTNNB1 mutation is represented by a cluster of intragenic deletions at the level of exon 3 [201]. Interestingly, the authors of this study performed a careful analysis of the actionability of the genomic alterations that are enriched in MSI-H/hypermutated tumors, compared with MSS tumors (86% vs. 37%), including BRAF^V600E^ (22% vs. 5%), BRCA 1–2 alterations (22% vs. 1%), and NTRK fusions (8% vs. 1%). PI3KCA and PTEN mutations were significantly higher in MSI-H than in MSS CRCs (41% vs. 16% and 35% vs. 5%); and 46% of right-sided and 30% of left-sided MSS tumors have potentially targetable oncogenic alterations, consisting primarily of BRAF^V600E^ and PIK3CA mutations [201]. There was a significant enrichment of oncogenic alterations in KRAS, PIK3CA, BRAF, PTEN, AKT1, RNF43, SMAD2 and SMAD4 in right-sided primary tumors and of APC and TP53 in left-sided primary tumors (Figure 7) [201].

### 3.12. Intratumor Heterogeneity and Colorectal Cancer Evolution

Recent studies have addressed the important problem of intra-tumor heterogeneity, a condition that often represents a condition greatly limiting the efficacy of current therapies. The intra-tumor heterogeneity may be observed at the level of the various areas of the primary tumor or at the level of multiple metastases or between the primary tumor and metastases. Thus, a set of studies have explored the mutational concordance of colorectal cancer tissue lesions through paired analysis of surgical specimens of primary and metastatic lesions. An initial fundamental study by Jones et al. [39] compared the mutational spectrum of metastases with primary colon cancers, providing evidence that only 3% of the mutations were not found in the colon cancers from which the metastases arise [39]. Furthermore, the mutational spectrum of different metastatic lesions present in a single CRC patient was comparable (only about 2.3% of mutations are metastasis-specific). Finally, in rare CRC patients in whom the original adenoma lesion was not destroyed by carcinoma development, it was possible to demonstrate a 30% mutational difference between the adenoma and carcinoma, mostly involving cancer-related genes [39]. Additional studies showed that the major pathway genes, including KRAS, TP53, APC, PIK3CA, BRAF and NRAS, are often concordant between the primary and metastatic lesions, regardless of the temporal relationship of metastases (synchronous or metachronous) [202,203]. Similarly, Jesinghaus and coworkers reported 100% genetic concordance between primary and multiple secondary sites for APC, KRAS, FBXW7, PIK3CA, BRAF, SMAD4 and ACVR2A; except for the true de novo mutations occurring in 16% of cases (involving TP53, CTNNB1, PTEN and SYNE1), all remaining cases (86%) shared the genetic lesions of the primary tumors with metastases, irrespective of the site of metastasis or the time lapse between primary tumor and the development of metastatic spread [204]. Jeantet et al. reported an analysis of a larger number of CRC patients and showed that intra-tumoral heterogeneity for RAS mutation in the primary tumors was found in 33% of cases, while inter-tumoral heterogeneity for RAS mutation between primary tumors and metastatic lymph nodes or distant metastasis was found in 36% of cases; finally, 28% of tumors had multiple RAS mutated subclones in the same tumor [205].

Kim and coworkers performed a detailed analysis based on multiregion biopsies in both primary and metastatic colorectal cancer lesions from five CRC patients, and analyzed the mutational spectrum and CNAs [206]. Various types of mutations were described in this study and classified according to their spatial localization: universal mutations are those present in all tumor lesions; metastasis-clonal mutations are those with a partial presence or absence in the primary lesions; primary-private mutations are observed in specific primary biopsies; and metastasis-private mutations are observed in specific metastasis biopsies [206]. The number of mutations between the primary and metastatic biopsies of each patient was not significantly different. From 20% to 54% of mutations in a given sample were universal; among the subclonal lesions, from 1 to 15% were metastasis-clonal, from 2% to 41% metastasis-private and from 14 to 56 primary-private [206]. APC, KRAS and TP53 mutations were identified as universal events [144]. Most CNAs were observed in both primary and metastatic lesions, thus representing early or universal genomic events [206]. Finally, the inferred evolution pattern of cancer progression was branched evolution, rather than linear evolution [206].

Sveen et al. analyzed the intra-patient, inter-metastatic genetic heterogeneity in CRC patients through the evaluation of copy number alterations. The level of heterogeneity of this parameter was highly variable in the different patients; interestingly, intra-patient, inter-metastatic heterogeneity was a strong prognostic determinant, in that patients with a low level of heterogeneity had a three-year survival rate of 66%, compared to 18% for patients with a high level of heterogeneity [207].

Uchi and coworkers profiled the genome and epigenome in multiple regions within each of nine CRCs [206]. Extensive intertumor heterogeneity was observed, from which the evolutionary history of tumors was inferred. First, clonally shared alterations appeared, involving mutations of driver genes (*APC*, *KRAS*), then C > T transitions at CpG sites and finally CpG island hypermethylation. The existence of a correlation between mutation counts and patients’ ages strongly suggests that the early-acquired alterations are related to aging [208]. At later phases, a parental clone is branched into numerous subclones; the branching evolution is generated by neutral evolution. The analysis of individual cases of tumor evolution was particularly interesting. Thus, in a single case it was observed that in the founder phase, the parental clone accumulated mutations in driver and founder genes (*APC*, *KRAS* and *FBWX7*), together with copy number loss or gain and hypermethylation and the hypomethylation of selected gene promoters. In the progressor phase the founder clone divided into two subclones: one harboring MYC amplification and the other with several copy number alterations. Subsequently, the two subclones branched into minor subclones, progressively accumulating progressor alterations; finally, the liver metastasis lesion originated from a polyploid-like subclone [207]. The study of the tumor heterogeneity of CRCs was of fundamental importance for a better understanding of the molecular and cellular mechanisms responsible for the development of this cancer and greatly contributed to proposing new theories to explain CRC development. Particularly, Sottoriva and coworkers proposed a “Big Bang” model of human colorectal tumor growth, hypothesizing that after the initial transformation event, colorectal tumors grow as a clonal expansion populated by numerous intermixed subclones [209]. The Big Bang model is supported by four types of observations: (i) intratumor heterogeneity is an intrinsic property of colorectal cancers, occurring early during tumor development and progressively increasing in tumor progression, and is not influenced significantly by clonal selection; branched phylogenies are the logical consequence of this initial heterogeneity; (ii) Clonal expansions or selective seeps are rare events during the progression of CRCs from an initial stage to an advanced stage of tumor development; (iii) Most of the public and private genetic alterations are early events and become pervasive during tumor development, thus dominating the genomic structure of the advanced neoplasia; (iv) Aggressive subclones may remain rare in the primary tumor but become dominant and fuel resistant under the selective pressure induced by some anti-tumor treatments [209].

Several findings directly support this theory. In fact, Kang et al. showed that colorectal adenomas display a high degree of intratumoral heterogeneity present between tumor sides and individual glands. Interestingly, private mutations are side-specific and subdivide the adenomas into two major subclones [210]. Thus, these observations support the suggestion that intra-tumor heterogeneity originates early during the history of colorectal cancer. Sievers has investigated the mutational spectrum of small colorectal polyps and has shown that: small polyps can have multiple driver pathogenic mutations—67% APC mutations, 15% KRAS mutations, 8% TP53 mutations, 10% FBXW7 10% mutations and 17% BRAF mutations (SSAs and HPs)—small polyps can have multiple driver mutations—31% of these tumors display multiple pathogenic mutations—adenomas contain subpopulations at lower allelic frequencies—private mutations—and statistical inferences predict that detectable intratumoral heterogeneity is an event occurring early during adenoma development [211]. Additional evidence in favor of the “Big Bang” model of tumor growth comes from additional recent studies. Thus, Williams et al. analyzed the subclonal mutant allele frequencies of various cancers and reached the conclusion that those of colorectal cancer, as well as of other cancers (such as stomach, bladder, lung and cervical cancers) follow a simple power-law distribution predicted by neutral growth [212]. In these malignancies, all clonal selection events seemingly occur before the onset of cancer growth and a lot in late-occurring subclones, thus resulting in the numerous passenger mutations that are responsible for tumor heterogeneity [212].

The role of passenger mutations was recently explored providing evidence that these mutations, although individually weak, together in their collective burden are able to alter tumor progression [213,214]. In contrast to a common view, many passenger mutations have a damaging effect and have the capacity to evade negative selection [213,214].

Lineage tracing experiments in human colorectal adenomas using a combination of nuclear and mitochondrial DNA lesions and epigenetic markers further support the “Bing Bang” theory of colony cancer development. These observations led to the identification of a stem cell population within human adenomas and suggests that the growth of new clones (no clonal sweeps) occurs rarely, certainly not as a steady and continual process, as it is commonly assumed [215]. According to these observations, a colon cancer development model was proposed, implying that clones formed at the onset of neoplasia formation do not sweep through the tumor and appear as localized clones, with divergent intraclone methylation patterns; rare subclones form later during tumor development [215].

In conclusion, the patterns of genetic heterogeneity observed within colorectal tumors are compatible with a “Big Bang” expansion of tumor cells that grow as a single cell expansion, characterized by numerous early-arising clones, and coexisting within the tumor for long periods of time due to the absence of selective pressure; this weak selection operating within the tumor was incapable of large clonal expansion in a short time. This implies that the heterogeneity observed within the developed tumor is in large part already generated at the level of the early tumor, well before its clinically detectable development [216].

As stated above, the “Big Bang” model, which is based on the concept of punctuated evolution, implies that the large majority of genetic alterations are acquired during the early stages of carcinogenesis; however, this model implies that the accumulation of this mutational load could only happen after truncal alterations in genes essential to colorectal cancerogenesis have occurred in a founder clone, thus in line with a monoclonal, monocryptal origin of colorectal cancer [216]. However, there is growing evidence that intestinal tumors are often polyclonal rather monoclonal. This evidence is not limited to mice, but it extends also to human colorectal cancers, both hereditary and sporadic [217,218]. These observations challenge the monocryptal, monoclonal origin proposed by the “Big Bang” model. According to polyclonality, multiple initiating clones originate from different distinct dysplastic crypts, from which one will emerge as dominant and will drive colorectal carcinogenesis. Recent studies supported the multiclonality of adenoma lesions. Thus, Borras and coworkers performed wide exome sequencing in 37 adenomas [219]. Their results showed that the presence of recurrent alterations in known cancer driver genes, such as *APC, KRAS, FBXW7* and *TCF7L2* and abnormalities in chromosomes 5, 7 and 13; 80% of adenomas displayed somatic alterations in WNT pathways; the adenomas displayed evidence of multiclonality similar to stage I carcinomas [219]. Interestingly, in this study, it was also observed that the at-risk colon mucosa in part displayed the genetic alterations observed in adenomas, thus indicating the evolution from normal tissue to premalignant adenoma and surrounding mucosa and then to carcinoma [218]. Gausachs et al., in a recent study, provided evidence that the mutational heterogeneity in *APC* and *KRAS* observed in adenomas arises at the crypt level [220]. This conclusion was based on the ultrasensitive genotyping of matched bulk biopsies and individual crypts of colorectal adenomas and carcinomas: this analysis observed the presence of novel *APC* mutations, abundant wild-type *APC* crypts coexisting with mutant crypts, and mutational heterogeneity in *KRAS* within crypts, events not detectable through the analysis of standard biopsies [220]. The nonrandom heterogeneity among the crypts supports a polyclonal model as the origin of multiclonality in colorectal cancer. The fact that intratumor heterogeneity arises at a premalignant stage from the convergence and admixtures of multiple crypts harboring different *APC* and *KRAS* mutations provides fundamental data on the mechanism of the initial evolution of colorectal cancers [220]. These findings imply a role for *KRAS* in the early stage of colon cancerogenesis and a cooperation with *APC* mutations. Several data support a possible early role of *KRAS* in colorectal cancerogenesis and a cooperation with *APC* mutations: in fact, KRAS activates the translocation of β-catenin to the nucleus [39], thus inducing cancer stemness [221] and increasing the intestinal crypt fissions between neighboring colorectal crypts [222].

Several recent studies have better explored the mutational spectrum of early adenoma lesions. Thus, Lin and coworkers performed a large-scale sequencing of 149 adenoma samples and identified several potential driver genes preferentially mutated in adenomas, compared to colorectal cancers. Colon adenomas and serrated adenomas have the same mutation frequencies, but the genes involved differed substantially, the most mutated genes in conventional adenomas being APC and the most mutated genes in serrated adenomas being *BRAF* [223]. The wide exome sequencing and target sequencing showed that: *TP53, PIK3CA, KRAS, APC* and *SMAD4* had a trend in mutation prevalence towards colorectal cancer; in contrast, both novel and known colorectal cancer-related mutations with driver patterns have been observed in adenomas. APC is the gene most frequently mutated in conventional colon adenomas; four genes recurrently mutated in adenomas *CTNNB1* (catenin-β1), *KRTAP4-5* (keratin-associated protein 4–5), *GOLGA8B* (golgin A8 family member B) and *TMPRSS13* (transmembrane protease, serine 13) had the oncogene pattern [222]. Lee and coworkers analyzed by whole exome, sequencing 12 high-grade colon adenomas (HGCAs), premalignant lesions that closely precede colon carcinoma. A total of 22 tumor-related genes were found to be mutated in these adenomas, the most frequently mutated being *APC* (10/12), *KRAS* (7/12), *SMAD4* (3/12), *AERBB4* (2/12), *TCF7L2* (2/12), *AMER1* (2/12), *TP53* (2/12) [224].

Interestingly, Shin and coworkers developed a model of colon cancer evolution based on the genome-wide analysis of all the somatic mutations in a large-scale molecular interaction network [225]. This analysis showed that a giant cluster, during colorectal tumor development, of mutation-propagating models undergoes a percolation transitions, corresponding to a sudden change from scattered small modules to a large connected cluster, which is accompanied by the typical phenotypic changes associated with cancer development [225].

Epigenetic changes occurring in various areas of colorectal cancers further enhance the level of tumor heterogeneity. This conclusion was reached in a recent study carried out by Martinez-Cardus and coworkers [226]. In this study, the authors analyzed the DNA methylation pattern in three different tumor areas: the invasive front—containing actively invasive cells giving rise to metastatic lesion—the central bulk and the internal surface [226]. Their findings showed that intertumoral epigenetic heterogeneity, related to differences occurring in the different patients, is higher than intratumoral diversity; the invasive front of the tumor is the most epigenetically divergent tumor region; surprisingly, liver metastases resemble more intratumoral sections than the invasive front (suggesting that invasion and micrometastatic disease occur at an early stage); the level of epigenetic heterogeneity had an impact on the outcomes of colorectal cancer patients, in that patients displaying a homogeneous epigenetic pattern have a poor prognosis compared to those displaying intratumor epigenetic heterogeneity [226].

Importantly, clinical studies have shown that the intratumor heterogeneity of colorectal cancers had a direct impact on therapy efficacy. In one study, tumor biopsies derived from patients participating in a trial based on cetuximab plus folinic acid fluorouracile ininotecan (FOLFIRI) in patients with *KRAS*-WT were analyzed by next generation sequencing. The results of this study showed that *KRAS* and *NRAS* mutataions are usually present in the large majority of tumor cells, whereas *BRAF* and *PIK3CA* mutations often affect a limited, subclonal fraction of tumor cells [227]. Surprisingly, in this study no direct relationship was found between the proportion of cells with KRAS mutations and cetuximab efficacy [227]. In contrast, in the CRYSTAL study, a correspondence was reported between the fraction of *KRAS* mutated tumor cells and cetuximab efficacy, with a benefit of cetuximab combined chemotherapy in patients with a low prevalence of *KRAS* mutations (0.1% to 0.5%) [228,229]. Other studies have confirmed the consistent heterogeneity of colorectal cancers at the level of primary tumors and synchronous metastases: this heterogeneity was present before and after anti-EGFR therapy [229]. Particularly, mutations were observed during therapy loss and the acquisition of *KRAS*, *NRAS*, *TP53*, *PIK3CA*, *FBXW7* and *PTEN* [230].

The existence of intratumor heterogeneity represents an element of considerable complexity, also for pre-clinical studies aiming to define the drug sensitivity of primary tumor cells. In fact, a recent study analyzed multiple spatially distinct biopsies from colorectal cancers and showed that basically tumor heterogeneity was recapitulated in matching patient-derived spheroids [231]. Thus, multiple cultures from spatially distinct sites of the tumor are necessary to obtain clinically relevant information and a representation of the genetic tumor subclones and of their biological complexity [231].

### 3.13. Tumor Location

Recent studies have shown that tumor location may represent an additional important source of heterogeneity in the biology and in the clinical phenotype of colorectal cancers. From these studies, remarkable differences emerge between tumors localized in the right colon, compared to those localized in the left colon and rectum. The right colon has a different embryological origin and blood supply from the left colon and rectum. Thus, the superior mesenteric artery supplies midgut structures from the mid-duodenum to the mid-transverse colon, while the inferior mesenteric artery supplies hindgut structures from the mid transverse colon to the rectum. The distribution of the different consensus molecular subtypes (CMS) of colon cancer in various anatomic regions suggests the existence of important biological differences in right-sided colon tumors. In fact, the large majority (>75%) of CMS1 tumors pertain to right-side tumors, while rectum cancers are rare (<5%); in contrast, the large majority of CMS4 tumors pertain to left-sided and rectum cancers (>90%) [232].

A recent review analysis of the biological and clinical properties of colon cancer patients subdivided by tumor site indicated that right-sided colon cancers and left-sided colon cancers differ in their microbiome, clinical characteristics, molecular profiling, clinical outcome and response to treatment; the reasons for these differences, at the moment, remain unclear [233].

Various studies have provided evidence of remarkable molecular diversities between right-sided and left-sided colon cancer. Thus, an initial study by Yamauchi et al. provided evidence that the frequency of CIMP-high, MSI-high and *BRAF* mutations gradually increased from rectum to ascending colon; in contrast, the frequency of *KRAS* mutations remained relatively stable at various tumor sites [97,234]. Sincrope et al. confirmed a higher frequency of *BRAF*-mutated tumors among proximal colon cancers and also showed that mutation in *KRAS* codon 12 was more frequent in normal tumors, while non-mutated *BRAF/KRAS* was increased in distal tumors [235]. The study by Lan and coworkers showed a higher frequency of *MSI, KRAS* and *PI3KCA* mutations in proximal than in distal colorectal cancers [236]. Salem and coworkers profiled the genomic alterations of a large set of colon cancers subdivided according to their tumor location [237]. They showed that right-sided colon cancers had higher rates of MSI, more frequent aberrant activation of EGFR pathways, more frequent *BRAF* and *PI3KCA* mutations and an increased mutational burden compared to left-sided and rectal cancers [237]. On the contrary, TP53 and APC mutations were more frequent in left-side/rectum cancers than in right-sided colon cancers [200]. On the contrary, *TP53* and *APC* mutations were more frequent in left-side/rectum cancers than in right-sided colon cancers [237].

Several studies have explored a possible impact of tumor location on response to therapy. These studies suggest that primary tumor location is a prognostic factor for patients with colorectal cancer. A meta-analysis of prospective and retrospective clinical studies reporting progression-free survival and overall survival data for right-sided and left-sided colon cancers reached the conclusion that right-sided colon cancers have a poorer prognosis than those with left-sided colon cancers [238]. A more recent meta-analysis of six trials comparing the therapeutic effect of chemotherapy plus EGFR antibody therapy, compared to chemotherapy alone, showed a significant benefit for chemotherapy plus EGFR therapy in patients with left-sided tumors, compared to no significant benefit for those with right-sided tumors [239]. In RAS-wild-type colon cancer treated with cetuximab as a salvage treatment, right-colon primary was associated with poorer survival outcomes that left colon and rectal cancer [240]. Taieb and coworkers performed a phase III randomized trial to determine the prognostic and predictive value of primary tumor location according to *BRAF, RAS* and MSI status in patients with stage III colon cancer receiving adjuvant treatment with FOLFOX, with or without cetuximab, in stage III colorectal patients categorized according to primary tumor site (proximal or distal). They found better disease-free survival in right-sided versus left-sided tumors in patients with *RAS* mutations and worse disease-free survival in right-sided versus left-sided tumors in patients with *RAS* and *BRAF* double wild type. These results were found independently of the treatment received, and no beneficial effect of cetuximab on disease-free or overall survival was observed in left-sided tumors [241].

**Figure 8:**
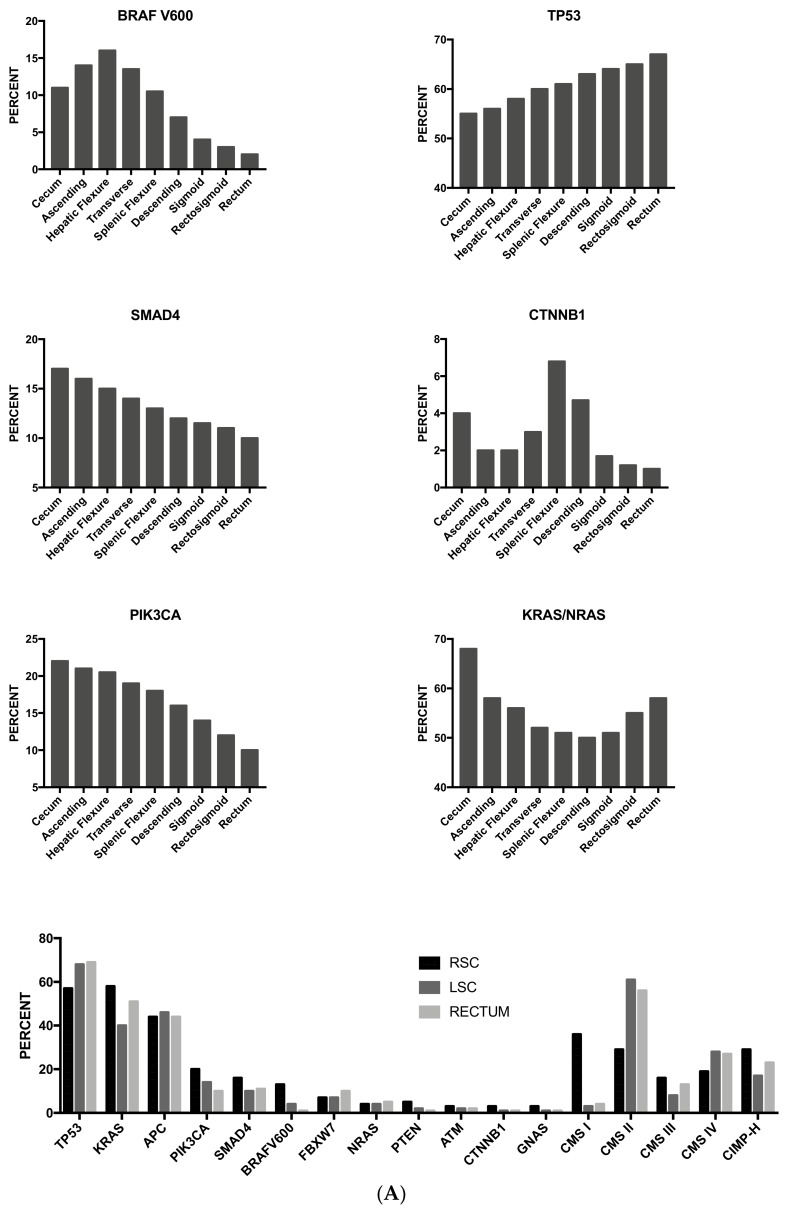

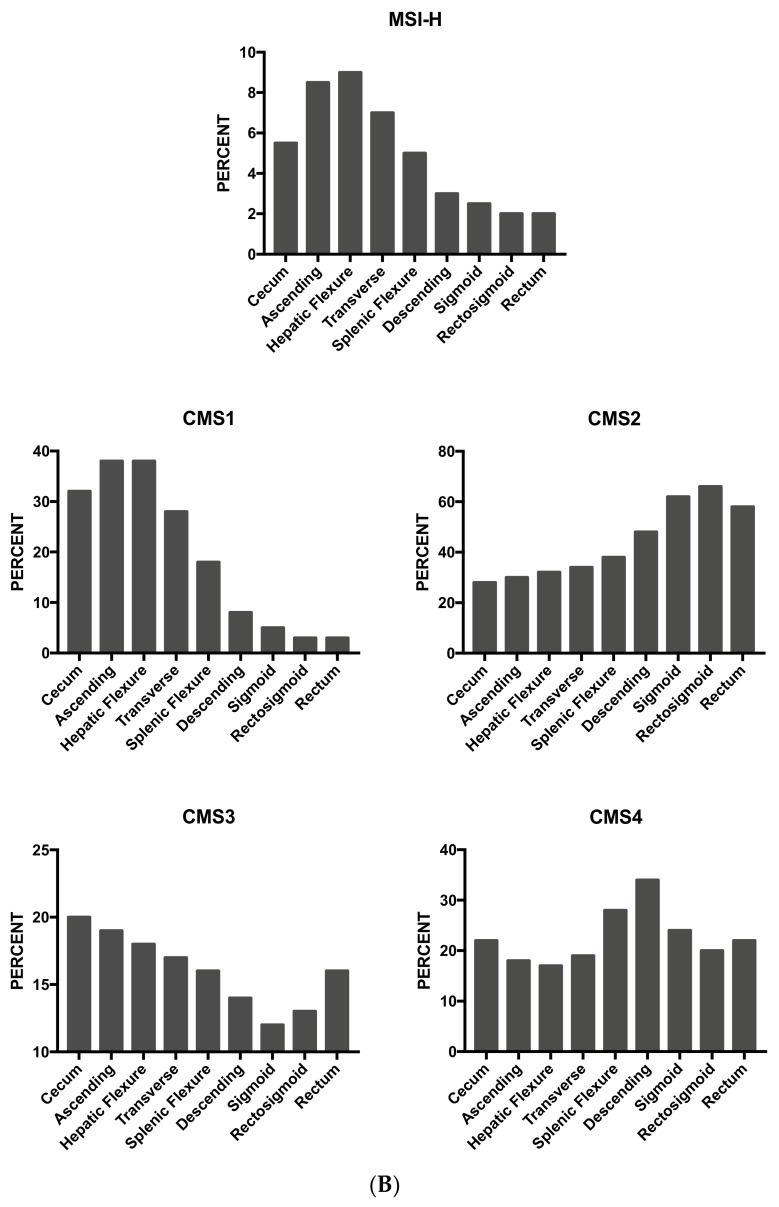
(**A**) Relative prevalence of key oncogenic alterations at specific primary tumor locations in patients with metastatic colon cancer. Panels 1 to 3 from the top to the bottom: frequency of BRAFV600, TP53, SMAD4, CTNNB1, PIK3CA and KRAS/NRAS alterations in primary tumor locations. Bottom pane: frequency of the main oncogenic alterations, consensus molecular subtypes and CIMP-H at the level of right-side colorectal cancer (RSC), left-side colorectal cancer (LSC) and rectum-located colorectal cancers. The data are reported by Loree et al. [242]; (**B**) Relative prevalence of consensus molecular subtypes (CMS) at specific tumor locations. The data are reported by Loree and coworkers [242].

Colorectal cancers are classified as right/left-sided based on whether they occur before or after the splenic flexure. However, a recent study showed that this subdivision is not optimal, and a more precise tumor location seems to be required to obtain more precise information [242] (Figure 8). This study showed that within right-sided tumors, *RAS* mutations decreased from 70% for cecal, to 43% for hepatic flexure location, while *BRAFV600* mutations exhibited the opposite pattern, increasing from 10% to 22% between the same locations [242] (Figure 8). At the level of the left-sided tumors, the sigmoid and rectal region displayed more *TP53* mutations, less *PI3KCA, BRAF* or *CTNNB1* mutations, and less MSI than tumors located on the other side [242]. Importantly, a left/right division preceding the transverse colon maximized the prognostic differences by side of tumor location [242].

In conclusion, although the current clinical treatment of colorectal cancer does not take into account the primary tumor site, left-sided and right-sided colon cancers harbor different biologic and clinical characteristics. Right-sided colon cancers are more likely to have genome-wide hypermethylation of the CIMP, hypermutated state via MSI-H, and *BRAF* mutations. Colorectal cancer subtypes are differentially distributed between right- and left-sided colorectal cancers, with a greater frequency of MSI/immune CMS1 and “metabolic” CMS3 subtypes observed in r ight-sided colon cancers. Growing evidence indicates that stage III-IV right-sided colorectal cancers have an inferior prognosis compared to the corresponding tumors located on left-side and that anti-EGFR antibody therapy is associated with better survival on left-side, stage IV colorectal cancers, compared with right-side colorectal cancers.

## 4. Gene Expression Studies

The current colorectal cancer staging is based on simple clinical-pathological features, mainly represented by bowel wall penetration, lymph node metastasis and distant metastasis. Duke’s classification is based on these criteria, and involves four stages of clinical cancer: stage A, where the tumor is localized in the innermost lining of the colon or only slightly growing into the muscle layer; stage B implies that the cancer has grown through the muscle layer of the colon; stage C implies that the cancer has spread to at least one lymph node in the area close to the bowel; stage D implies the spread of the tumor at the level of distant metastatic sites (liver, lung). Although Duke’s staging works very well for the good and poor prognosis groups (Duke’s stage A and D, respectively), it is not very informative in terms of predicting the prognosis of intermediate groups B and C.

Recent studies have shown that molecular staging, through gene expression profiling studies, was able to stratify the prognosis of Duke’s B and C patients and to identify those that can benefit from chemotherapy. According to gene profiling studies, Duke’s B and C patients are identifiec as those that can benefit from chemotherapy. According to gene profiling studies, Duke’s stage B and C patients can be subdivided into good and poor prognosis groups; this molecular classification improves the prognostic stratification of these patients and helps to identify patients who might be candidates for adjuvant therapy [243].

The other current system for the prognostic evaluation of colon cancer is the tumor nodes metastasis (TNM) staging system of the Americal Joint Committee on Cancer. According to this classification, patients with stage I have a five-year survival rate of approximately 93%, decreasing to approximately 80% for patients with stage II disease and 60% for stage III disease. However, this staging system did not allow the definition of stage II patients who could benefit from adjuvant chemotherapy. According to the analysis of the expression profile of 18 genes selected for their predictive potential, the colorectal cancer patients were subdivided into two subgroups, namely low risk (60%) and high risk (40%) patients; two thirds of the stage II patients were classified as low risk and one third as high risk [244]. This subclassification was important because it may help to identify patient subsets who are candidates for adjuvant chemotherapy [244].

Some gene expression studies have also attempted to address the problem of trying to differentiate colon and rectum cancers at the molecular level. Molecular and epidemiological studies have shown only minor differences between these two types of tumors. Some studies have suggested that *KRAS* and *BRAF* mutations are more frequent among colon than rectum tumors, while the chromosomal instability network is more common in rectal than in colon cancer. However, a recent study based on the analysis of a large number of colorectal cancer samples failed to show major differences between proximal colon, distal colon and rectum cancers; the only significant differences were observed at the level of some *Homeobox* (*HOX)* genes, but the most significant differences were observed between proximal and distal tumors, and not for colon and rectum tumors [245].

Gene expression studies may also help to identify patients responsive to specific treatments. Some colon cancer patients with metastatic disease have shown clinical benefits from treatment with EGFR antibodies: only 20–30% of these patients respond to EGFR inhibitors. At the molecular level, the resistance to EGFR inhibitors was associated with molecular alterations that constitutively activated EGFR signal transduction (*KRAS* mutations, *BRAF*, *PI3KCA* and *NRAS* mutations). A recent gene expression study provided evidence that it is possible to identify two different gene signatures, one associated with resistance and the other with sensitivity to treatment with anti-EGFR antibodies [246]. The gene signature was based on a combined signature involving activation mutation signatures for *KRAS*, *BRAF* and *PI3KCA*: according to this signature, the tumors were classified as “activated oncogenic” or “wild-type like” [246]. The former was associated with resistance and the latter with sensitivity to treatment with anti-EGFR antibodies [246].

Recent studies provided consistent progress in the definition of molecular subtypes of colorectal cancers. Perez-Villamil and coworkers using a gene expression profiling study based on the screening of 1722 selected genes, defined five colorectal cancer subtypes: cluster-1, the low-stroma subtype; cluster-2, the immunoglobulin subtype; cluster-3, the high-stroma subtype; cluster-4, the mucinous subtype; and cluster-5, the unclassified samples. The high-stroma subtype was characterized by increased levels of genes distinctive of tumor-associated stroma and components of the extracellular matrix. The mucinous subtype was characterized by the increased expression of trefoil factors and mucins and also by the high frequency of *BRAF* and MSI mutations [247]. The low-stroma subtype was associated with a better prognosis compared to the other subtypes [247].

Sadanandam and coworkers analyzed the gene expression profile of a large number of colon cancers and determined five subtypes according to the gene signature (the predominantly expressed genes defined each subtype): (i) goblet-like, defined according to the high expression of MUC2 and TFF3; (ii) enterocyte, defined by the high expression of the enterocyte-specific genes; (iii) stem-like, characterized by the high expression of Wnt signaling targets, stem cells, and myoepithelial, mesenchymal genes, associated with the low expression of differentiated markers; (iv) inflammatory, characterized by the high expression of inflammatory-related genes and chemokines; and (v) transit-amplifying, characterized by the variable expression of Wnt-target genes and stem cell genes [248]. It is of interest to note that 54% of the patients pertaining to the transit-amplifying subtype displayed a response to treatment with the anti-EGFR antibody cetuximab. Among this subtype, the sensitive tumors possessed high epiregulin and amphiregulin, which are positive predictors of cetuximab response; in contrast, filamin, which regulates expression and signaling of the c-Met receptor, was overexpressed in resistant tumors—in line with this observation, these tumors displayed increased sensitivity to c-Met inhibitors [248]. Analysis of the clinical outcomes of patients from whom the samples were derived showed the prognostic and predictive value of this classification: (i) the stem-like subtype was associated with a rapid recurrence when treated with surgery alone, but showed the greatest patient benefit from adjuvant chemotherapy; and (ii) goblet-like and transit-amplifying subtypes were associated with a favorable outcome in patients who received surgery alone, but were associated with a poorer outcome in patients who received adjuvant chemotherapy [248].

To explore the heterogeneity of colon cancers, De Sousa E Melo and coworkers analyzed 1100 colon cancers using an unsupervised classification strategy [249]. Using this approach, they demonstrated that three main molecularly-distinct subtypes can be recognized: (i) the first subgroup (CCS1-CIN) is typically associated with *KRAS* and *TP53* mutations and represents the group of chromosomal-instable tumors; (ii) the second group (CCS2-MSI) was strongly associated with MSI and CIMP; and (iii) the third subtype (CCS3-serrated) is heterogeneous for the MSI and CIMP status and relates to the sessile-serrate adenomas and shows the upregulation of the genes involved in matrix remodeling and the epithelial–mesenchymal transition [249]. This subtype is associated with an unfavorable prognosis and with resistance to EGFR-targeted therapy [249]. A subsequent joint study showed that these two classification schemes are not in conflict, but instead support eachother’s legitimacy. In fact, a simple correspondence between the two classifications was shown: the transit amplifying and enterocyte subtypes are subsets of the CCS2-MSI subtype, and the stem-like subtype is highly related to the CCS3-serrated subtype [250]. The correspondence between the two classifications is also supported by clinical criteria: CCS3-serrated tumors are resistant to EGFR-targeted therapy and a comparable trend is observed for the corresponding stem-like subtype of the other classification [250].

**Figure 9:**
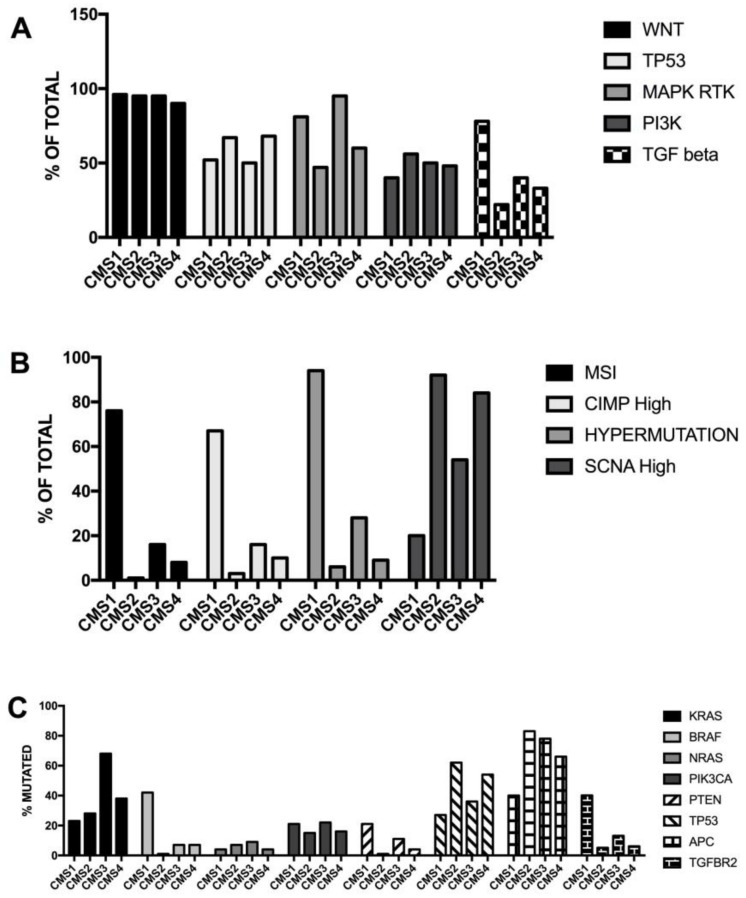
Gene expression-based classification of colorectal cancers into four types according to Guinney et al. [251]: consensus molecular subtype 1 (CMS1), CMS2, CMS3 and CMS4. (**A**): Activation of some signaling pathways in the four CMS subtypes. (**B**) Distribution of some molecular abnormalities in the four CMS subtytes. (**C**): Recurrent gene mutations in the four CMS subtypes. CIMP: CpG island methylator phenotype; hypermutation; SNCA high: single copy number alteration high.

A recent bio-informatics analysis of numerous gene expression profiles obtained through the study of thousands cases of colorectal cancer provided strong support for a novel classification of colon cancer, subdivided into four major CMS (Table 2, Figure 9): (i) CMS1 compounds that are hypermutated (about 14% of all cases), which are MSI-H, CIMP-H, CIN, *BRAF*-mutated (in about 40% of cases) and not very frequently mutated at the level of *APC* (≈35%), *TP53* (≈30%) and *KRAS* (≈25%) genes; these tumors predominantly originate from serrated lesions, located in the proximal regions of the colon and their prognosis is intermediate, but poor after relapse; (ii) CMS2 corresponds to the canonical subtype (about 40% of all cases), which are CIMP^−^, MSI stable, CIN-H, epithelial type, with frequent *APC* mutations (about 75%) and characterized by WNT and MYC activation; *BRAF* mutations are absent, while *TP53* mutations are frequent (about 70%) and *KRAS* mutations are moderately frequent (about 30%)—these tumors predominantly originate from tubular lesions located in the distal region of colon; (iii) CMS3 corresponds to the metabolic type (about 10% of all cases), which are not frequently CIMP^+^ (20%) and MSI (about 15% of cases), CIN-H in about 54% of cases and hypermutated in 30% of cases, with a gene expression pattern characterized by the upregulation of multiple metabolic signatures, with frequent *KRAS* and *APC* mutations; *TP53* and *BRAF* mutations are less frequently observed; these tumors are equally distributed in the proximal and distal segments of the colon and are associated with an intermediate prognosis; (iv) CMS4 corresponds to the mesenchymal type (about 25% of all cases), which are rarely hypermutated, MIS and CIMP^+^, but very frequently CIN^+^, with a gene expression signature characterized by upregulation of genes involved in epithelial-to-mesenchymal transition (EMT) and in TGF-β signaling, with frequent *APC*, *TP53* and *KRAS* mutations, but rare *BRAF* mutations; these tumors originate from serrated precursor lesions, predominantly localized in the distal segments of colon and are associated with a poor prognosis [251].

Subsequent studies were focused on better defining the heterogeneity and the biological features of the CMS subtypes of colorectal cancers. A recent study provided evidence that the BRAF-mutated group of colorectal cancer patients, mainly part of the CMS1 group, is heterogeneous and can be subdivided into two different subgroups that can be distinguished according to the pattern of gene expression. These two subtypes display distinct molecular patterns with one (BM1) showing high KRAS/mTOR/AKT/4EBP1/EMT/complement activation and immune infiltration, while the other (BM2) exhibits cell-cycle checkpoint dysregulation, glycolysis and mTORC1 signaling activation [252]. Interestingly, the BM1 colon cancer cells are more sensitive to the inhibition of BRAF, BCL2 and MEK compared to BM2; however, BM2 tumor cells are more sensitive to CDK1 inhibition, compared to BM1 [252]. Another recent study found that the expression levels of chemokine-like factor (*CKLF*) genes may be used as a prognostic stratification marker for CMS1 patients; in fact, CMS1 colorectal cancer patients displaying high CKLF expression, have a good prognosis compared to CMS1 patients showing medium/low CKLF expression levels [253]. CKLF is a factor released by leukocytes infiltrating colorectal tumors.

*KRAS* is a common canonical mutation in colon cancer, distributed among the various CMS. A recent study explored the impact of *KRAS* mutations on the immune microenvironment among the various colon cancer consensus subtypes [254]. Cytotoxic T lymphocytes, neutrophils, and the interferon-γ (IFN-γ) pathway are suppressed in *KRAS* mutants [254]. In particular, the immune response was heterogeneous among various *KRAS*-mutant colon cancers; *KRAS*-mutant CSM2 samples had the lowest Th1-centric coordinate immune response cluster, reduced expression of the IFN-γ pathway, signal transducer and activator of transcription 1 (STAT1) and motif chemokine 10 (CXCL10), and reduced infiltration of T lymphocytes and neutrophils, compared to CMS1 and CMS4 and to KRAS-WT CMS2 [254].

The development of a consensus regrding the molecular classification of colon cancer represents major progress in the study of this cancer. However, the complexity, the cost and the requirement of sufficient bulk tumors for the procedure required for the evaluation of the transcriptional profile of colon cancer impedes the widespread clinical use of this approach. A strategy to bypass all these limitations would consist in develop a simplified IHC approach, showing concordance with transcriptome-based classification. In this context, a recent study reported the development of a practical and robust methodology based on the IHC detection of five markers—CDX2, FRMO6, HTR2B, ZEB1 and KER—by IHC and microsatellite instability by standard molecular biology technology, to identify and classify molecular subtypes of colon cancer [255]. This classification based on IHC showed 87% concordance with the transcriptome-based classification [255]. The application of this methodology to three different data sets showed its capacity to identify the various CMS subsets and, particularly, CMS4 associated with poor prognosis [255]. Importantly, the application of this methodology to the tumor specimens obtained in the CAIRO2 study (in this study the colon cancer patients were treated with standard chemotherapy plus anti-EGFR antibodies) showed that among the KRAS-WT and BRAF-WT not all patients experienced benefits from the treatment with anti-EGFR antibodies: in fact, the benefit was limited to colon cancer patients with KRAS/BRAF WT epithelial-like tumors, but not KRAS/BRAF WT mesenchymal-like tumors [255].

Drug screening of tumor cell lines and patient-derived xenografts showed subtype-dependent response profiles, showing a response to EGFR and HER2 inhibitors in the CMS2 epithelial/canonical group, and high sensitivity to heat shock protein 90 inhibitors in cells pertaining to the CMS1 microsatellite instability/immune and CMS4 mesenchymal groups [256]. Furthermore, chemotherapy delayed the outgrowth of CMS2, but not CMS4 xenografts [257].

In all three classification systems of colorectal cancers based on the pattern of gene expression, one subtype was associated with a low degree of differentiation, the EMT and poor prognosis. Other studies have shown that these subtypes are specifically characterized by stromal cell signatures, related to the abundance in these tumors of cancer-associated fibroblasts, leukocytes or endothelial cells [258,259]. High expression of the stromal signatures was associated with resistance to standard therapies and poor prognosis [258,259]. Stromal cell signatures in colorectal cancers are associated with enhanced TGF-β signaling [258,259]. In line with these observations, *SMAD/TGF-β* alterations, observed in about 22% of colorectal cancer patients, are associated with reduced survival compared to the rest of patients not displaying these alterations [260]. These studies have shown the importance of the stromal component in the expression signature of colorectal cancers. By evaluating differentially-expressed genes between the central (CT) and the invasive front (IF) regions of tumors, Dunne and coworkers reached the conclusion that stromal-derived intratumoral heterogeneity can be a major confounder of transcriptomics-based CMS patient subtyping [261]. In a more recent study, the same authors, extending their previous analysis, reached the important conclusion that transcriptomic signatures based on cancer-cell-intrinsic gene expression overcome the confounding effect of tumor-microenvironment-related heterogeneity and group samples by patient-of-origin rather than region-of-origin [262]. Single-cell transcriptomic analysis showed that epithelial-to-mesenchymal transition-related genes were found to be upregulated only in the cancer-associated fibroblast (CAF) subpopulation of a tumor sample; furthermore, colorectal tumors previously assigned to a single subtype on the basis of bulk transcriptomics could be subdivided into subgroups with divergent survival probability using single-cell signatures [263].

**Figure 10:**
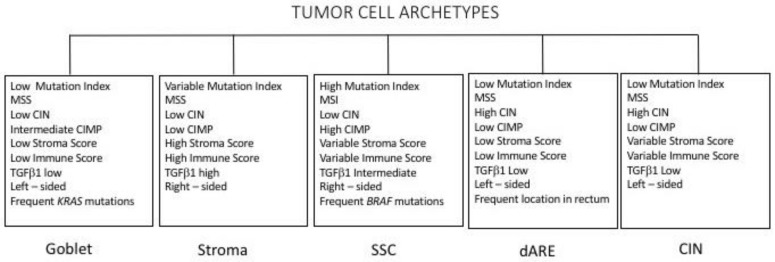
Classification of colorectal cancers into five tumor archetypes. This classification is based on gene expression data on the cancer cell and tumor microenvironment. This classification system is based on the study of Bramsen et al. [264]. SSC: serrated similar cancer; dARE: depleted AU-rich elements; CIN: chromosomal instability.

Bramsen and coworkers recently performed a biomarker-based study of colorectal cancer, reporting a classification system improving the prediction of patient prognosis [264]. The results of this study proposed a colorectal cancer classification based on cancer cell archetypes (derived from the analysis of the epithelial cell-specific transcripts) and tumor cell archetypes (derived from the analysis of tumor transcripts, relative to the epithelial and stromal cell components) (Figure 10). The analysis of epithelial-cell-derived transcripts and DNA methylation suggested the existence of three different archetypes (secretory type—about 25%—and absorptive type—about 50%—for their resemblance to secretory and absorptive enterocytes, and the serrated type—about 25%—due to its resemblance to sessile serrated colorectal cancer). (i) The secretory archetype showed typical features of secretory cell lineage, such as high ATOH1 mRNA expression and a mucosa gene expression patterns, extensive secretory goblet cell differentiation and an enrichment in *KRAS* mutations and signaling, a low mutation index, MSS and a low CIN score. (ii) The absorptive archetype showed classic features of conventional adenocarcinomas, including high CIN, MSS, low mutation index, enhanced WNT/β-catenin signaling, and low methylation. (iii) The serrated archetype was characterized by a high mutation index, MSI, CIMP-high, frequent right-sided location, frequent BRAF and KRAS mutations, and gene expression associated with immune processes (IFN-γ and inflammatory responses) [264]. In contrast, five different tumor archetypes were identified, more homogeneous for carcinoma cell-related features than for their heterogeneous stroma content: (i) goblet subtype, which is largely composed of the secretory cancer cell type (and CMS3 tumors) and exhibits the properties previously described for the secretory type; (ii) the stroma subtype, which is composed of the three archetypes of colon cancer (secreted, serrated and absorptive; in part corresponding to CMS4) and is characterized by high stroma and immune scores, high TGF-β expression, MSS, low CIN, variable mutation index, and frequent right-sided location; (iii) the SSC subtype, which largely corresponds and exhibits the properties of the serrated colon cancer archetype, including intermediate stroma and immune scores, and variable TGF-β expression; and (iv, v) the depleted in AU-rich elements (dARE) and CIN subtypes, which correspond to the absorptive colon cancer archetype and basically exhibit the properties described for this cancer-related archetype [264]. These two subgroups are subdivided according to their CIN properties and, mostly, according to the presence of a dARE phenotype. The dARE phenotype is enriched in antisense RNA/long non-coding RNA; AREs are frequently found in immune-related transcripts, where they facilitate the post-transcriptional degradation upon stimulation. Preliminary evidence was provided that the dARE archetype is microbiome-dependent [264]. Importantly, patients with stroma archetype tumors have a shorter relapse-free survival (RFS) than other patients and, particularly, those with SSC tumors, thus suggesting that the tumor microenvironment has a stronger impact on prognosis than colon cancer archetypes [264]. It is interesting to note that both poor-prognosis stroma tumors and good-prognosis SSC tumors are enriched in the expression of sets of genes related to the immune response: however, while SSC tumors are enriched in transcripts related to active cytotoxic CD4^+^ and CD8^+^ T lymphocytes and depleted in transcripts associated with activated stroma and immune suppression, the reverse process was observed for poor-prognosis stroma tumors [264]. In contrast, CIN and dARE tumors are relatively depleted in gene sets associated with stromal and immune activity. The goblet tumor microenvironment mostly resembled the normal colon mucosa, characterized by high plasmocytes and a high expression of immunoglobulin A (IgA) [264].

Since the CMS colorectal tumor classification implies the presence of a stromal component, this classification system cannot be used for the characterization of preclinical models (tumor cell lines, patient-derived tumor xenografts) devoid of the stromal component. For this reason, Eide and coworkers recently developed a CMS scaller, an algorithm for CMS classification in the absence of human tumor stroma [265].

The analysis of genes differentially expressed in colorectal cancers with or without metastasis allowed the identification of a 13-mRNA signature able to successfully discriminate high-risk patients. The prognostic value of this signature was independent of the tumor stage, postoperative chemotherapy and somatic mutations. Thus, this 13-mRNA signature was able to identify two subgroups of patients with clearly better or poorer prognoses within the various subgroups of colorectal patients subdivided according to whether they were positive for *BRAF* or *KRAS* or had an absence of these two mutations [266]. Finally, the 13-mRNA signature outperformed the other known gene signatures [266]. Interestingly, the analysis of the high-risk patients identified by the 13-mRNA signature revealed enrichment in drug resistance, cancer metastasis and stemness [266].

Some studies have explored epigenetic abnormalities occurring in colorectal cancers and have attempted to provide an integrated genetic and epigenetic analysis of these tumors. Shen and coworkers carried out DNA methylation of the promoter regions and observed that colon cancers can be divided into three groups—two CIMP^+^ and one CIMP^−^ (i) CIMP1 tumors are characterized by microsatellite instability (MSI, 80%) and *BRAF* mutations (53%) and rare *KRAS* and *p53* mutations (16% and 11%, respectively); (ii) CIMP2 is very frequently associated with *KRAS* mutations (92%), frequent p53 mutations (31%) and rare MSI and *BRAF* mutations (0% and 4%, respectively); and (iii) CIMP^−^ cases with a high and moderate rate of *p53* and *KRAS* mutation, respectively (72% and 33%, respectively) and rare *BRAF* mutations (2%) and MSI (12%) [267].

Interestingly, distinct gut microbiome species are associated with CMS of colorectal cancer; thus, the most highly-enriched microbial species associated with CSM1 included *Fusobacterium hwasookii* and *Porphyromanas gingivalis*; and CMS2 was enriched for *Selemonas* and *Prevotella* species, while CMS3 had few significant associations [268].

## 5. Animal Models of Colorectal Cancer

The development of mouse models has played an essential role in understanding the cellular and molecular bases of colon cancerogenesis. The most common model is the so-called *APC*^Min/+^ model, a multiple intestinal neoplasia model: in this model, an autosomal dominant mutation was generated by chemical mutagenesis, which induced a loss of function mutation in the mouse *APC* gene at the level of codon 850. In this model, the majority of polyps developed in the small intestine and only rarely progression to invasive cancer was observed. This model was particularly useful for the definition of genes that, in cooperation with *APC*, can accelerate and decelerate tumor development. All the alleles causing a loss of the capacity of APC to bind to β-catenin are tumorigenic for intestinal cells; however, some of these alleles differ from other alleles for some peculiar biological properties. Thus, another mutant, *APC*^1322T/+^ more closely mimics the mutation occurring in human cancer (*APC*^1309^): interestingly, the *APC*^1322T/1322T^ mice had more severe polyposis than the Min mice, but surprisingly the tumors developed in these animals had lower levels of nuclear β-catenin than Min tumors [269]. The lower levels of β-catenin were associated with a lower WNT activation, but with a higher expression of the WNT target and stem cell marker LGR5 and also of other stem cell markers. According to these observations, it was concluded that a submaximal level of Wnt signaling favors the stem cell phenotype and more efficiently promotes colon cancerogenesis. Other models were based on *APC* gene deletion: the deletion of both copies of the *APC* gene induced colon adenoma formation [270]. Subsequent studies have shown that *APC* loss in intestinal cells determines a progenitor-like phenotype, driven by the *WNT* target gene *MYC*: in fact, *MYC* deletion rescues the perturbed phenotypes of proliferation, differentiation and migration induced by *APC* loss [271]. Some *WNT/MYC* targets are essential for mediating the tumorigenic effects, such as focal adhesion kinase [272]. The study of *APC*-deleted mice models of colon carcinogenesis suggested that intestinal stem cells (e.g., LGR5^+^ cells) may act as the cells of origin for cancer when *APC* is deleted, or a constitutive β-actin is expressed; however, the induction of activation WNT signaling in differentiated cells induces de-differentiation and adenoma formation, but this process requires additional mutational oncogenic events (reviewed in [273]). A mouse model of colorectal cancer whereby *APC* can be conditionally suppressed provided clear evidence that *APC* restoration drives rapid tumor cell-differentiation and regression without the relapse and restoration of a normal crypt-villus homeostasis [41]. Using a Drosophila model, it was possible to demonstrate that the intestinal stem cell over-proliferation induced by the inactivation of the *APC* gene requires two evolutionary conserved cofactors. Earth bound (Ebd) and Erect wing (Ewg); particularly, these two cofactors are required for all the major consequences of *APC* inactivation in the intestine, including the hyperactivation of *WNT* target gene expression, increased number of intestinal stem cells and hyperplasia of the intestine epithelium [274].

The generation of metastatic models of colorectal cancer implied the generation of murine models of invasive colon adenocarcinoma. Mouse models combining mutations of *APC* with the expression of an oncogenic *KRAS*^G12V/+^ induced an increased number of intestinal tumors, endowed with increased tissue invasiveness [275,276]. However, it must be pointed out that the effects induced by the expression of one *KRAS*^G12D^ allele were variable in different studies. In fact, Forbes et al. used a CDX2 P-G22 Cre system to express the mutant *KRAS*^LSL-G12D^ allele into intestinal epithelial cells [277]. Using this system, it was shown that the expression of a mutant *KRAS* allele into mouse colonic cells induced the formation of lesions reminiscent of human hyperplastic polyps, but failed to induce the expansion of the intestinal stem cell population; in contract, inactivation of both *APC* alleles resulted in the expansion of the crypt stem cell population, generating an adenomatous epithelium resembling human colon adenomas [277]. In a subsequent study, Moon and colleagues showed that initiating events such as *APC* mutation may lead to the clonal expansion of colonic stem cells, while late, secondary genetic events such as KRAS mutations, further enhanced Wnt signaling induced by *APC* mutants by increased β-catenin stabilization [221,278]. Furthermore, it was shown that the expression of the intestinal stem cell markers CD44, CD133 and CD166 was induced in intestinal tumors only of bouble mutant *APC* defective/*KRAS*-mutant mice, thus indicating that *APC* mutation is required for CSC activation by oncogenic mutant *KRAS*.

Hung and coworkers developed a stochastic somatic mutation model of intestinal carcinogenesis based on the loss of *APC* and *KRAS*^G12D^/^+^ mutation; this model showed the progressive tumor development and the progression from adenoma to carcinoma [279].

Other studies based on *APC* mutation, in combination with the inactivation of various members of the TGF-β family—such as *TGFBR2, SMAD2, SMAD3* or *SMAD4*—resulted in the generation of invasive, but not metastatic, colon adenocarcinomas [280,281].

*APC*^Min/+^*TP53*^−/−^ mice displayed a tendency to develop tumors with a higher burden and with increased invasiveness [282]. Interestingly, the expression of a single allele of a gain-of-function mutant *TP53*^R172H^ (mimicking the human *TP53*^R175H^), resulted in the formation of invasive tumors [283]. Mutant *TP53*^R270H^ in cooperation with *APC* deletion induced intestinal tumor invasion through the acquisition of an invasiveness phenotype with a complex glandular architecture [284].

It is thus evident that it was difficult to develop genetically-engineered mouse models that accurately recapitulate advanced-stage disease in the anatomical location observed in the naturally occurring disease. These limitations were related to the location of tumor development (mainly the small intestine) of traditional mouse models harboring APC mutations/deletions and to the overall tumor burden, limiting the time necessary for malignant progression. Tissue-restricted Cre/loxP-based strategies can drive cancer-inducing oncogenes in the colon but require multi-allelic intercrossing and rarely show the progression up to the metastatic stage. In this context, a particularly interesting example is provided by a mouse model of colorectal cancer involving specific Cre expression in colonic epithelial cells to more closely mimic the disease development observed in humans [285]. This system took advantage of the specific expression of the carbonic anhydrase I (*CarI*) gene in colonic cells and a promoter/enhancer from the murine *CarI* gene was used to generate Cre-expressing transgenic mice [286]. Thus, using this technology, an inducible *Car*^CreER^ mouse model with Cre expression in the cecum and proximal colon was created; and the mutation of the genes that drive human colorectal cancer with the *Car*^CreER^ mouse elicited the development of tumors exclusively in the cecum and proximal colon [285]. The mutation of both *APC* and *KRAS* yelded microadenomas in both the cecum and proximal colon, which progressed to macroadenomas; aggressive carcinomas, endowed with some metastatic capacity into lymph nodes, developed in response to the combined induction of mutations of four driver oncogenic genes, including *APC, KRAS, TP53* and *SMAD4* [285]. Although inducible *Car*^CreER^ is a model for studying human intestinal cancers originating from the proximal colon, its major limitation is related to the incapacity of this model to reproduce all the steps of human colorectal cancers, particularly for the late stages [285].

Recent studies have shown the methodology for obtaining organoid long-term cultures from normal and neoplastic human colon tissues. The long-term culture of human adult intestinal cells remained difficult for a long time, but recent studies have identified and optimized culture conditions for the long-term growth of organoids derived from normal and neoplastic colon tissue. The development of these cultures is based on three essential findings: R-spondin1, a Wnt agonist and the ligand for LGR5, induces crypt hyperplasia in vivo; EGF induces intestinal proliferation; and Noggin induces a marked increase of crypt numbers. These findings have been used to develop in mice a long-term culture system that maintains crypt physiology [287]. Thus, the combination of R-spondin 1, EGF and Noggin in basement membrane extract (BME) sustains ever-expanding small intestine organoids, displaying a number of features comparable to those of the original tissues in terms of cell architecture and cellular composition [287]. These culture conditions have been adapted to the long-term culture of human colonic adenocarcinoma by adding nicotinamide, prostaglandin E2, A83-01 (an ALK-inhibitor) and SB202190 (a p38 inhibitor) [288]. More recently, van de Wetering and coworkers using this technology developed a biobank of paired organoids derived from adjacent tumors and the healthy epithelium of colorectal patients [289]. Using this technology, a success rate of about 90% was achieved in establishing organoids. At variance with healthy tissue-derived organoids, tumor-derived organoids displayed different morphologies, ranging from cystic to solid structures [289]. Importantly, tumor organoids displayed molecular abnormalities highly comparable to those observed in primary tumors [289]. The large majority of these organoids displayed mutations in the WNT pathway, including inactivation mutations in *APC*, *FBXW7*, *Axin2* and *FAM123B*, gene fusions of the Wnt agonistic *RSPO2* and *RSPO3* and *RNF43* mutations [289]. Finally, it was shown the organoid culture platform can be exploited for functional drug sensitivity studies at the level of the individual patient [289].

The normal colon organoid cell culture system can be used to explore the minimal mutational requirements for colon adenocarcinoma development. Thus, using this system, Li and coworkers showed that the full development of adenocarcinomas resembling those spontaneously occurring in humans requires the deregulation of four different genes, including *APC, p53, KRAS* and *MAD4* [290]. Two recent studies extended these initial observations using the CRISPR-Cas9-mediated engineering of human intestinal organoids. Thus, Drost and coworkers showed that the introduction of four mutated genes—*APC*, *TP53*, *KRAS* and *SMAD4*—into cultured human intestinal stem cells was sufficient to induce tumors with the features of invasive colon adenocarcinomas, whose growth is independent from exogenous growth factors [291]. Importantly, the combined loss of *APC* and *TP53*, although not sufficient for tumoral transformation, was sufficient for the appearance of extensive aneuploidy [291]. These findings were confirmed in a parallel study by Matano and coworkers, who showed that the introduction of multiple mutations in the tumor suppressor genes *APC, SMAD4* and *TP53*, and in the oncogenes *KRAS* and *PI3KCA,* as well as into human intestinal organoids, elicited the formation of invasive tumors growing independently from growth factors and able to grow in vivo after implantation under the kidney subcapsule in mice [292]. These cells could form micrometastases after injection into mouse spleen but failed to colonize in the liver. In contrast, engineered organoids obtained from human adenomas formed macrometastatic colonies in the liver [292].

Other studies have reported the development of tumor organoid libraries from large collections of human colorectal tumors [293]. The development of these libraries did take advantage of the improvement in the definition of the niche factor requirements of colorectal organoids [293]. In vitro and in xenografts, the organoids largely reproduced the histopathological grade and differentiation capacity of their native parental tumors [293]. The growth-independent growth is associated with the adenoma-to-carcinoma transition, reflecting the progressive accumulation of mutations and independence from exogenous factors [293]. The analysis of matched pairs or primary and metastatic organoids, displaying similar genetic profiles and niche factor requirements, showed that the metastasis-derived organoids exhibited higher metastatic activity [293].

Recently, various reports described the development of transplantation procedures of engineered organoids enabling the generation of metastatic mouse models of colorectal cancer [294]. Importantly, these organoid models recapitulate human colorectal cancer progression. In one study, to implant organoids into the colon, a strategy was used based on short-term treatment with dextran sodium sulfate, inducing transient colonic damage and establishing a niche for cell engraftment [294], using organoid cultures harboring oncogenic *APC, KRAS*^G12D^ and *TP53*^R172/−^. In this system, colorectal carcinoma progressed from localized adenocarcinoma, to local disseminated disease and to distant metastasis [294]; a deregulated WNT pathway was essential for the progression of metastatic colorectal cancer [294].

Other recent studies have reported organoid-based models of metastatic colon cancer. Thus, Fumagalli and coworkers developed a metastatic model of the orthoptic transplantation of engineered cancer organoids [295]. This model allowed the definition of the contribution of common colorectal cancer mutations to metastasis. Using this approach, it was shown that the combination of oncogenic mutations in the Wnt (*APC* deletion), EGFR (*KRAS*^G12D^), *P53* (*p53*^KO^) and TGF-β (*SMAD4*^KO^) signaling pathways allowed the development of poorly-differentiated adenocarcinomas, endowed with metastatic potential, both at the liver and lung level [295]. The analysis of various mutant combinations allowed the determination that the initiating *APC* and *KRAS* mutations act as the drivers of proliferation and tumor growth, while inactivating mutations in *SMAD4* block differentiation during tumor growth and being required for metastatic potential [295]. Importantly, these four mutations allow growth at the level of distant sites lacking these factors. These findings suggest that the metastatic process is a direct consequence of the loss of dependency of specific niche signals [295]. Roper and coworkers reported a CRIPR-Cas9-based gene editing and organoid transplantation approach that employs colonoscopy-guided mucosal injection for primary and metastatic tumor induction in mice [296,297]. *APC*-deleted and *TP53*-deleted and *KRAS*^G12D/+^ mouse colon organoids and human colorectal cancer organoids engraft in the distal colon and metastasize to the liver [297].

Nakayama et al. investigated the biologic properties of tumor organoids derived from the intestinal tumors of mice, which were *APC*-deleted and expressed two *TP53*-mutant (*TP53*^R270H/R270H^) alleles; these organoids were characterized by high tissue invasiveness and associated with a complex glandular structure, but unable to metastasize in vivo [284]. RNA sequence analyses of tumor organoids showed global gene upregulation by mutant *TP53*^R270H^, which was associated with the activation of inflammatory and immune pathways [284].

Sakai and coworkers generated mouse models harboring different combinations of key colorectal cancer driver mutations (*APC, KRAS, TGFBR2, TP53* and *FBXW7*) in intestinal cells to investigate their role in the development of primary tumors and metastases. *APC* deletion caused adenoma formation and the combination with a *TP53* mutation of *TGFBR2*-deletion-induced submucosal invasion; the addition of *KRAS*^G12D^ induced an EMT-like morphology and lymph node invasion; and *APC* deletion, in combination with *KRAS*^G12D^ and F*BXW7* mutation, was insufficient to induce submucosal invasiveness, but induced an EMT-like morphology [298]. The analysis of tumor-derived organoids showed that *KRAS*^G12D^ is critical for liver metastatic capacity following splenic transplantation when this mutation was combined with *APC* deletion plus *TP53* mutation or *TGFBR2* deletion and showed the highest metastatic capacity when *APC* deletion was combined with *KRAS^G12D^* and *TGFBR2* deletion [298].

The organoid methodology can also be used to explore the peculiar mutational signatures associated with colorectal cancer development. Thus, Drost et al. used CRISPR-Cas9 genome editing in human organoid cultures to decipher the mutational consequences of the introduction of the mismatch repair gene *MLH1* [299]. As expected, *MLH1*^KO^ organoids accumulated an increased number of mutational events, compared with *MLH1*^WT^ organoids, thus reproducing the key feature of MSI-H cancers [299]. This strategy was also applied to the study of gene signatures induced by *NTHL1* gene inactivation. The *NTHL1* gene encodes for a DNA glycosylase that is involved in the removal of oxidized pyrimidines through the inhibition of the base excision repair gene [299]. The germline homozygous mutation in *NTHL1* causes adenomatous polyposis and colorectal cancer [76,300]. The knockout of the *NTHL1* gene in human colon organoids induced a typical mutational signature [299].

The study of tumor organoids allowed the better definition of the role of WNT hyperactivation induced by *APC* loss on the perturbation of normal intestinal stem cell homeostasis. These studies were focused on determining the mechanism undergoing MYC activation by MYC hyperactivation, focusing on the E3 ubiquitin ligase Mule, also known as HUWE1: this enzyme mainly acts by targeting substrates for degradation by attaching lysine 48-linked polyubiuquitin chains; however, the K63-linked ubiquitination of c-Myc may promote its transcriptional activity. Loss of Mule exacerbates the *APC*^Min^ phenotype, inducing the formation of adenomas populated by intestinal stem cells; loss of Mule alone was sufficient to promote intestinal tumorigenesis; interestingly, loss of *c-Myc* in Mule^KO^ mice restores normal intestinal cell proliferation [301]. Mule/HUWE1 is able to directly target β-catenin and to promote its degradation to stop the WNT signal under conditions of hyperactive WNT signaling [302]. These observations are consistent with a model where Mule/HUWE1 loss promotes cancer by increasing MYC levels, DNA damage and genomic instability. This prediction was confirmed through the analysis of a mouse model of combined *APC* and *HUWE1* deficiency showing a dramatic increase in tumor initiation; the analysis of the mechanisms of tumor development in these models showed that the tumor phenotype was driven by MYC and rapid DNA damage accumulation, leading to the loss of the second APC allele [303]. Interestingly, the increased levels of DNA damage sensitized the HUWE1-deficient colorectal cancers to DNA damaging agents [303].

The tumor suppressor *FBW7* acts as the substrate recognition component of an SCF-type E3-ubiquitin ligase complex targeting several proteins, including c-Myc and c-JUN. *FBW7* is one of the most frequently mutated genes (about 10% of cases) in colorectal cancers and *FBW7* inactivation in *APC*^Min/+^ mice accelerates tumorigenesis [304]. The mechanism of tumor suppression by *FBW7* was intensively investigated in appropriate animal models. FBW7 levels are frequently decreased in colorectal cancers and the deubiquitinase USP9X acts as a positive regulator of FBW7 stability [305]. Reduced USP9X levels in colorectal cancer are associated with reduced FBW97 levels and poor prognosis [305]. Colitis-driven tumor formation is strongly accelerated in mice with intestine-specific deletion of USP9X, via a mechanism involving c-Myc accumulation [305]. Other studies have shown that *FBW7* loss in colon cells promotes chromosomal instability and tumorigenesis via Cyclin E1/CDK2-mediated phosphorylation of centromere protein A [306]. *FBW7* mutation/deletion mediates resistance in colorectal cancer to targeted therapies by blocking Mcl-1 degradation [307]. Mcl1 inhibitors increase the sensitivity of *FBW7*-mutant colorectal cancer cells to various targeting agents [307].

Interesting results were found in a recent study on *ARID1A*, the third most significantly mutated gene in human colorectal cancers, with the highest frequency (39%) in cancers of the MSI-H type. When *ARID1A* is absent, SWI/SNF is lost from thousands of enhancers that subsequently lose activity, while residual SWI/SNF complexes containing ARID1B bind the enhancers that remain active. Enhancers are major determinants of cell type specificity in gene expression; *ARID1A* loss impairs SWI/SNF control of enhancers bound by dominant transcription factors that activate the gene expression programs critical for development and differentiation. *ARID1A*^KO^ mice developed tumors exclusively in the colon, displaying features of adenocarcinomas [308]. At variance with *APC*^Min^ mice, *ARID1A*^KO^ mice developed tumors exclusively in the colon and not in the small intestine [308]. Furthermore, double-mutant mice showed that *ARID1A* loss is required to facilitate tumorigenesis driven by the inactivation of *APC* [308].

Recently, protocols were reported for the deriving of colonic organoids from differentiated human embryonic stem cells or induced pluripotent stem cells. Interestingly, colonic organoids were obtained from patients with familial adenomatous polyposis harboring gene mutations in the WNT-signaling-pathway regulator gene encoding *APC*, representing a unique platform for the evaluation of drug sensitivity [309].

### 5.1. Colon Cancer Metastases

As in other solid tumors, in CRC patients, metastases are the main cause of cancer-related mortality. Distant metastatic disease is present in about 25% of patients at diagnosis and about 50% of patients will develop a metastatic disease. Only some metastatic patients had metastases limited to a single organ and in some of these patients the metastases are resectable; however, many patients have multiple, non-resectable metastases. The liver is the most common site (50% of cases) of metastasis from CRC, due to venous drainage of the colon and rectum. The lungs are the second most common site (10–15% of patients). The peritoneum is another frequent site (4–13%) of CRC metastases.

The development of molecular biology techniques has allowed the evaluation of genetic similarities/differences between primary tumors and their metastatic lesions. A fundamental study by Jones and coworkers showed that the large majority of the somatic mutations observed in metastatic lesions are also observed in the corresponding primary tumors [39]. These findings are in line with the hypothesis that metastases are not seeded by single cells of the primary tumors, but by clusters of tumor cells [310].

In colon cancer patients, the presence of cancer cells in lymph nodes defines stage III disease. The association between the presence of lymphatic and distant metastases, together with the observation that lymph node dissemination often precedes distant metastases, has supported the current view that lymph node metastases in turn generate distant metastases. This concept represents the basis of the TNM staging system of tumors in which the primary tumors (T) initially seed regional lymph nodes (N) that in turn seed distant metastases (M). According to this view, the surgical removal of tumor-positive lymph nodes was currently performed. Given this background, recent studies performed on colorectal cancer were focused on evaluating the clonality of lymph node metastases and distant metastases.

Although the results generated in the various studies were not homogeneous, there now emerges an indication of the main mechanisms operating in lymphatic and distant metastasis formation. Thus, by comparing the *KRAS, BRAF* and *PIK3CA* mutations in primary tumors, Baldus and coworkers observed a consistent heterogeneity between primary and lymph node metastases (particularly for *KRAS*), but less consistent between primary and distant metastases [311]. However, the discrepancy between the lymph node and distant metastases could be artefactual and related only to the very limited number of distant metastases analyzed [311]. Other studies have shown a high concordance between primary tumors and metastases for *KRAS, NRAS* and *BRAF* [206,312,313]. The study of the association between the mutations of key driver genes and colorectal cancer metastases has been explored in many studies, in some cases generating contradictory results. A recent meta-analysis based on the main papers published on this topic has been performed [314]. This analysis provided evidence that mainly *KRAS* mutations and *TP53* mutations were associated with colorectal cancer metastasis; some of the studies also suggest a role for S*MAD4* and *BRAF* [314].

The limited heterogeneity of the distant metastases was also supported by other studies. Thus, Sebagh analyzed 157 patients with multiple liver metastases and observed pathological heterogeneity in 20% of cases; genetic heterogeneity was observed in 28% of patients with pathological heterogeneity [315].

Importantly, recent studies have in part clarified the clonality of lymph node metastases. Ulinz and coworkers have performed a detailed genetic analysis of primary tumors and paired lymph node metastases and have reached the important conclusion that lymph node metastases are polyclonal and can originate from multiple regions of the primary tumor [316]. Furthermore, a single lymph node metastasis can harbor subclones from different geographic regions in the primary tumor [316]. These findings were compatible with a model of tumor metastases, where multiple waves of metastatic cells escape the primary tumor over time and seed a single lymph node during the process of tumor progression [316]. A similar conclusion was reached by Wei and coworkers, who showed that all metastatic tumors inherited multiple genetically-distinct subclones from primary tumors, supporting a polyclonal seeding mechanism for metastasis; furthermore, metastatic tumors exhibited less intratumor heterogeneity than primary tumors [317].

Noxerova et al. performed a very interesting study based on the analysis of 17 CRC patients with lymph node and distant metastases; importantly, the study of somatic variants in hypermutable DNA regions allowed the reconstruction of the clonal relationship between the various metastases [318]. This analysis showed that in 65% of cases, lymph node and distant metastases arose from independent subclones in the primary tumor, whereas 35% of the cases share a common subclonal origin [318]. These observations indicate that the metastatic process in colorectal cancer is heterogeneous and two different lineage relationships between lymphatic and distant metastases exist [318].

### 5.2. Early-Onset Colorectal Cancer

The overall incidence of colorectal cancer exhibits a slow but constant decrease over the last years; in contrast, early-onset cancer (defined as occurring at an age of <50 years) shows an opposite trend, with a slow and continuous increase, with an incidence ranging from 3% (United States) to 8.6% (European Union) [319].

Two subtypes of early-onset colorectal cancer have been identified: an inherited subtype, occurring in the context of hereditary syndromes with a clear pattern of intrafamilial transmission; and a sporadic subtype, attributed to the presence of common/rare genetic variants, in large part unknown, predisposing and favoring the development of CRC [319].

The analysis of a large panel (450 early-onset patients) of patients showed the occurrence of germline mutations at the level of genes associated with various hereditary cancer syndromes in 16% of the cases [81,320]. Half of these patients with germline mutations had Lynch syndrome [81,320]. A total of 10.3% of these early-onset patients had MMR-deficient tumors, while 89.3% were MMR-proficient [81,320]. Among the MMR-proficient tumors, at least 8% had a germline in at least one gene (in some cases, mutations in high-penetrance colorectal cancer genes, such as *APC, PMS2, MUTYH, SMAD4*) [81,320].

De Voer and coworkers detected germline mutations of the RECQL-helicase gene *BLM* in a few early-onset colorectal cancer patients [321]. Biallelic mutations in the *BLM* gene causes Bloom syndrome, an autosomal recessive disorder characterized by chromosomal instability and increased cancer risk [321].

Another recent study identified two novel candidate genes for early-onset colorectal cancer susceptibility: the tyrosine phosphatase PTP-PEST, encoded by *PTPN12*, a regulator of cell motility; and *LRP6*, a component of the WNT-F2D-LRP6 complex that triggers WNT signaling [322]. Interestingly, the *LRP6* variants have been observed in individuals characterized by very-early-onset disease (<30 years).

Heald and coworkers reported the occurrence of germline *PTEN* mutations in some early-onset colorectal cancer patients [322]. These authors studied 127 patients with *PTEN* germline mutations; the germline *PTEN* mutations causes Cowden syndrome, which is associated with breast and thyroid cancers. A total of 13% of these patients had early-onset colorectal cancer [323].

Various studies have compared the molecular abnormalities observed in early-onset with late-onset colorectal cancers. These studies have shown that the main molecular abnormalities are basically similar in the two age groups, with some notable differences in the frequency of some of these abnormalities. In this context, McCleary et al. performed a study on a large set of colorectal cancer patients subdivided into three age groups—<60, 60–75 and >75 years—with the intermediate group being much larger than the other two groups. MSI-H, as well as CIMP-H, was more frequent in the older groups than in the younger group; the major mutational events, such as *KRAS*, *BRAF* and *PIK3CA* displayed similar frequencies in the three groups [324]. Interestingly, tumor nuclear CTNNB1 expression seemed to be associated with higher mortality among older, but not among younger patients [324]. However, other studies with a more appropriate cutoff (<50 years or <40 years) to identify early-onset colorectal cancers have shown an elevated MSI-H incidence (i.e., >15% of cases) in this group of patients [325].

Chang and coworkers investigated a very large set (1475 patients) of colorectal patients, subdivided into various age groups—<50, 56–60, 60–70, 70–80, and >80—importantly, 30 patients with Lynch syndrome were excluded from the data analysis [326]. The mutation rate of *APC*, *TP53*, *KRAS*, *PIK3CA*, *FBXW7*, *BRAF*, *NRAS*, *HRAS*, *TGFBR*, *AKT1* and *PTEN* was investigated; among these various genes, only *HRAS* and *PTEN* were more frequently mutated in younger (i.e., <50 years) patients than in older ones [326]. However, at variance with the previous study, MSI-H was higher among younger than older patients [326].

Kothari and coworkers investigated 246 colorectal cancer patients, subdivided into two age groups—<45 years and >65 years—and analyzed the mutation spectrum of various recurrent mutations in these two patient groups. Mutations in the *FBXW7* gene were more common in the younger cohort (27.5 vs. 9.7%), as well as in the proofreading domain of POLE (9.8% vs. 1%); in contrast, there were similar mutation rates between cohorts, concerning *TP53, KRAS* and *APC* genes [327]. The *BRAF* gene was more mutated in older than in younger patients [327].

Analysis of gene expression patterns showed differences at the level of 93 genes, differentially expressed in young and old colorectal cancer patients [328]; this analysis also showed that the RAS, MAPK, WNT and DNA repair genes are equally expressed in both age groups, whereas PI3K-AKT signaling is more specific to early-onset colorectal cancer and cell-cycle pathways to older patients [328].

In parallel, various studies have explored the clinicopathological features in early-onset colorectal cancer patients. Chang et al. analyzed a large set of colorectal cancer patients. In their study, the early-onset group of patients was defined as patients <40 years. The early-onset tumors were more frequently located in the distal colon (sigmoid/rectum), more frequently displayed adverse morphological features (signet ring cell differentiation), and perineural and venous invasion [329]. The two groups did not display differences in tumor stage, size and lymph node metastases and showed similar overall survival [329].

Yeo et al. investigated the clinicopathological features of early-onset colorectal cancers, defined with an age cutoff of <50 years [330]. The most relevant findings of these studies were that the incidence of early-onset colorectal cancer increased from the ascending colon to the rectum, more frequently presents with an aggressive histology (high-grade and signet ring cell) and with distant disease [330].

### 5.3. Genetic Abnormalities in Colorectal Neuroendocrine Carcinomas

The WHO established a classification system for colorectal neuroendocrine tumors based on clinicopathological characteristics. In 2010 this classification was updated and redefined to include four tumor types: colon neuroendocrine tumor (CNET) G1, CNET G2, colorectal neuroendocrine carcinoma G3 (CNEC G3) and mixed adeno neuro endocrine carcinoma (CMANEC) [331].

CNECs are a heterogeneous group of rare and highly aggressive colon tumors, subdivided into small-cell CNEC and large-cell CNEC. Recent studies have characterized the genetic abnormalities of these tumors. Morphologically, colorectal neuroendocrine carcinomas are a heterogeneous group ranging from small cell CNECs (SCNECs) to large cells (LCNECs). The mechanisms of carcinogenesis and the aggressiveness of CNECs remain unknown. CNECs are classified into three different subgroups according to their differentiation stage: G1, well-differentiated tumors are preferentially located at the level of the rectum and have a better prognosis than G3, not differentiated tumors, which are preferentially located in the colon [332].

In addition to CNECs, there are CMANECs, rare and heterogenous tumors, characterized by the presence of an exocrine and neuroendocrine compartment, each representing at least 30% of whole tumor cell population. Like CNECs, CMANECs are also aggressive tumors and require intensified treatment strategies.

Takizawa and coworkers comparatively analyzed the molecular characteristics of CNETs and CNECs, subdividing them into SCNEC and LCNEC [333]. Aberrant p53 expression (88%), β-catenin nuclear expression (48%) and high expression of cyclin E (84%) were much more frequent in CNECs than in CNETs (0%, 5% and 5%, respectively) [333]. *TP53, APC, KRAS* and *BRAF* genes were frequently mutated in CNECs, but not in CNETs, where no mutations of these genes were observed [333]. Although the molecular features of CNECs are similar to those observed in colorectal adenocarcinomas, decreased expression of Rb and high expression of p16 and Bcl-2 were more pronounced in CNECs than in colorectal adenocarcinomas [333].

Recent studies have characterized the pattern of genetic alterations observed in colorectal mixed adenoneuroendocrine carcinomas and neuroendocrine carcinomas, providing evidence that these tumors are genetically closely related to colorectal adenocarcinomas [334]. A total of 84% of these tumors harbor at least one somatic mutation and 11% displayed high microsatellite instability [334]. Compared with colorectal adenocarcinomas, neuroendocrine carcinomas exhibited a higher frequency of *BRAF* (37%) mutations, but a lower frequency of *KRAS* (21%) and *APC* (16%) mutations [334]. Other studies confirmed the frequent *BRAF* mutations in these tumors [335]; interestingly, the dramatic response to BRAF-MEK combination therapy was observed in two cases of metastatic high-grade neuroendocrine carcinoma refractory to standard therapy and displaying *BRAF* (V600E) mutation [336].

Olevian and coworkers comparatively analyzed poorly-differentiated CNECs and poorly-differentiated conventional colorectal adenocarcinomas for mutations in KRAS and BRAF and for DMA mismatch repair protein abnormalities. Compared to conventional adenocarcinoma, CNEC frequently exhibited *BRAF* mutations (59% vs. 5%) and less frequently displayed *KRAS* mutations (17% vs. 43%); the large majority (93%) of CNECs showed proficient mismatch repair [337]. At the clinical level, patients with poorly-differentiated CNEC had a significantly worse overall survival rate compared to patients with conventional colorectal adenocarcinoma [337].

Very interestingly, Woischke and coworkers performed a mutational analysis on the glandular adenoma/adenocarcinoma and neuroendocrine tumor components of CMNECs [338]. About 60% of these tumors displayed mutations at the level of codons 12 or 13 of *KRAS* gene exon 2. Interestingly, the *KRAS* mutational status was identical in the neuroendocrine and glandular components, thus suggesting a common clonal origin of CNECs and adjacent glandular components in colorectal tumoral tissue [338]. The examination of the overall mutation frequencies showed that about 33% of the mutations were shared among the glandular and NEC components, whereas about 40% occurred exclusively in the neuroendocrine component and 27% occurred selectively in the glandular component [338]. The most frequent mutations occurred in *TP53, KRAS* and *APC* genes and were shared between the two histological components [338]. The analysis of allele frequencies suggested that mutations in *TP53, KRAS* and *APC*, as well as individual mutations in *RB1*, *MET*, *BRAF*, *ERBB4* and *PTPN11* were identified as potential founding clone mutations [338]. Finally, the exome sequencing analysis of the glandular and NEC microdissected components, performed in a limited number of samples, showed the presence of shared mutations (9%), but most mutations were either exclusive to glandular (44.4%) or to NEC (46.6%) components [338]. The ensemble of these observations suggests a common clonal origin of the two components and their very early divergence and independent evolution [338].

### 5.4. Genetic Abnormalities in Small-Bowel Adenocarcinoma

**Figure 11:**
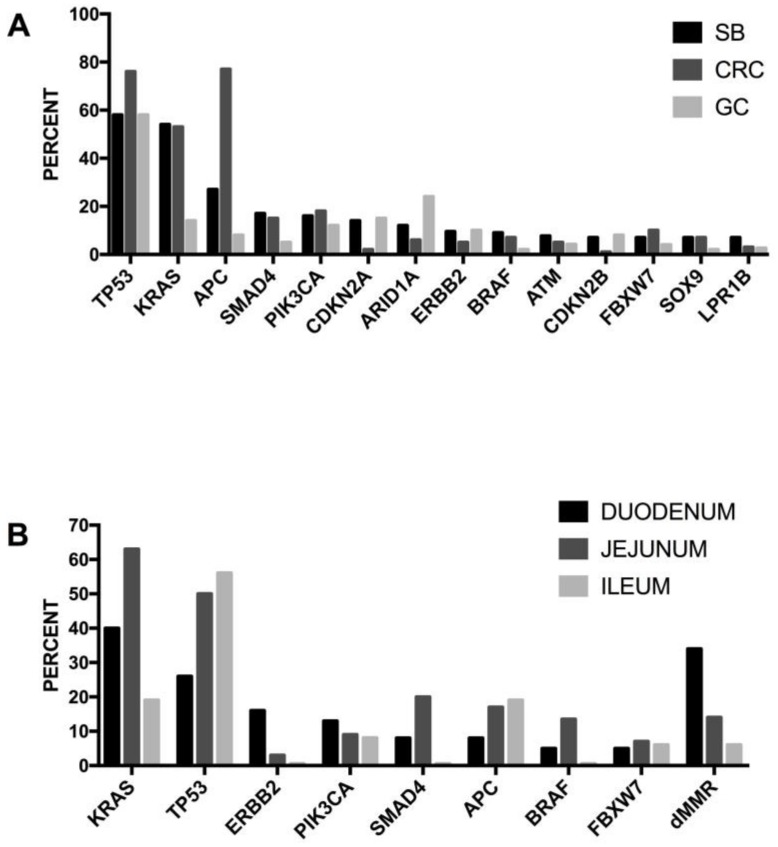
Recurrent genetic abnormalities observed in small bowel adenocarcinoma. (**A**): Frequency of genomic alterations in small bowel adenocarcinoma (SB), colorectal cancer (CRC) and gastric cancer (GC). (**B**): Frequency of genomic alterations in SB adenocarcinomas, subdivided according to tumor location. These data are reported in Schrock et al. [342].

Small bowel cancer accounts for about 1.5–2% of gastrointestinal malignant lesions and is a rare tumor, considering that the small intestine contributes to 75% of the length of the alimentary tract and 90% of its surface mucosal area [339]. Duodenal adenocarcinoma is the most frequent small-bowel cancer. This tumor is usually diagnosed at late stages of disease (III and IV), due to the absence of early symptoms and its inaccessibility for routine flexible endoscopy examinations. The pathogenesis of this tumor is scarcely known. Predisposing factors include Crohn’s disease, celiac disease, hereditary genetic syndromes and dietary factors [339]. Recent studies have in part clarified the molecular abnormalities typically observed in these rare tumors. A first study performed by French–German investigators reported the molecular characterization of a group of 83 small-bowel adenocarcinomas, showing that eight genes were mutated with a frequency of >5% in these tumors: *KRAS* (43%), *TP53* (41%), *APC* (13.2%), *SMAD4* (9.6%), *PIK3CA* (9.6%), *ERBB2* (8.4%), *BRAF* (6%) and *FBXW7* (6%). There is a relationship between the frequency of some of these genetic abnormalities and tumor location in the small intestine. Thus, *TP53* and *APC* mutations increased from the duodenum to ileum, while *ERBB2, PIK3CA* mutations and mismatch repair deficiency (dMMR) decreased from the duodenum to ileum (Figure 11) [340]. There is a strong association between *ERBB2* mutations and dMMR [340].

Yuan and coworkers confirmed in a small series of duodenal carcinomas that *KRAS* and *TP53* were the two most frequently mutated genes [341]. More recently, Schrock and coworkers reported the genomic profiling of small-bowel adenocarcinoma on a large set of patients (317 small-bowel adenocarcinoma patients) [342]. They reported a high frequency of *KRAS* mutations (53.6%) and of *APC* mutations (26.8%); genetic alterations of *SMAD4* (17.4%), *CDKN2A* (14.5%), B*RAF* (9.1%) and *ERBB2* (8.2%) were also frequent [342]. Interestingly, in BRAF mutant tumors, *BRAF*^V600E^ mutations—which are commonly observed in colorectal cancers—are rare and amount only to about 10% of these *BRAF* mutant tumors [342]. Microsatellite instability and hypermutation were observed in 7.6% and 9.5% of the tumors, respectively [342].

### 5.5. Genetic Abnormalities in Colorectal Carcinomas with a Signet-Ring Cell Component

Three major histological subtypes of colorectal cancer are commonly identified: intestinal type adenocarcinoma, mucinous adenocarcinoma (MAC) and signet-ring cell carcinoma (SRCC). The first histological—subtype I—is the most common (85–90% of all colon adenocarcinomas); MAC and SRCC represent 5–15% and 1%, respectively, of all colon adenocarcinomas. Most of the studies on these two more rare forms of adenocarcinomas usually involve the analysis of both these two tumors. The World Health Organization defines signet-ring cell carcinomas as tumors composed by >50% of tumor cells with prominent intracytoplasmatic mucin, displacing and indenting the nucleus. The presence of a signet-ring component, even in the range 1–50% and particularly when >50%, was associated with a poor prognosis [343]. Several studies have explored the molecular abnormalities of SRCCs. Ogino et al. have shown that *BRAF* mutations are more frequent in SRCCs than in MACs (28% vs. 22%), while *KRAS* mutations were more frequent in MACs than in STCCs (42% vs. 26%) [340]. *TP53* was also frequently mutated in SRCCs [344]. Other more recent studies have explored the frequency of *KRAS* and *BRAF* mutations in SRCCs subdivided according to the frequency of the singlet-ring component (1–49%; >50%); however, the results of these studies were discordant, and it is unclear whether a higher proportion of signet-ring cells is associated with a higher proportion of *BRAF* mutations [345,346].

Alvi and coworkers recently reported the molecular profiling of SRCC [347]. According to the methylation pattern, SRCC can be subdivided into hypermethylated (41% of cases) and hypomethylated (59% of cases). The hypermethylated group, which predominantly develops in the proximal intestine, is frequently *BRAF* mutated (about 80%), is enriched for CIMP and MSI phenotypes, and has high CD3^+^ cell infiltrates and expression of PDL1. The hypomethylated group predominantly develops in the distal colon and does not show any typical molecular features [347]. Interestingly, their study reported a high frequency of *KIT* mutations (1621A>C), particularly in the hypermethylated group of tumors [347]. The MSI^+^, CIMP^+^, *BRAF* V600E^+^/CD3^+^/PDL1^+^ hypermethylated genotype seems to be an ideal candidate for immune checkpoint inhibitor therapy [347]. Comparison with TCGA data for conventional colorectal cancer showed that SRCCs display higher frequencies of *TP53*, *BRAF* and *KIT* mutations, but lower frequencies of *APC, KRAS, PIK3CA* and *ATM* [347].

Interestingly, the analysis of SRCCs arising from colonic adenomas suggests that the signet cell component is derived from the molecular de-differentiation of the pre-existing adenomatous lesion [348].

## 6. Colon Cancer Stem Cells

### 6.1. Membrane Markers

The theory that cancer in adults derives from aberrant stem cells represents a modern interpretation of the so-called embryonal rest theory proposed by J Cohnheim in 1867 (mentioned in [349]). According to this theory, cancers arise from resident tissue stem cells or progenitor cells; therefore, tumors can be considered as aberrant organs, in which only a small subset of cancer cells, the cancer stem cells, initiate and maintain the tumor and are capable of metastatic spread [350]. The contribution of stem cells to tumor development was first noted in early studies of leukemia [351] and of teratocarcinoma [352]. Cancer stem cells, like normal stem cells, give rise to a cell progeny more or less heterogeneous, following the different types of tumors that are capable of various degrees of differentiation [353].

The research into colon cancer stem cells, as well as other tumors, is characterized by the identification of cell membrane markers useful for the isolation, amplification in vitro and characterization of these cells and by the development of in vivo assays. Two sets of membrane markers have emerged as the most useful for the identification of colon cancer stem cells: CD133 (Prominin-1) and CD144. CD133 is a five-transmembrane domain protein that is located at the level of the membrane protrusions of embryonal epithelial structures [354]. Given its peculiar location, a role was hypothesized for CD133 as an “organizer” of the plasma membrane topology [355]. However, to date, its function remains unknown. A role was tentatively proposed for CD133 in maintaining stem cell properties through an inhibitory action on cell differentiation [356].

Two different groups of investigators have identified human colon cancer initiating cells using CD133 as a marker [357,358]. These studies have shown that CD133^+^ colon cancer cells represent about 2.5% of the bulk tumor cells, are devoid of intestinal differentiation markers such as cytokeratin 20 (CK20), while expressing the epithelial adhesion molecule EpCAM. CD133^+^, but not CD133^−^ colon cancer cells are able to generate tumors of the same histotype when injected in nude mice; CD133^+^ colon cancer cells can be amplified in vitro as floating aggregates called tumor spheres, which can be maintained in vitro and are able to form colon cancers when injected into immunodeficient SCID mice; and upon growth factor deprivation, CD133^+^ cells gradually differentiate, become adherent and express intestinal differentiation markers, such as CK20 [357,358].

The identification of CD133 as a colon cancer stem cell marker was challenged by Shmelkov and coworkers [359]. In fact, these authors, using a knockin LacZ reporter mouse in which the expression of LacZ was driven by the endogenous CD133 promoter, showed that CD133 expression in the mouse colon is not restricted to stem cells and both CD133^+^ and CD133^−^ colon cancer cells could initiate tumorigenesis [360]. More recently, it was shown that single CD133^+^/CD24^+^ colon cancer stem cells can self-renew and reconstitute a complete and differentiated carcinoma [360]. Importantly, spheroid cultures of these colon cancer stem cells contain an expression of other stem cell markers, such as LGR5, CD44, nuclear β-catenin and CD166 [360]. Finally, Horst et al. have shown that the level of CD133 expression in colon cancer is a negative prognostic marker [361]. Using three different monoclonal antibodies against the CD133 antigen, these same authors have shown that CD133 positivity, coupled with nuclear β-catenin positivity, identify colon cancer cases associated with low survival [362]. They also showed that CD133-positive colon cancer cells are negative for antigens associated with epithelial differentiation [362]. Other studies have confirmed that the level of CD133 expression in colon cancer is a negative prognostic factor. Thus, Artells et al. analyzed CD133 mRNA levels in cancer tissue compared to normal colon tissue in 64 colorectal cancer patients, showing that expression levels were higher in the tumor than in normal tissue [363]. Furthermore, higher levels of CD133 were associated with shorter overall survival and relapse-free interval [363]. Finally, in a recent study carried out on 54 colon cancer patients, Horst et al. associated high CD133 expression with liver metastasis [364]. However, other clinical variables—such as age, gender, tumor size and histological grade—were independent of CD133 expression levels [360]. Furthermore, in this study, the authors depleted CD133 in the cultured colon cancer cell lines Caco-2 and LoVo, expressing high and moderate endogenous levels of CD133, respectively. CD133 knockdown does not affect the proliferation, migration, invasiveness and colony formation capabilities of colon cancer cells [364]. These observations support the view that CD133 is a marker with a high prognostic impact for colon cancer but does not seem to have an obvious functional impact as a driving force of this malignancy. In this context, Chen and coworkers reported the results of a meta-analysis carried out on all the data available on CD133 expression in colon cancer. The results of this meta-analysis showed that higher CD133 expression is significantly associated with poorer clinical outcomes and some clinico-pathological features such as tumor invasion, number of lymph node metastases and vascular invasion [365].

Srinivasan and coworkers showed that colon cancer stem cells are heterogeneous and can be subdivided into fast- and slow-cycling, which can undergo asymmetric division to generate each other [366]. Fast-cycling colon cancer stem cells express CD133, while slow-cycling colon cancer stem cells express Bmi1 [366]. NOTCH promotes asymmetric cell fate, regulating the balance between these two cancer stem cell populations [366].

Until recently, CD133 expression was regarded as restricted to undifferentiated colon cancer cells. However, a recent study showed that CD133 changes its conformation on differentiation, but not its level of expression; particularly, the decrease of AC133 epitope reactivity was due to a change in CD133 glycosylation, thus suggesting that CD133 is exposed on both colon cancer stem cells and in their differentiated progeny [367].

As mentioned above, high expression of CD133 has been used as a predictor for prognosis in colorectal cancers, suggesting that the enumeration of colon cancer stem cells using CD133, is a predictive marker for disease progression. To better understand the significance and prognostic value of CD133, Kemper et al. evaluated the expression of CD133 and other cancer stem cell markers by quantitative microarrays [368]. CD133 mRNA expression, but not that of other cancer stem cell markers, predicted relapse-free survival in colon cancer patients; interestingly, CD133 expression was related to mutations in *KRAS and BRAF* [369]. According to these observations it was concluded that CD133 expression is upregulated in colon cancers that have hyper-activated RAS-RAF-MEK-ERK as a consequence of mutations in *K-RAS* or *BRAF* [364]; since mutations in these genes are related to prognosis, it was concluded that CD133 expression is not indicative of cancer stem cell numbers, but rather dependent on the mutational status of the RAS-RAF pathway [370].

The other membrane marker used for the identification of colon cancer stem cells is CD44, a class I transmembrane glycoprotein acting as a receptor for constituents of the extracellular matrix, such as hyaluronic acid, and a downstream target of the Wnt/β-catenin pathway [370]. CD44 proteins regulate growth, survival, differentiation and migration and may be therefore involved in tumor progression and metastasis [371]. In colorectal cancer, the expression of CD44 is enhanced both in adenomas and in carcinomas [372]. Particularly, the expression of total CD44 and of CD44v3, CD44v6 and CD44v8-v10 isoforms correlates with a bad prognosis [371]. Furthermore, the invasion of colon cancer cells in Matrigel in vitro is dependent upon CD44 binding to hyaluronic acid and on the accumulation of hyaluronic acid in the pericellular region [373]. Colon cancer stem cells were shown to express the CD44 and the epithelial adhesion molecule EpCAM; furthermore, in some colorectal cancers, cancer stem cells were found also to express CD166 and the positivity for this antigen, associated with CD44 and EpCAM positivity, could be used for further enrichment of colon cancer stem cells [374]. It is important to note that immunohistochemical analysis of normal colon crypts shows that CD44 expression is not limited only at the level of the stem cell compartment at the crypt bottom, but also extends to the level of the cells forming part of the proliferative compartment. A subsequent study has validated CD44 as a robust marker of highly tumorigenic colon cancer cells with stem-cell-like properties [375]. Other stem cell markers, such as ALDH1, further enrich the cancer stem cell properties of the CD44 tumor population [375]. The value of CD44 as an important stem cell marker is also supported by a recent study showing the selective expression of the CD44v isoform in LGR5^+^ intestinal cells isolated from microadenomas of familial adenomatous polyposis patients [375]. Through knock-in techniques it was shown that CD44v promotes adenoma formation in APC^Min/+^ mice [376]. These findings support the view that CD44v is a marker of tumor stem cells and that plays an important role in promoting tumor initiation [376]. Constitutive activation of the β-catenin/TCF-mediated transcription by WNT pathway-activating mutations in human colorectal adenomas and adenomas of APC^Min/+^ mice results in a markedly enhanced expression of CD44v isoforms, including CD44v4 and CD44v6 [377]. Reduced adenoma formation was observed in mice CD44^−/−^/APC^Min/+^, which was completely rescued by the introduction of CD44v4 or CD44v6, but not by the standard CD44 isoform, CD44s [377].

An important function of CD44 in the biology of the normal epithelial intestine and in adenoma formation is related to its capacity to associate with receptor tyrosine kinases, such as EGFR and MET. A recent study provided evidence that CD44v could be involved in the mediation of the effect of MET, promoting intestinal crypt development and regeneration: in fact, intestinal crypts from CD44^−/−^ mice did not expand to the same extent as crypts of CD44^+/+^ mice on stimulation with hepatocyte growth factor (HGF, met ligand); this negative effect was removed by CD44v4 or CD44v6 expression [378]. Recent studies have shown that CD44v65 is expressed on colon cancer stem cells and is required to promote the migration and the metastatic potential of these cells [379]. HGF, osteopontin and SFD-1α (stromal-derived factor 1 α), secreted by cells present in the tumor microenvironment, increase CD44v6 expression by activating the WNT/β-catenin pathway [379]. CD44v6^−^ colorectal cancer cells may acquire CD44v6 expression and, concomitantly, develop a metastatic potential [375]. Heterogeneous nuclear ribonucleoprotein L-like (HNRNPLL), a pre-mRNA splicing factor, is a metastasis suppressor gene; its knockdown in colon cancer cells enhanced their tissue invasiveness and their metastatic potential and induced increased expression of CD44v6 [380]. The increased CD44v6 expression was required for the stimulatory effects of HNRNPLL on the metastatic activity of colon cancer cells [379]. HNRNPLL expression was downmodulated when colon cancer cells were induced to undergo the epithelial–mesenchymal transition [380]. CD44v6 is expressed in normal intestinal crypts and in hyperplastic polyps, with cellular labeling limited to the base of the crypts [380]. Tubulovillous, villous adenomas, as well as Stage I-III colorectal cancers express CD44v6 in the large majority (>90%) of cases; however, only 35% of metastatic colorectal cancers express CD44v6 [380].

Recently, the isolation of a monoclonal antibody was reported; mAbCC188 selectively targets a carbohydrate epitope expressed on the surface of both colorectal cancer stem cells and their differentiated progeny, while it does not bind normal colonic mucosa [381].

Pang et al. explored the expression of two markers—CD44 and CD26—in colorectal cancers and investigated the role of CSC subsets expressing these markers in tumorigenesis and metastasis [382]. Particularly, they identified a subpopulation of CD26^+^ cells present in both the primary and metastatic tumors in colorectal cancer patients with liver metastases. Importantly, in a group of patients without distant metastasis at the time of presentation, the presence of CD26^+^ cells in their primary tumors predicted metastasis on follow-up. Isolated CD26^+^ cells, but not CD26^−^ cells, led to the development of distant metastasis when injected into the mouse cecal wall; furthermore, CD26^+^ cells were also associated with enhanced invasiveness and chemoresistance [382]. This study suggests that the analysis of CSC subsets in the primary colon cancers according to CD26 expression may have important clinical implications as a selection criterion for adjuvant therapy. CD26 expression in colorectal cancers is observed particularly at late stages of tumor development and implies the emergence of a tumor cell population endowed with metastatic potential [383]. The CD26^+^ tumor cell population is maintained by genetic alterations at the level of *TP53* and *PIK3CA* [383]. In vitro studies have shown the generation of CD26^+^ cells from CD26^−^ cells [383].

A recent study suggested that different cancer stem cell subpopulations are responsible for the preferential metastatic capacity either to the liver or the lungs [384]. It is in fact well known that colorectal cancers preferentially metastasize to the liver; pulmonary metastases are less frequent and can occur after liver metastases or synchronously with liver metastases or metachronously with liver metastases. Gao and coworkers identified CD110 and CDCP1 as membrane markers of metastatic SCSs derived from human colorectal cancers that metastasized preferentially to the liver and lung, respectively [384]. Functional studies have shown that thrombopoietin interacts with its receptor CD110, which is present on CSCs, and stimulates their self-renewal and promotes the formation of liver metastases. CDCP1 acts by promoting the adhesion of colon cancer cells to the lung endothelium [384]. A subsequent study clarified in part the mechanism through which thrombopoietin (TPO) promotes liver metastases: this metastasis-promoting effect is mediated by the activation of lysine degradation, generating in turn acetyl-coA, used in p300-dependent LRP6 acetylation [385]. LRP6 acetylation triggers LRP6 phosphorylation at the level of tyrosine residues, activating the WNT signaling and self-renewal of CD110^+^ cancer stem cells [385]. Lysine degradation also generates other biological effects and determines the production of glutamate, which modulates the redox status of CD110^+^ cells [385]. The ensemble of these studies suggests that TPO is a component of the liver microenvironment required for the metastasis of colorectal cancer cells to the liver [385].

It is of interest to note that in all the studies related to the use of CD133 and CD44 as markers for the identification and isolation of colon cancer stem cells, the expression on these cells of EpCAM was noted. EpCAM is a glycosylated, 30- to 40-kDa type I membrane protein, expressed in a variety of normal and malignant epithelial tissues, described several years ago as a dominant antigen in human colon carcinoma tissue. EpCAM has a dual role as a cell adhesion molecule and receptor involved in the regulation of gene transcription and cell proliferation [386]. Some lines of evidence suggest that EpCAM is required for the proliferation of cancer stem cells. Particularly, it was suggested that cancer stem cells benefit from activated EpCAM for proliferation, self-renewal, and anchorage-independent growth and invasiveness [387].

A recent study showed that Rho kinase plays a key role in the regulation of CD44 expression at the level of colon cancer stem cells. In their study Ohata et al. showed that colon tumor spheroids possess a cell population displaying variable CD44 levels; CD44^high^ cells in these spheroids are characterized by an increased tumor sphere-forming capacity, constitutive β-catenin activity and enhanced glycolytic enzymes activity [388]. Interestingly, inhibitors of Rho kinase greatly enhanced the formation of spheroids from primary colon tumor cells [388]. It is of interest that the Rho inhibitor markedly enhanced CD44 expression at the level of some CD44^−/low^ cells that differentiate into CD44^high^ cells and acquire cancer stem cell properties [388]. According to these observations, it was suggested that the transition from the CD44^low^ to CD44^high^ state helps to maintain the CD44^high^ fraction and the tumorigenic heterogeneity in colon cancer [388].

As mentioned in the section on normal intestinal stem cells, recent studies have shown that ALDH1 could represent a marker for normal stem cells. Studies of the progression from normal to mutant APC epithelium to adenoma have found that ALDH1-positive cells increased in number and became distributed farther up the crypt [389]. ALDH1-positive cells isolated from colon cancers form tumors when inoculated in nude mice and fail to grow [389]. Interestingly, increased numbers of ALDH1-positive cells were found in the crypts of patients with chronic ulcerative colitis, a condition that predisposes people to colon cancer development through a pathway known as the inflammation–dysplasia–cancer progression [390]. ALDH1-positive cells isolated from these patients undergo a transition to cancerous stem cells after xenografting and can be propagated in vitro as tumor spheres [390]. Particularly, a ALDH^high^ cell population was identified in the normal-appearing, non-dysplastic colonic epithelium of colon ulcerative colitis (CUC) patients. Since these cells have the capacity to initiate the colitis–cancer transition, these cells were defined as precursor-CCSCs. These cells can be propagated in vivo as tumor xenografts and in vitro as non-adherent spheres [389]. However, the success rate for generating tumor spheres from ALDH^high^ cells derived from CUC patients is low [390].

Interestingly, cancer stem cells can be isolated not only from primary tumors, but also from colon cancer cell lines. In fact, it was shown that colon cancer cell lines contain cancer stem cell populations that can be enriched by the use of an in vitro, Matrigel-based differentiation assay together with selection for the expression of CD44 and CD24 cell surface markers. These CD44^+^/CD24^+^ cells are the most clonogenic in vitro and can initiate tumors in vivo [391].

A recent study provided evidence that ST6Gal-1, a sialyltransferase that adds sialic acid residues to the *N*-glycans of certain membrane receptors, is a marker of colon cancer stem cells [392]. ST6Gal-1 is overexpressed in several epithelial cancers, including colon carcinoma. In the normal colon, ST6Gal-1 is expressed at the level of the base of the crypts, where its pattern of tissue staining is comparable to that observed for ALDH1 [392]. It is of interest to note that also in other epithelial tissues, such as the epidermis, ST6Gal-1 expression was limited to the basal layer. Experiments of enrichment of colon cancer stem cells starting from colon cancer cell lines provided evidence of a concomitant enrichment of both ALDH1-positive cells and of cells displaying ST6 Gal-1 activity [391]. Experiments on the selection of colon cancer cells according to chemosensitivity showed that chemoresistant cells are enriched in both ALDH1-positive and ST6Gal-1-expressing cells [392].

Another approach to try to identify and isolate colon cancer stem cells consisted of the identification of the side population, i.e., of the cell population identified on the basis of its capacity of efflux of the DNA-binding dye Hoechst 33342. This property is usually related to an increased expression of ABC transporters. A limitation of the identification and isolation of SP cells using the DNA intercalating agent Hoechst 3342 is related to the capacity of this molecule to induce p53 and to cause cell death. An alternative method, producing comparable results, consists in the use of calcein, as calcein^low^ cells in large part correspond to SP cells. A recent study examined the cancer stem cell properties of calcein^low^ cells isolated from xenografts of colon cancer cells originated through the inoculation of colon cancer cell lines into immunodeficient mice. Calcein^low^ cells isolated from first xenograft passages were only marginally more tumorigenic than calcein^high^ cells; however, serial passages of these tumor cells showed that the calcein^low^ population became progressively enriched with self-renewal capacity in vivo, compared to calcein^high^ cells [393]. This calcein^low^ population was shown to contain high β-catenin nuclear expression [393].

Very interestingly, recent studies have suggested the existence of different subpopulations of colon cancer stem cells. Thus, Dieter et al. have reported a considerable heterogeneity in the colon cancer stem cell compartment, showing the existence of three different types of tumor-initiating cells in tumor spheres isolated from primary colon cancers: (i) long-term initiating tumor cells capable of extensive self-renewal and of maintaining tumor formation in serial xenotransplantation; (ii) tumor transient amplifying cells capable of limited self-renewal and generating tumors only in primary mice; (iii) delayed contributing tumor-initiating cells, capable of tumor formation only in secondary and tertiary mice [394]. Importantly, metastasis formation is limited only to long-term tumor initiating cells [394]. Finally, an important property of colon cancer stem cells consisted in their pronounced homing capacity at the level of bone marrow [394]. In a more recent study, the same authors investigated whether multiple, distinct genomic subclones with the properties of cancer stem cells exist within individual colorectal cancers and whether these genetic subclones determine the functional heterogeneity of colorectal cancer stem cells [395]. To investigate the genomic subclone kinetics in primary tumor samples and the corresponding serial xenografts and spheroids, high-coverage of the whole-genome sequencing analysis was performed [395]. This analysis, which was limited to the study of three patients, showed that two to four genetic subclones exhibiting differing growth dynamics provide different contributions to tumor growth over time [395]. Importantly, the various genetic subclones remained stable, but their individual contributions to tumor growth during time changed dramatically [395].

The development of the studies on colon cancer stem cells have led to the definition of the gene expression profiles of intestinal stem cells. These gene expression platforms can be used to screen colon cancers. Thus, Merlos-Suarez and coworkers defined a mouse intestinal signature obtained by identifying the genes markedly expressed in enriched populations of colon stem cells, compared to differentiated colon epithelial cell populations. This intestinal stem cell gene expression signature includes various genes known to be expressed in stem cells, such as the LGR5 gene (a Wnt target gene, marking proliferating colon stem cells) and is clearly associated with the colon cancer stage, as well as with tumor relapse and the occurrence of metastases [396]. This issue was re-explored in a more recent study, where colon cancers were screened using a colon cancer stem cell signature obtained determining the genes mostly expressed in colon spheres, compared to their differentiated progeny [397]. This colon cancer stem cell gene expression signature was intimately associated with disease recurrence in a set of 90 stage II patients undergoing curative surgery [397]. Although the gene signature for colorectal cancer stem cells was defined by high Wnt signaling activity, elevated expression of Wnt targets at the level of the primary tumors was associated with good prognosis, while patients with low expression of Wnt target genes were associated with immature stem cell signatures and exhibited a negative prognosis [397]. The reduced expression of some Wnt targets, such as LGR5 and ASCL2, during disease progression seems to be due to promoter methylation [397]. According to these observations, it was suggested that colon cancer stem cell gene signatures mainly reflect the differentiation status [397]. Recently, Ziskin et al. explored the expression of a series of genes—such as LGR5, Wnt7β-catenin, ASCL2 and BIM1—associated with intestinal stem cells into colon cancer samples. The expression of all these genes was evaluated in a large number (891) of colon adenocarcinoma patients. The large majority of colon cancers express both LGR5 and ASCL2 and the expression of these markers was positively correlated. However, LGR5 expression in this large group of colon cancer patients did not have prognostic significance [398]. LGR5 expression was explored in various types of colon tumors, particularly concerning the localization of LGR5^+^ cells within the tumoral tissue. Studies carried out using the in situ hybridization, the most suitable technique for the detection of LGR5 in human tissues, showed that: (i) adenomas displayed extensive LGR5 expression and this expression was not confined to the base of adenomatous crypts; (ii) in serrated lesions, LGR5 expression is upregulated, but the basal localization of LGR5^+^ cells is retained, with a crypt cellular organization resembling that of normal colon; (iii) in the majority of colon carcinomas with a glandular structure LGR5 expression is compartimentalized; and (iv) invasive colon cancer cell populations consist highly of LGR5^+^ cells [66,399]. However, these studies are difficult to interpret, due to the lack of reliability of the antibodies used to target LGR5. A recent study, based on the detection of LGR5 mRNA expression by in situ hybridization, provided a more accurate quantitative detection of LGR5 in human colorectal adenomas and adenocarcinomas [400]. In normal colon crypts, LGR5^+^ cells are exclusively located at the base of crypts and represents about 5–6% of the epithelial crypt cells; these cells are dramatically expanded in colon adenomas, either tubulovillous or tubular, representing >70% of total crypt cells; the proportion of LGR5^+^ cells is markedly increased in colorectal cancers, particularly in those that are moderately differentiated (60–95% of LGR5^+^ cells) and to a lower extent in those that are poorly differentiated (5–50%) [400]. These observations indicate that LGR5^+^ cell expansion is a hallmark of colorectal tumorigenesis, occurring during the early stages of the process of neoplastic transformation. The decrease of LGR5^+^ cells in poorly-differentiated colorectal cancers compared to those that are more differentiated suggests that the tumoral de-differentiation process involves the emergence of new de-differentiated cells [400]. These observations indicate that during the classical pathway of colon cancerogenesis there is an expansion of the pool of stem/progenitor LGR5^+^ cells; however, at the late stages of colon cancer development, decreased expression of LGR5 was reported. In fact, Zhou and coworkers showed that the maximum LGR5 expression was observed in stage I and II colon cancer and progressively decreased in stage III and IV colon cancers [401]. LGR5 knockdown experiments in colorectal cancer cells showed an increase of the metastatic potential of these cells both in the colon and in the lung [401]. LGR5 was shown to be capable of association with TGF-β receptors and its activation by R-spondin ligands determines the activation of LGR5 and the concomitant activation of TGF-βRII; furthermore, in colorectal cancer specimens, LGR5 expression correlates with SMAD2 expression [401]. These observations suggest that LGR5, in cooperation with TGF-β, exerts a suppressive effect on the metastatic activity of colon cancer cells [401].

Recent studies have better defined the role of LGR5^+^ cells in the development of human colorectal cancers. In a first study, Cortina and coworkers used the CRISPR/Cas9 technology to transfer lineage-tracing approaches to primary colon cancer cells (patient-derived tumor organoids) [402]. The analysis of LGR5^+^ cells isolated from organoid-derived xenografts showed that these cells exhibit a gene expression program similar to normal intestinal stem cells and propagate tumors to recipient mice with elevated efficiency [402]. Lineage-tracing experiments showed the capacity of LGR5^+^ cells to differentiate in absorptive and muco-secreting cell phenotypes [402]. Two groups have recently introduced metastasis-permissive models based on organoid technology, genome editing and orthoptic allogeneic transplantation [403,404]. This methodology was used to visualize the dynamics of LGR5^+^ cells in human colorectal cancer [403] and to investigate the roles of LGR5^+^ cells in mouse colorectal cancer [403]. Lineage tracing experiments have shown that LGR5^+^ cancer cells are capable of self-renewing and generate differentiated cell progeny; LGR5^+^ cells play an essential role in the growth of human colorectal cancers, however, upon depletion LGR5^+^ cells are replaced by new LGR5^+^ cells, originated from the de-differentiation of KRT20^+^ cells [403]. This observation indicates that LGR5^+^ cell-targeting therapy alone may be sufficient to eradicate colorectal cancer [403]. In the third study, De Sousa Melo and coworkers explored the roles of LGR5^+^ cells in murine models of colorectal cancer, showing that the ablation of LGR5^+^ cells in orthoptically transplanted tumors suppressed tumor growth only during cell ablation; in contrast, the elimination of LGR5^+^ cells in liver metastasis models led to the complete elimination of metastatic seeds [404]. These observations indicate the difficulty of developing a curative treatment for colorectal cancer based on cancer stem cell targeting and also show the hurdle that cancer cell plasticity poses in the attempt to achieve valuable therapeutic approaches [404].

### 6.2. Functional Properties and Signaling Pathways

Kreso and coworkers addressed two very important questions concerning the biological behavior of colon cancer stem cells. In fact, they addressed the question of the individual heterogeneity of tumor-initiating cells, evaluated both at the genetic and at the functional levels. Particularly, through a series of elegant experiments, they raised two essential questions: first, whether colon tumor-initiating cells form part of one genetic clone or are derived from distinct, different genetic subclones; and second, whether these cells display a functional variability in their tumor-propagating capacity. Thus, to explore the relative contribution of genetic and non-genetic mechanisms to the functional heterogeneity of single human cancer cells that are capable of tumor long-term propagation, they used an in vivo xenotransplantation assay in immunodeficient mice of individual tumor patient-derived cells, transduced with a green fluorescent protein (GFP)-expressing lentivirus to facilitate clonal tracking [405]. The results of these studies provided some important information about the heterogeneity of colon cancer stem cells. In fact, these authors showed that: (i) the genomic lesions detected in first xenografts were recapitulated upon serial xenotransplantation passages, a conclusion supported by exome sequencing; (ii) the analysis of methylation pattern diversity showed that the diversity of the various individual tumors remained in tumor xenografts over serial passages; (iii) the analysis of individual clonal variability in clonal repopulating activity showed that according to clonal longevity the clones can be subdivided in three subtypes: type I, long-term repopulating repopulating clones—type II, short-term repopulating clones; and type III, transiently-repopulating clones; and (iv) the analysis of individual variability in clonal repopulating ability displayed dynamic variability allowing the distinguishing of two typical behaviors: type IV clones—dormant clones forming small tumors in the first mice recipients, but large tumors in the secondary animals (these cells strongly change their growth capacity in time)—and type V clones—fluctuating clones, displaying a cyclic fluctuating variability of their tumor repopulating capacity [402]. This analysis showed that chemotherapy treatment significantly reduced the number of type I clones, but favored the growth of type IV clones, i.e., dormant tumor clones not growing in the untreated samples [405]. These clones do not acquire novel genetic mutations and therefore their treatment tolerance cannot be related to the acquisition of new driver mutations [405]. This very important study allows some important conclusions. Individual tumor cells forming a colorectal cancer are very heterogeneous in their functional properties in that they display variability in their repopulating capacity in terms of clonal longevity, dynamics and response to therapy. The tumor cells that at a given moment display repopulating capacity do not represent the whole population with these biological properties, since a part of them are initially dormant and acquire this property at a later time. The standard chemotherapy seems to spare these dormant clones that resist these drugs, and which are activated in their proliferative activity and become in part responsible for tumor resurgence [405].

Colon cancer stem cells, like normal intestinal stem cells, are capable of both self-renewing symmetric divisions (producing two CSC daughter cells) and asymmetric divisions (generating a CSC daughter cell and a differentiated non-CSC daughter cell). A recent study shows that the decision of a CSC to perform either a symmetric or an asymmetric cell division is regulated according to the level of miR-34a [406]. In fact, low miR-34a levels determine an upregulation of NOTCH signaling, thus promoting the maintenance of stemness at the level of daughter cells, while high miR-34a levels inhibit NOTCH signaling and promote the differentiation of daughter cells [406]. The critical role of miR-34a in this fine regulation consisted in converting “noisy” signaling inputs into the NOTCH bimodal level that enabled robust binary daughter–cell-fate decisions [406]. This mechanism of miR-34a-mediated asymmetric cell fate determination is mostly active in colon cancer stem cells isolated from early-stage colorectal cancer and is progressively lost in colon cancer stem cells isolated from late-stage specimens [406]. This mechanism may therefore represent a physiological protection from excessive intestinal stem cell proliferation during an oncogenic event. This conclusion was reinforced by a more recent study showing the existence in early-stage colon cancer stem cells of an incoherent feedforward loop, targeting both NOTCH and NUMB (an antagonist of NOTCH signaling causing its endocytosis and degradation) via miR-34a and favoring the asymmetric cell division of colon cancer stem cells [407]. LGR5^+^ mouse colon stem cells normally undergo symmetric divisions but switch to asymmetric divisions when proinflammatory stimuli or oncogenic events (APC loss) cause excessive proliferation of these cells; importantly, the deletion of miR-34a inhibits asymmetric divisions and exacerbates LGR5^+^ cell proliferation [407].

These observations suggest an important role for the NOTCH pathway in the control of colorectal cancer stem cells. Canonical NOTCH receptor signaling is controlled by an α-secretase, followed by intramembrane cleavage by a preselin-dependent γ-secretase to release the NOTCH intracellular domain (NCID). A disintegrin and metalloproteinase domain-containing protein 10 (ADAM10) or 17 (ADAM17) is required for NOTCH activation in the intestine and cell-autonomous ADAM10 signaling is essential for cell lineage specification and intestinal stem cell survival. NOTCH signaling is the dominant pathway activated by ADAM10 and ADM17. ADAM10-dependent NOTCH signaling controls the maintenance and survival of LGR5^+^ intestinal stem cells and regulates the cell fate specification of transit-amplifying progenitors; ADAM10 inhibition is associated with reduced stem cell proliferation and activity; furthermore, ADAM10 and NOTCH signaling plays an important role in maintaining quiescent Bmi1^+^ intestinal stem cells (reviewed in [408]). Studies carried out in experimental animal models have shown that NOTCH activation is able to promote, but not induce colon carcinogenesis. Thus, NOTCH activation alone in intestinal cells was unable to induce adenoma formation, but overexpression of activated NOTCH promoted adenoma formation and decreased the survival of an *APC*^+/−^ mouse model [409]. In contrast, the pharmacological inhibition of NOTCH using γ-secretase inhibitors blocked cell proliferation and induced secretory differentiation in established APC adenomas [410]. *ADAM10* deletion in APC biallelic loss models markedly reduced adenoma formation both in the small intestine and colon and improved animal survival; activated NOTCH rescued the ADAM10-deficient phenotype [411].

Other recent studies have further supported the heterogeneity of colon cancer stem cells and have raised some evidence that the phenotype of colon cancer stem cells may change as a function of the microenvironmental conditions. Thus, Kobayashi and coworkers established colon cancer stem cell lines starting from human colon cancer cells grown in NOG mice; these cells grow as adherent cells [409]. These cells were shown to express the stem cell marker LGR5 and to be capable of self-renewal [412]. Upon exposure to irinotecan, LGR5^+^ cells transposed to LGR5^−^, drug-resistant cells; in the absence of irinotecan, LGR5^−^ cells reverted to LGR5^+^ cells [412]. Both LGR^+^ and LGR5^−^ cells were able to generate tumors when transplanted into NOG mice [412]. Interestingly, epiregulin was expressed at the level of both LGR5^+^ and LGR5^−^ colon cancer stem cells [412]. Analysis of primary colon cancer tissues showed the existence of both epiregulin^+^ LGR5^+^ and epiregulin^+^ LGR5^−^ colon cancer stem cells: both these cell populations displayed a tumor-initiating capacity [409]. Other studies support the important role of the microenvironment in the induction and maintenance of the cancer stem cell phenotype. Thus, Lu and coworkers observed that endothelial cells secrete soluble factors that promote the cancer stem cell phenotype in colon cancer cells via NOTCH activation [413]. Immunohistochemical studies have shown that CD133 and NOTCH intracellular domain-positive colorectal cancer cells are co-localized at the level of perivascular areas [413]. The mechanism through which endothelial cells induced NOTCH activation is related to ADAM17 proteolytic activity; in line with this mechanism, the immunodepletion of JAGGED-1 in endothelial cell-conditioned medium abrogated the NOTCH-activating and the CSC-activating properties [413]. These observations suggest that the tissue microenvironment plays a major role in the maintenance of colon cancer stem cell properties. The angiocrine mechanism supporting the stemness properties of colon cancer stem cells imply a paracrine pathway, where the protease ADAM17 cleaves membrane-bound JAGGED-1 present on endothelial cells, releasing an N-terminal soluble fragment that binds and activates the NOTCH present on colorectal cancer cells [413]. These observations also open the opportunity to target angiocrine signaling via the inhibition of ADAM17 and/or soluble JAGGED-1. In line with these observations, the downregulation of JAGGED-1 induced the inhibition of cell proliferation, with reduced cyclin D1, cyclin E and c-Myc expression [411]; furthermore JAGGED-1 knockdown markedly reduced the growth of colon cancer cells in vivo in xenograft models, with marked downregulation of both proliferation and metastasis markers [414]. On the other hand, neutralizing anti-NOTCH1 antibodies blocked the growth of xenografts from colon cancer cells [415]. These studies highlight the importance of NOTCH pathway activation in the maintenance of colon cancer stem cells and in colon cancerogenesis. Interestingly, NOTCH1 was shown to activate the WNT/β-catenin signaling pathway in colon cancer [416]. In this context, the analysis of primary colon cancer samples showed that the activation of NOTCH1 is associated with the translocation of β-catenin in the nucleus [416].

A recent study provided evidence about an additional mechanism through which the NOTCH pathway is activated in colorectal cancer: The overexpression of the cargo protein MAP17 determines the neutralization by sequestration of the protein NUMB, an antagonist of NOTCH [417]. Interestingly, in colorectal cancers MAP17 expression correlates with a signature of NOTCH and stem cell genes [417]. NOTCH signaling, associated with bone morphogenetic protein signaling, are activated in poor-prognosis, mesenchymal subtype colorectal cancer [418]. The NOTCH pathway is activated in colorectal cancer cells also through epigenetic mechanisms, orchestrated by the overexpression of the signaling scaffold protein STRAP [419]. In clinical specimens, STRAP expression increased in colorectal cancers and its high expression was associated with poor prognosis [419]. Finally, there is a good correlation in tumor samples between STRAP and HES1 expression [419].

The large majority of the membrane cancer stem cell markers identified to date are also shared by normal stem cells; therefore, therapies that target these markers may cause severe tissue injury to normal tissues. However, recently Nakanishi and coworkers reported the identification of doublecortin-like kinase 1 (DCLK1) as a marker allowing us to distinguish between normal and tumor colon stem cells [420]. Previous studies have suggested that DCLK1, a microtubule-associated protein kinase, located in the normal intestine at the +4 position, above Paneth cells, could represent a putative intestinal stem cell marker; however, this view was challenged by other reports indicating that this marker is expressed in differentiated cells. In particular, it was shown that DCLK1 is a marker of Tuft cells. DCLK1-expressing Tuft cells constitute a unique intestinal epithelial lineage, distinct from enterocytes, Paneth cells, goblet cells and enteroendocrine cells. These cells are critical modulators of the intestinal stem cell niche [421] and critical mediators of the type 2 immune response to helminth infection [422]. Using lineage-tracing experiments, Nakanishi and coworkers provided evidence that DCLK1 does not mark intestinal stem cells in the normal intestine, but marks tumor-initiating cells, continuously producing a tumor cell progeny in the adenomatous polyps of Spc^Min/+^ mice [420]. Importantly, the ablation of the cells marked by tumor stem cell marker DCLK1 resulted in tumor regression without substantial damage to normal intestinal tissue [420]. In the mouse intestinal polyps, DCLK1^+^ cells in part co-express other stem-cell-associated markers, such as CD44, LGR5 and CD133 [420]; furthermore, DCLK1^+^ cells have been observed in human colorectal cancers [420]. Although these findings are interesting and offer therapeutic potentialities, future studies are required to assess whether DCLK1 marks cancer stem cells in primary human colon cancers and whether this marker really englobes the whole tumoral cell population endowed with tumor-initiating capacities. Interestingly, DCLK1^+^ tuft cells are quiescent and resistant to transformation, even after the genetic loss of APC and for long period of time. However, DCLK1^+^ cells are able to generate colon cancers when APC loss was followed by an inflammatory stimulus such as colitis: the effect of the inflammatory stimulus was active in inducing tumorigenesis when both were given immediately after APC loss or after three months [423]. These finding indicate that DCLK1^+^ cells remain quiescent even following an oncogenic mutation but are activated by tissue injury caused by an inflammatory stimulus and initiating colon cancer [423]. Importantly, the knockdown of DCLK1 cells in APC^Min/+^ mice attenuates intestinal adenomas and adenocarcinomas [424]. Recent studies provided evidence that DCLK1 promotes the epithelial–mesenchymal transition via the PI3K/AKT/NF-kB pathway in colorectal cancer cells [425]. Importantly, in some colorectal cancer models, the silencing of the expression of DCLK1 inhibited the invasion and metastasis by tumor cells [425]. DCLK1^+^ cells were widely distributed in colon cancer specimens, while DCLK1-positive epithelial cells are rarely detected in normal colon tissue [426]. DCLK1 mRNA was highly expressed in colon cancer stem cells but expressed only at low levels in normal intestinal stem cells [426]. Furthermore, studies carried out with human colon cancer stem cells support a key role for DCLK1 in maintaining tumor sphere growth in vitro and in vivo [426]. A subset of DCLK1^+^ cancer stem cells was able to overcome the inhibitory effects of chemotherapeutic agents via an autophagic survival mechanism; loss of DCLK1 combined with the chemotherapeutic/chemopreventive agents was required to achieve the eradication of cancer stem cells and to prevent tumor relapse [427]. A recent study showed that DCLK1 mediates the pro-survival signaling and self-renewal of colorectal cancers [428]. In fact, the TCGA Colon Adenocarcinoma Cancer Data shows the existence of a correlation between DCLK1 and pro-survival signaling expression [428]. The expression of DCLK1, as well as of other stem cell-associated markers (LGR5, Bmi1 and MUSASHI1), was clearly higher in epithelial cells of *APC*^Min/+^ than in WT controls; furthermore, enteroids from the intestinal DCLK1^+^ cells of *APC*^Min/+^ mice display high pluripotency and pro-survival signaling [428]. DCLK1 knockdown in *APC*^Min/+^ mice reduces adenoma formation and decreases self-renewal and pro-survival signaling [428].

The 5′(α)-promoter of the human *DCLK1* gene is epigenetically silenced during colon carcinogenesis, thus resulting in a loss of expression of the canonical long (L)-isoform 1 (DLK1-L) in human colorectal cancers, however, human colorectal cancers express a short (S) isoform 2 (DCLK1-S), generated from an alternate (β)-promoter of the *DCLK1* gene [429]. In normal enterocytes, the transcriptional activity of the β-promoter is potently inhibited by the transcription factor forkhead box D3 (FOXD3); in colorectal cancer cells, *FOXD3* is epigenetically silenced by methylation, allowing the expression of DCLK1-S [430]. Patients with high DCLK1-S levels exhibit significantly worse overall survival compared to those expressing low DCLK1-S levels [427]. Interestingly, in a retrospective analysis, adenomas derived from high-risk patients displayed higher staining for DCLK1-S than adenomas derived from low-risk patients [430]. These findings were confirmed through immunohistochemical analysis of adenomas with a DCLK1-S-specific monoclonal antibody, showing three- to five-fold greater reactivity in adenomas derived from high-risk patients, compared to those derived from low-risk patients [431]. In line with these findings, it can be stated that the overexpression of DCKL1-S into human colon cancer cells caused a significant increase of its invasive potential [430].

It is of interest to note that some studies have addressed the problem of the identification of differentially expressed stem cell markers in the early and late stages of colon tumors. In this context, Chen and coworkers observed that CCR9 is a chemokine receptor (CCL25). CCR9 expression was preferentially expressed in early-stage colon carcinoma and its expression decreased during disease progression, being low/absent in advanced colon cancers. Interestingly, the authors observed that adenomas of the colon were able to form tumors in recipient mice and thus the tumor-initiating capacity was considerably inhibited by the anti-CCR9 antibody [432]. In vivo CCR9^+^ cells induced tumor formation [429]. Blocking CCR9 signaling in vivo had a double effect: on the one hand inhibiting primary tumor growth, but on the other hand, increasing tumor metastasis [432]. In line with these findings, the stimulation of NOTCH signaling promoted the proteosomal-dependent degradation of CCR9 and promoted tumor spreading.

A recent study provided evidence of a possible role of a LGR5^−^ intestinal stem cells in the development of colon cancer. In particular, in order to to show whether an LGR5^−^ stem cell really contributes to colon tumor initiation, it established a genetic fate-mapping system for labeling Keratin-19 (Krt19)-expressing progenitor/stem cells; this system labels a population of cells including transit-amplifying cells, progenitors and stem cells, but excludes LGR5^+^ stem cells [433]. Using this system, it was shown that Krt19 marks long-lived radiation-resistant cells located above the crypt base that generate LGR5^+^ cells in a normal colon and intestine. Under conditional loss of the *APC* gene, Krt9^+^ cells display cancer-initiating abilities [433].

The studies aiming to elucidate the mechanisms responsible for the control of self-renewal and other biological properties of colon cancer stem cells require the availability of relatively large amounts of cells. This requirement, however, is difficult to meet because these cells are rare, and their in vitro expansion is difficult. To bypass these consistent difficulties, O’Brien and coworkers established an in vitro cell culture system is serum-free conditions using DMEM/F12 cell culture medium supplemented with EGF and bFGF: this cell culture system allowed the growth of tumor spheres that were more than 100 times enriched in colon cancer-initiating cells (CC-ICs) [434]. The expression of the stem-cell-associated markers—CD133 and CD44—in these cells was highly variable from one tumor sample to another and in the same sample at different times. In spite of their variable phenotype, these cells stably maintain their capacity to generate tumors in immunodeficient mice [434]. Using this cancer stem cell system, these authors explored the role of the helix–loop–helix transcription factors of the inhibitors of differentiation (ID) family in controlling the self-renewal of colon cancer stem cells. The results of these experiments provided evidence that ID1 and ID3 function together to control the self-renewal of colon cancer stem cells through cell-cycle restriction driven by the cell-cycle inhibitor p21 [434]. Colon cancer stem cells exhibited high ID1/ID3 expression and p21 expression; the knockdown of ID1/ID3 decreased p21 expression and decreased the tumorigenic potential of colon cancer stem cells [434]. Through this mechanism, colon cancer stem cells are protected from excessive DNA damage and from functional exhaustion [434]. Importantly, the silencing of ID1 and ID3 increases the sensitivity of colon cancer stem cells to chemotherapeutic agents such as oxaliplatin [434]. In addition to ID1 and ID3, ID2 expression is also induced in colorectal cancer cells by hypoxia [432]. Particularly, hypoxia induces WNT/β-catenin signaling, and through this mechanism stimulates cancer stem-cell-like phenotypes and ID2 expression [435]. Importantly, knocking down ID2 expression reduced CD44 expression and tumor-sphere formation and reduced the metastatic potential of colorectal cancer cells in vivo [435].

The studies performed on ID1–3 have suggested that the targeting of self-renewal could represent a strategy to inhibit colorectal cancer stem cells. This conclusion was further supported by another study showing that the targeting of Bmi1—a canonical regulator of self-renewal—inhibits the ability of colorectal cancer stem cells to self-renew, inducing the abrogation of their tumorigenic potential [436]. Importantly, the treatment of primary colorectal cancer xenografts with a small-molecule acting as a Bmi1 inhibitor, resulted in the loss of cancer-initiating cells, with a consequent inhibition of tumor growth [436].

Recent studies have shown that some transcription factors essential for the maintenance of embryonic pluripotent stem cells are also essential for colon cancer stem cells. Thus, in a first study, Leng and coworkers isolated a colon cancer stem cell line DLD-1 from colon cancer cells expressing typical colon cancer stem cell markers such as CD133, CD166, LGR5 and ALDH1 and by the high expression of the transcription factor KLF4, a transcription factor essential for maintaining the self-renewal of adult and embryonic stem cells [437]. The inhibition of KLF4 expression in these cells reduced the expression of cancer stem cell markers. In a second study, Maddox and coworkers showed that Oct1 is a transcription factor playing an essential role in the control of normal and pathological stem cell function. In the normal colon and small intestine Oct1^+high^ cells co-express markers of stem cells. Importantly, in colon cancer samples the number of Oct1^high^-positive cells correlated with the number of putative cancer-initiating cells CD44^high^CD24^low^ [438]. The reduction of Oct1 expression in colon cancer cells determines a clear decrease of ALDH1-positive and side population (SP) cells, while the opposite phenomenon is elicited by Oct1 overexpression [438]. According to these data it was suggested that Oct1 regulates the stem cell phenotype of colon cancer stem cells [438]. Other stem cell factors expressed in colon cancer stem cells orchestrate important properties of these cells and are important for the maintenance of the biological properties of these cells. Thus, Hwang and coworkers reported the high expression of the transcription factor SNAIL, an activator of the epithelial–mesenchymal transition [439]. Importantly, overexpression of SNAIL in colon cancer cells induced many properties of colon cancer stem cells, including cell de-differentiation [439]. This overexpression experiment showed that SNAIL induced a battery of genes related to IL-8 and JUN [439]; importantly, the blocking of IL-8 inhibited the inductive effect of SNAIL on cancer stem cell properties [439]. In primary tumors, SNAIL was co-expressed with IL-8 and CD44, but not with CD133 [439].

Other transcription factors contribute to the EMT in colon cancer cells. In fact, it was shown that the homeobox PROX1 is frequently overexpressed in colon cancers, where its overexpression correlates with E-cadherin downregulation [440]. The enforced expression of PROX1 in colon cancer cells caused the downregulation of E-cadherin and integrins, increased metalloproteinase activity and increased invasivity [440]. In another study it was shown that Axin2, a canonical Wnt suppressor, promotes EMT in colon cancer cells [441]. In fact, Wu and coworkers showed that in colon cancer cells Axin2 acts as a tumor promoter and not as a tumor suppressor; axin2 levels are increased in colon cancer cells, where this transcription factor acts as inducer of SNAIL activity, thereby inducing an EMT [319]. Silencing of Axin2 expression in colon cancer cells inhibits EMT and inhibit the metastatic and invasive properties of colon cancer cells [441].

The hairy and enhancer of split 1 (HES1) is a transcription factor belonging to the basic helix–loop–helix family of transcription factors. It is a transcriptional repressor of genes that require a bHLH protein for their transcription. HES1 plays an important role in the NOTCH signaling pathway. NOTCH signaling activates HES1 expression through a mechanism involving the classic pathway of NOTCH activation via the release of the intracellular domain of NOTCH. HESA1 targets the NOTCH ligands DLL1 and JAGGED1. The absence of HES1 in the developing intestine of mic promotes an increased differentiation to goblet cells, enteroendocrine and Paneth cells. Recent studies suggest an important role for HES1 in the regulation of normal and cancer stem cells. Thus, it was shown that HES1 is expressed in the crypt base columnar cells and transit-amplifying progenitors, but not in the Paneth cells or in other cells of the villi [442]. HES1 deletion in LGR5^+^ or Bmi1^+^ cells determines a loss of self-renewal, but does not perturb general homeostasis [442]. Furthermore, in normal LGR5^+^ cells, HES1 deletion and β-catenin stabilization decreased tumor formation and prolonged host survival [442]. Interestingly and importantly, in LGR5^+^ or DCLK1^+^ cells of established colorectal tumors, HES1 deletion induced immediate apoptosis, thus consistently reducing the tumor burden [442]. According to these observations, HES1 was considered a potential therapeutic target. Other studies support these findings. Thus, Nakata and coworkers showed that NOTCH ligand-dependent signaling is indispensable for stem cell proliferation and niche maintenance of APC-deficient intestinal tumors. In fact, the targeted deletion of *JAGGED1* in LGR5^+^ tumor-initiating cells resulted in the silencing of HES1 expression, the disruption of the stem cell niche and a dramatic reduction in the proliferative activity of APC-deficient intestinal tumors in vivo [443]. Finally, Gao and coworkers have shown that HES1 is upregulated in poorly-differentiated colon adenocarcinoma, compared with well-differentiated tumors; HES1 mRNA expression was increased in the majority of tumor samples, compared to the corresponding normal colon tissue [444]. Importantly, HES1 expression in tumor specimens correlated with the expression of stemness-related genes [444]. HES1 overexpression increased the size of CD133^+^ cells and the capacity of tumor sphere formation [444]. According to these findings, it was concluded that HES1 induces stem-like cell self-renewal and increases the number of cancer stem cells in colorectal cancer [444].

In addition to these findings, other studies indicate that some membrane markers used to identify colon cancer cells stem cells also play a role in the maintenance of the stemness properties of these cells as well as a role in the EMT. In this context, a role was defined for EpCAM and CD44. Concerning EpCAM, it was shown that EpCAM and transcription factors Oct4, Nanog, Sox2 and c-Myc are concomitantly expressed in colon TICs and EpCAM overexpression enhanced tumor sphere formation [445]. The knockdown of EpCAM inhibited several tumor properties and decreased the expression of *EMT* genes [445]. These effects of EpCAM seem to be mediated through NOTCH activation since they are inhibited by gamma-secretase inhibitors [445]. On the other hand, several recent studies suggest a link between CD44 expression and cancer stem cell properties and EMT. First, it was shown that TGF-β1, a well-known inducer of EMT and of colon cancer liver metastases, stimulates tumor sphere formation; TGF-β inhibitors reduced CD44 expression in tumor spheres [446]. Second, CD44v expression is inversely related to E-cadherin expression in colon cancer cells [447] and CD44 overexpression in colon cancer cells enhanced EMT markers [448].

### 6.3. Transcription Factors and Colon Cancer Stem Cells

APC mutation leads to β-catenin activation, which in turn induces a set of genes involved in the control of cell differentiation, proliferation and survival and, through this mechanism, promotes tumorigenesis. In this context, two recent studies have in part clarified the mechanisms through which β-catenin activation promotes colon tumorigenesis. Thus, genome-scale loss-of-function screens have shown that β-catenin active cancers are dependent on a signaling pathways involving the transcription factor YAP1 [449]. YAP1 is a transcription regulator that has been involved in stem cell differentiation and in the control of organ size. In particular, it was shown that YAP1 forms a transcriptional complex with the transcription factor TBX5 and β-catenin. The phosphorylation of YAP1 by the tyrosine kinase YES1 activates this transcriptional complex with its consequent localization at the level of the promoter of the anti-apoptotic genes *BCL2L1* and *BIRC5* [449]. In line with this mechanism, the addition of a small-molecule inhibitor of YES1 blocked the growth of β-catenin-dependent colon cancer cell lines and mouse colon cancer models [449]. This observation, together with previous studies, has led to the conclusion that constitutive β-catenin activation drives malignant transformations through interaction with two different transcriptional complexes: β-catenin/YAP1/TBX5 and β-catenin/TCF4. A second study was mediated through the binding of β-catenin to various transcription factors. In this context, recent studies have shown a relevant role of the transcription factor Forkhead box O3 (FOXO3a), for which β-catenin acts as a transcriptional co-activator, enhancing the expression of target genes [450]. FOXO proteins are phosphorylated by activated AKT and sequestered in the cytoplasm in an inactive form, with consequent inhibition of their transcriptional activity. The interaction between the Wnt pathway and FOXO transcription factors has important implications at the pathogenetic level, as well as for the development of new therapeutic strategies. Thus, Tenbaum and coworkers observed an accumulation of nuclear β-catenin and FOXO3a in colorectal cancers, and the highest nuclear concentrations of both these factors have been observed in stage 3 and 4 tumors and in patients with metastatic disease [450]. Experiments carried out in colon cancer cells expressing both activated β-catenin and activated FOXO3a showed that β-catenin diverts the FOXO transcription factor from its apoptosis-inducing activity to a metastasis-promoting activity [450]. In these cells, PI3K inhibition promotes the nuclear accumulation of FOXO3a and the metastasis of cells with high nuclear β-catenin content [450]. This finding explains why in these cells AKT/PI3Kinhibitors are unable to induce apoptosis and induce a paradoxical effect inducing tumor progression. Only the simultaneous inhibition of both β-catenin/Wnt and PI3K/AKT was able to induce apoptosis of these colon cancer cells [450]. Given these observations, experiments have been performed demonstrating that it is possible to evaluate the β-catenin activation status of a patient’s cancer cells and the response of these cells to agents targeting nuclear FOXO3a accumulation before taking any decision about a suitable individual treatment [450].

The role of YAP and TAZ transcriptional co-activators as regulators of the WNT pathway is complex. At the level of the cytoplasm, YAP/TAZ interact directly with β-catenin, hampering its nuclear translocation, and with dishevelled (DVL), inhibiting β-catenin activation. In the absence of WNT, β-catenin is degraded through a process requiring YAP/TAZ [451]. Therefore, cytoplasmic YAP/TAZ act as inhibitors of β-catenin signaling and, therefore, as suppressors of colorectal cancerogenesis. Inactivation of the β-catenin destruction complex, induced by APC inactivation, determines the stabilization of both β-catenin and YAP/TAZ, thus promoting their translocation in the nucleus [451]. In the nucleus, YAP/TAZ cooperate with β-catenin to transactivate WNT target genes [451]. Particularly, *YAP/TAZ* genes regulate the expression of target genes involved in stem cell self-renewal, cell proliferation and tumorigenesis. YAP/TAZ are required for the growth of adenomas following APC inactivation, and elevated YAP/TAZ expression is associated with poor prognosis in colorectal cancer patients [451]. The YAP/TAZ activity is antagonized both in the cytoplasm and in the nucleus by the T Cell Lymphoma Invasion and Metastasis 1 (TIAM1) co-factor [452]. In advanced colorectal cancer, TIAM1 is downregulated and low TIAM1 nuclear levels predict a poor prognosis [452].

Organoids may be used to enrich in cancer stem cells and to investigate the properties of these cells. Thus, Regan and coworkers have investigated the cancer stem cell content of a series of organoids established from colon cancer patients showing that they are enriched in cancer stem cells, whose number consistently changes from one case to another (being usually more frequent in organoids derived from tumors at an advanced stage) [453]. Using this cellular system and investigating the regulation of cancer stem cells present in tissue organoids, it was shown that cancer stem cell survival is regulated by non-canonical, Sonic HedgeHog (SHH)-dependent, PTCH1-dependent hedgehog signaling, which acts as a positive regulator of WNT signaling to inhibit cancer stem cell differentiation [453]. Hedgehog signaling in a normal colon is confined to the differentiated cells present at the top of the crypts, where it acts by antagonizing WNT signaling and limiting its expression at the base of the crypts. Hedgehog signaling may be activated by a canonical and non-canonical pathway. The canonical pathway involves the binding of the hedgehog to its receptor PTCH1, to relieve it from the repression of smoothened, frizzled class (SMO) receptor, which in turn activates a downstream signaling pathway, resulting in the activation of GLI transcription factors, exerting an activator effect on the transcription of some target genes [453]. Non-canonical hedgehog signaling does not act via the canonical hedgehog-to-GLi pathway and includes two different pathways: one works through PTCH1 and is independent of SMO, the other functions through SMO. Thus, the non-canonical hedgehog pathway is active in colorectal cancer stem cells, acting as a positive regulator of WNT signaling to regulate the survival of these cells [453].

As reported repeatedly above, canonical WNT/β-catenin signaling is essential to maintain intestinal stem cells and its constitutive activation is the earliest event during colorectal cancerogenesis. WNT signaling was found to be essential for the generation of cancer-initiating cells and two cancer stem cell markers—LGR5 and CD44—are known target genes of WNT signaling. Given this fundamental role in colorectal cancerogenesis and the maintenance of colon cancer stem cells, it was evident that drugs able to inhibit WNT signaling represent a potential new therapeutic tool. In this context, TNF receptor associated factor 2 (TRAF2) and NCK-interacting kinase (TNIK) are a fundamental regulatory component of the TCF4-β-catenin transcriptional complex and are the most downstream effector of WNT signaling. Recently, the first orally available small-molecule TNIK inhibitor was reprted—NCB-0846—possessing a potent anti-WNT activity; this compound binds to TNIK in an inactive conformation and this binding is fundamental to WNT inhibitory activity [454]. Importantly, NCB-0846 suppresses WNT-driven tumorigenesis in APC^Min/+^ mice and tumorsphere formation and the tumor-initiating capacity of colorectal cancer cells [454].

Several recent studies have explored the role of the secreted peptide progastrin in the regulation of the phenotype and the functional properties of colon cancer stem cells. Progastrin is secreted by human colorectal cancer cells and exhibits marked tumor-promoting activities on these cells. In particular, progastrin regulates the WNT and NOTCH pathways in colorectal cancer cells and through this mechanism plays a key role in the survival, self-renewal and modulation of the phenotype of colon cancer stem cells [455]. Given these properties of progastrin and its role as an autocrine regulator of colorectal cancer stem cells, it has emerged as a potential therapeutic target [456]. Thus, a recent study investigated the anti-tumor effects of a neutralizing anti-human progastrin antibody; one of these neutralizing antibodies was shown to inhibit cell proliferation and the migration/invasion of colorectal cancer cell lines to reduce the self-renewal of cancer stem cells and to increase chemosensitivity of colorectal cancer cells harboring *KRAS* mutations [456].

The analysis of the immunohistochemical expression of colorectal stem cell markers is often a subjective and semiquantitative process, leading to inconsistent and irreproducible reporting of results. To bypass these limitations, more objective methods for the analysis of protein expression in tissue samples and appropriate tissue selection are required. Automated digital platforms now offer the possibility of the objective analysis of protein expression in tissue samples. However, an additional important problem is related to the selection of regions of interest (ROI) for each tumor and for the control normal epithelium [457]. A recent study was particularly instructive, showing the variability of the optimal ROI to be analyzed for five different colorectal cancer stem cell markers (ALDH1, CD44v6, CD133, LGR5 and SOX2) [457]. Recent studies have explored the potential prognostic value of a set of colorectal cancer stem cell markers—CD133, LGR5, ALDH1, CD44v6 and SOX2—investigated alone or in association with immune-related cell markers—CD3, CD8, Foxp3 and PD-L1—in 104 stage III colorectal cancer patients [457]. The results of this study showed that the tumor expression of ALDH1, CD133 and SOX2 are nor correlated; elevated SOX2 expression is associated with a higher tumor grade and BRAF^V600E^ mutation; high CD133 and high SOX2 expression are associated with reduced overall survival; and high expression of immune markers CD3 and FoxP3 is associated with a better overall survival [458]. Interestingly, the combined evaluation of some stem cell and immune markers allowed the identification of subgroups of patients associated with particularly poor or good prognosis: patients categorised as SOX2^high^/PD-L1^low^ or CD133^high^/CD3^low^ have a poor outcome; and patients classified as SOX2^low^/FoxP3^high^ have a good outcome [458].

### 6.4. Colon Cancer Stem Cells and Chronic Inflammation

Many studies have suggested that chronic inflammation may play a significant role in colorectal carcinogenesis. This conclusion is supported by various pieces of evidence: (i) patients with CUC have a considerably increased risk of developing a peculiar form of colon cancer (colitis-associated cancer); (ii) in mouse models of colorectal cancer induced by APC loss of function, inhibition of two major enzymes responsible for the generation of secondary inflammatory mediators—cyclo-oxygenase 2 and inducible nitric oxide synthase—suppresses tumor formation; (iii) genetic and functional studies of inflammation-dependent models of colon cancer indicate that inflammation acts as a tumor promoter, although a direct role of inflammation in colon carcinogenesis through the induction of DNA damage cannot be excluded; and (iv) non-steroidal, anti-inflammatory drugs can reduce the risk of sporadic colon cancer and of familial adenomatosis-related colorectal cancer. Recent studies suggest that NF-kB activation could play a relevant role in the initiation of colorectal carcinogenesis. In fact, Shaked and coworkers used various models of mouse colon carcinogenesis [459]. Particularly, they showed that transgenic mice expressing constitutively active IkB kinase (IkkB) in intestinal epithelial cells developed intestinal tumors after a long period of latency; however, when these mice were crossed with mice with APC allelic loss, the resulting hybrid mice developed more β-catenin-positive early lesions and more colon tumors and reduced survival. Treatment of these mice with iNOS inhibitors decreased β-catenin lesions, DNA damage markers and reduced tumor size [459]. According to these findings it was suggested that NF-kB activation may accelerate APC loss of heterozygosity by enhancing nitrosactive DNA damage [459].

The role of NF-KB in colon tumor promotion and progression, as well as its contribution to tumor initiation and colon cancer stem cell function, was confirmed in another recent study [460]. In fact, it was shown that the constitutive activation of β-catenin in intestinal epithelial cells resulted in a rapid expansion of intestinal crypt cells and TNF-α-dependent NF-kB activation [460]. Inhibition of NF-kB in intestinal epithelial cells delays transformation in mice with constitutively active β-catenin and modulates its binding activity [460]. In contrast, elevated NF-kB signaling enhanced Wnt activation and induced de-differentiation of nonstem intestinal cells that acquire a tumor-initiating capacity [460]. These observations are very important because they provide a basis to explain the promoting effect of chronic inflammation on tumor initiation through an expansion of the tumor-initiating cell population [460]. Surprisingly, a recent study provided evidence that p53 loss could contribute to colon cancer development by favoring the generation of an inflammatory microenvironment. In particular, Schwitalla and coworkers showed that p53 loss is insufficient to initiate intestinal tumorigenesis in mice, but greatly stimulates carcinogen-induced intestinal tumor induction and triggers the formation of aggressive and invasive tumors [45]. The role of p53 during intestinal tumorigenesis is complex and different at early stages, compared to late stages of tumor development; thus, in the initial stages p53 exerts its orthodox effects on DNA damage and intestinal epithelial cell survival, while during tumor progression p53 loss is responsible for increased intestinal permeability, inducing the formation of an NF-kB-dependent inflammatory microenvironment and the epithelial–mesenchymal transition [45].

Other studies have provided evidence that cytokines pertaining to the IL-6 cytokine family play an important role in colorectal cancer formation. These cytokines signal through the common receptor chain gp130 and activate the JAK/STAT3 pathway. The gp 130/STAT3 pathway was shown to be essential for the pathogenesis of colitis-associated cancer. A recent study showed that IL-6, and particularly IL-11, play a potent pro-tumorigenic role in colorectal cancer models in mice [461]. It is important to note that ablation of the IL-11 receptor markedly reduced tumorigenesis in the APC^−^ mouse model [461]. Colorectal cancers develop proximally to microbial intestinal populations that are separated from immune cells by an epithelial barrier. The deterioration of the barrier occurring during the process of local tumor progression results in invasion of the tumor by microbial products that trigger tumor-elicited inflammation with the production of IL-17 and IL-23, which in turn drives tumor growth [462].

Not only familial adenomatous polyposis, but also other pathological conditions affecting the colon, predispose the development of colon cancer; under these conditions, Wnt pathway activation also seems to play a key role. This is the case for chronic ulcerative colitis. Patients with CUC have a markedly increased risk of developing a peculiar form of colon cancer (colitis-associated cancer). Recent studies indicate a consistent role for the Wnt/β-catenin signaling pathway in the colitis-to-cancer transition. Initial mutational studies at the level of the Wnt/β-catenin pathway suggested that the involvement of this pathway in the colitis-to-cancer transition is much less frequent than in sporadic colorectal cancer and occurs at later times in tumor history development. However, recent studies based on the characterization of cancer stem cells suggest a relevant role of Wnt/β-catenin pathway activation in the colitis-to-cancer transition. In fact, immunostaining studies have shown that Wnt activity levels in ulcerative colitis are intermediary between those observed in normal colon epithelial cells and in colon cancers [463]. A similar trend was observed for ALDH1, a marker of colon cancer stem cells. Showing intermediary levels between those observed in normal colon epithelial cells and those observed in colon cancer cells, interestingly, 52% of ALDH1^+^ cells in ulcerative colitis displayed Wnt activity [463]. ALDH1^+^ cells, particularly those displaying high Wnt activity, are able to initiate and maintain colon tumors when inoculated into immunodeficient mice [463]. Inhibition of Wnt activity into these cells reduced their tumorigenic activity (iii). These observations support an early role for Wtn activation in the colitis-to-colon-cancer transition and indicate that high Wnt activity may represent a marker for the identification of highly tumorigenic cells within the dysplastic epithelium in colitis patients [463].

The fact that chronic inflammation and tissue damage predispose the development of colon cancer suggests that some factors or pathways important for wound healing may promote tumorigenesis. Recent studies support this view. During an inflammatory response at the level of the colon epithelium, the sensing of the tissue damage by inflammosomes led to an IL-18-dependent decrease of IL-22 binding protein (IL.22BP) levels, with a consequent increase of IL-22/IL-22BP [464]. The enhanced IL-22 activity is important to promote wound healing; however, the prolonged IL-22 production and activity during the recovery phase promoted colon cancerogenesis, thus showing a link between inflammation/wound healing and colon tumorigenesis [464]. More recently, evidence was provided of an important role in colon cancer progression and stemness maintenance of another cytokine, IL-22. In fact, Kryczek and coworkers showed that IL-22 acts on colon cancer cells inducing STAT3 activation and the expression of the histone methyltransferase DOT1L. DOT1L expression was in turn responsible for the induction of the stem cell genes *NANOG*, *SOX2*, and *Pou5F1*, resulting in increased cancer stemness and tumorigenic activity [465]. DOT1L expression in tumor tissues was predictive of poor patient survival [465].

### 6.5. Cancer Stem Cells in Colorectal Neuroendocrine Cancer

As mentioned in the section concerning colorectal neuroendocrine cancer, the majority of studies suggest that these tumors display the same recurrent mutations observed in colorectal adenocarcinomas and suggest a common origin of these tumors, which often exhibit a glandular component together with a neuroendocrine component. Although the cancer stem cells of these tumors were not characterized, recent studies of normal colon neuroendocrine progenitors/stem cells led to a new evaluation of the biological properties of these cells. In 2013, Buczacki and coworkers used label retention properties to characterize the quiescent intestinal stem cell population [6]. This approach led to the identification of a population of slowly-cycling secretory progenitors in the +4 position of the intestinal crypt [6]. These cells express the LGR5 marker but are committed to secretory lineage differentiation and are capable of differentiation into neuroendocrine cells and Paneth cells [6]. These cells, however, retain the stem cell competence and are capable of regenerative activity in a condition of regenerative request [6]. Basak et al. investigated in vitro the signaling pathway capable of inducing the quiescence of LGR5^+^ cells and of driving their differentiation into enteroendocrine cells [466]. Using murine intestinal organoids, these authors showed that in the presence of sustained WNT signaling, inhibiting EGF or downstream MAPK activity induced a quiescent state into LGR5^+^ cells [466]. RNA sequencing studies showed that these quiescent LGR5^+^ cells switch to a secretory progenitor signature, including a marked increase of chromogranin A expression, a marker of enteroendocrine cells [466]. The coordinated inhibition of WNT, NOTCH and EGF/MAPK promoted enteroendocrine differentiation in organoid cultures [466]. Other studies have comparatively analyzed the transcriptomes of various intestinal stem cell populations, including single-cell transcriptomic analyses, showing that the large majority of multiple intestinal stem cell cycling populations resemble LGR5^+^ intestinal stem cells, with the exception of label-retaining Bmi1^+^ (Bmi1-GFP^+^) cells showing an enrichment of enteroendocrine markers, including Prox1 [29]. Prox1^+^ cells are capable of sustained clonogenetic growth in vitro and displayed in vivo a long-live repopulating capacity, as determined by lineage-tracing experiments, both in homeostatic conditions and after radiation-induced injury [29]. Single-cell transcriptomic analysis revealed the existence of two subsets of Prox1 quiescent cells, one resembling mature enteroendocrine cells, while the other displayed low-level of enteroendocrine-specific gene expression and co-expressed the Tuft cell markers—LGR5 and ASCL2—similar to the label-retaining secretory progenitors described by other authors [29]. According to these observations, it was concluded that enteroendocrine cells, including mature enteroendocrine cells, comprise a reservoir of homeostatic and injury-inducible intestinal stem cells [29]. This conclusion was confirmed in a parallel study by Jadhav and coworkers, who identified an active enhancer signature that distinguishes LGR5^+^ intestinal stem cells from Bmi1^+^ quiescent intestinal stem cells and other secretory cells and, through this analysis, reached the conclusion that Bmi1^+^ quiescent cells are preterminal enteroendocrine cells [30]. In particular, through this study, the authors conclude that Bmi1^+^ quiescent cells in mice resemble label-retaining cells that differentiate predominantly into Paneth cells, but also into enteroendocrine cells [467].

Enteroendocrine cells have been characterized in detail in the small intestine. These cells are important sensors of nutrients and microbial metabolites that secrete diverse hormones. According to their secretory capacities, enteroendocrine cells have been classified into eight distinct sublclasses, with cells expressing secretin (S), cholecystokinin (I), proglucagon (L), glucose-dependent insulinotropic polypeptide (K), somatostatin (D), neurotensin (N), grelin (A), and serotonin (enterochromaffin cells) [468]. A recent study showed that single enteroendocrine cells have multiple capacities of hormone production and three subsets have been identified according to their hormone secretory capacity: SILA, SIK and SAKD [469].

These studies have contributed to improving our understanding of intestinal neuroendocrine tumors. Neuroendocrine tumors are the most common tumors of the small bowel, but are rarer in the large bowel, accounting only for a minority of colon cancers. Recent studies have in part clarified the somatic mutational spectrum occurring in small-intestine neuroendocrine tumors. These tumors displayed a mutation rate of about 0.1 somatic single nucleotide variation/10^6^ base pairs in the exome, lower than that observed in colorectal cancers [470]. The mutated genes are *FGFR2*, *MEN1*, *HOOK3*, *EZH2*, *MLF1*, *CARD11*, *VHL*, *NONO*, *FNCD2* and *BRAF* [470]. *SMAD* genes were recurrently mutated or deleted in 45% of these patients, thus implying the TGF-β signaling pathway as an important determinant of these tumors [471]. The *CDKN1B* gene was either mutated or deleted in small bowel neuroendocrine tumors, implicating cell cycle dysregulation in the etiology of these tumors [471].

The origin of neuroendocrine tumors of the colon remains to be determined [472], but the recent acquisitions of colon enteroendocrine cells strongly support a possible origin of these tumors from either LGR5^+^ quiescent +4 progenitors or from Bmi1 quiescent cells. Interestingly, the conditional deletion of β-catenin in immature cells expressing the transcription factor neurogenin 3 induced small intestinal adenomas expressing serotonin; however, β-catenin deletion at a later stage of enteroendocrine differentiation did not result in the generation of adenomas [473].

## 7. Conclusions

Colorectal cancer accounts for over 8% of all deaths annually worldwide. Colorectal cancer is a heterogeneous disease with different mechanisms of pathogenesis. Advances in molecular biology over the past two decades have enabled a better understanding of the molecular pathogenesis of colorectal cancers and have permitted the introduction of innovative targeted therapies for the treatment of this tumor. Particularly, these molecular studies have led to the identification of driver genetic events in disease development, of molecular subtypes of colorectal cancers and of the consistent inter-tumor and intra-tumor heterogeneity.

The study of the molecular abnormalities of colorectal cancers has led to the identification of several genes, the alterations of which are involved in tumor initiation and/or progression. The model of the progressive step-wise accumulation of genetic and epigenetic events required first for the development of adenoma and then of adenocarcinoma was instrumental for the identification of driver somatic mutations occurring at the level of some tumor suppressor genes (*APC, TP53, SMAD4*) and oncogenes (*KRAS* and *PI3KCA*). The mutations of *APC* genes represent the earliest events in this pathway of colon carcinogenesis. The non-random accumulation of these genetic alterations leads to deregulation in the WNT, TGF-β and EGFR and downstream MAPK and PI3K signaling pathways, with consequent deregulation of cell proliferation, survival/apoptosis and differentiation. Studies in animal models, including colon organoids, have shown that alterations of these genes are able to both initiate and to drive tumor progression; however, full cancer development also requires the development of chromosome instability, causing loss of heterozygosity and imbalance in the chromosome number. Some mutations, such as *TP53* mutations, favor the development of CIN. This pathway of cancerogenesis is responsible for the development of about 85% of colorectal cancers. The remaining 15% of colorectal cancers develop through an alternative pathway of colon cancerogenesis, involving a defective mismatch repair system, due to the inactivation of genes such as *MLH1*, *MLH3*, *MSH2*, *MSH3*, *MSH6*, *PMS1*, *PMS2*, determining a high rate of somatic mutations (hypermutation) and microsatellite instability; these tumors are also characterized by a high CIMP (CIMP^high^), determining the hypermethylation of some genes, such as *MLH1*, involved in tumor development. Colorectal cancers exhibiting MSI have different molecular properties compared to the majority of the tumors associated with MSS. In fact, these tumors display frequent genetic alterations in the coding regions, with elevated frame shift-to-inframe ratios and lower transcript levels than wild-type alleles [474]. Genetic alterations in MSI tumors preferentially occur in euchromatic and intronic regions, compared to the preferential occurrence at the level of heterochromatic and intergenic regions in MSS tumors [474].

The substantial difference between these two colo-cancerogenetic pathways is supported by the observation that many genes recurrently mutated in MSI tumors—such as *RNF43*, *ATM*, *BRAF*, *R-Spondin* family gene fusions—are only rarely observed in MSS tumors. The model of progressive acquisition of mutations during colon cancerogenesis was challenged by an alternative model, the “Big Bang” model, which proposes that tumors grow predominantly in a single expansion, producing—early in the tumor history—numerous intermixed subclones that are not subject to stringent selection (neutral evolution). According to these two models, it can now be proposed that colorectal cancers evolve through processes of clonal selection and neutral evolution. These two different evolutionary mechanisms underline different molecular events characterizing the natural history of the tumor, namely gradual genotype changes—dependent upon the steady accumulation of single-nucleotide variants—and punctuated genotype changes—dependent upon large-scale copy number alterations [475].

Next generation sequencing of tumor specimens from large colorectal cancer patient cohorts has led to major advances in elucidating the genomic landscape of these tumors. This approach may have important implications for therapy optimization. However, at the clinical level, this approach is limited by the need for tumor tissue samples suitable for sequencing in adequate quantity and with a sufficient percentage of tumor cells, which are not available for all colorectal cancer patients. The recent development of cell-free DNA testing, based on the presence of the tumor circulating DNA present in colorectal cancer patients, represents an attractive resource for genomic studies and allows us to bypass these limitations [476]. Recent studies based on large sets of patients strongly support the use of cell-free DNA for the determination of a genomic landscape of colorectal cancer patients [476].

Parallel to the studies characterizing the genetic defects underlying colorectal cancer development, consistent efforts have been made to characterize the gene expression pattern as a tool to better understand tumor-related deregulation of gene expression and intertumor heterogeneity. A great effort to share large-scale data, based on six independent transcriptomic systems, has led to the proposal of a consensus molecular classification of colorectal cancer, enabling the categorization of most tumors into one of four different subtypes [251]. This system is further implemented by the inclusion of information derived from genomic analysis in these four subgroups. Tumors characterized by MSI belong to the CMS1 subtype, while CIN tumors have a more heterogeneous gene expression pattern and span from CMS2 to CMS4. The development of this consensus classification represents an important progress in the study of colorectal cancer and allows a harmonization at the level of various studies in the classification of these tumors. The molecular classification of colorectal cancers helped to define prognostic factors and to predict patient survival. A large screening analysis showed that MSS, CIMP^+^, BRAF^+^, as well as MSS, CIMP^−^, and KRAS^−^ had the highest disease-specific mortality; subjects with MSS, non-CIMP, BRAF^−^ and KRAS^+^ also have high disease-related mortality [138]. A meta-analysis of several pooled studies showed that among MSS^+^ tumors, those with mutations of KRAS or BRAF are associated with decreased survival rates compared to tumors with no KRAS or BRAF mutations [477].

The study of colorectal cancers has also shown that the tumoral stromal content may considerably impact the transcriptional classification of colorectal cancer with clinical and biological implications. Lineage-dependent stromal transcriptional components could play a more relevant role than stable expression features related to cancer cells. Thus, developing a system that minimizes the disturbing effect of the stromal tumoral component, a classification was proposed that identifies five colorectal cancer intrinsic subtypes (CRIS), characterized by peculiar molecular, functional and phenotypic properties: (1) CRIS-A: mucinous, glycolytic, enriched for microsatellite instability or KRAS mutations; (2) CRIS-B: high TGF-β signaling, epithelial-to-mesenchymal transition, associated with poor prognosis; (3) CRIS-C: high EGFR signaling and, consequently, sensitivity to EGFR inhibitors, TP53 mutations, high CIN; (4) CRIS-D: high WNT signaling, IGF2 overexpression and amplification, high CIN; (5) CRIS-E: Paneth cell-like phenotype, TP53 and KRAS mutations, high CIN [478]. CRIS-B identifies a colorectal cancer subtype associated with poor prognosis [478]. Interestingly, the correspondence between CSM and CRIS subtypes showed that CSM1 mainly corresponds to CRIS-A and, in part, also to CRIS-B; CSM2 mainly corresponds to CRIS-C, but also to CRIS-D and CRIS-E; CSM3 mainly corresponds to CRIS-A; CSM4 corresponds to the five CRIS subtypes [478]. Stromal-derived intratumoral heterogeneity undermines the molecular stratification of colorectal cancer patients into prognostic/predictive subgroups. This issue was systematically analyzed, exploring the impact of the analysis of various regions of the tumor (central tumor, invasive from of the tumor, lymph node metastasis) on the gene expression analysis using the CSM and CRIS classification systems. The classification following the CMS system was tumor-region-dependent, while the CRIS classification was patient-specific and not affected by the region of the tumor analyzed [262,479]. To obviate the limitations of the CMS classification system for the classification of cancer cell lines, organoids and xenografts were derived from colorectal cancers and a CMS classifier (CMS scaller) optimized for preclinical models was developed [265]. The limitations of the CMS classifier in identifying some colorectal cancer cases cannot be bypassed using laser-capture microdissected tumoral tissue [480]. These studies have confirmed that CRIS is a more robust patient stratifier than CMS, and also indicate that epithelial enrichment by microlaser dissection cannot eliminate the potential for stromal-derived intratumor heterogeneity to undermine patient stratification by the CMS classifier. The existence of a tumor heterogeneity in CMS subgroups is also supported by other studies. Thus, the study of multiple regions of CMS4 colorectal cancers and the analysis of PDGFRA, PDGFRB, PDGFC and KIT showed the existence of a consistent intratumor heterogeneity [481].

Although there is some uncertainty about the molecular mechanisms of colorectal tumor progression from early tumor lesions to advanced tumors, there is no doubt that the majority of colorectal cancers are preceded by adenomas. It was estimated that adenomas of the colon are present in 20% to 53% of the USA population older than 50 years of age [482]. These considerations supported programs of colon adenoma detection and removal by colonoscopy. On the other hand, epidemiological studies have shown that colorectal cancer incidence increases from 50 years of age. Several screening procedures, including colonoscopy, flexible sigmoidoscopy, computed tomography, or fecal-based screening are currently used. Colonoscopy is the most complete modality for the detection and removal of colon polyps [483]. However, some issues related to its invasiveness, inconvenience due to diet restriction and bowel preparation and possible adverse events resulting in sedation and a colonoscopic procedure (bleeding, perforation) limit the choice of general colonoscopy as the routine screening strategy. Flexible sigmoidoscopy is the preferred method for endoscopists because for this procedure sedation is not necessary and less bowel cleansing is required, as compared to colonoscopy. Several studies have addressed the impact of flexible sigmoidoscopy or of colonoscopy in reducing colorectal cancer mortality. Studies have been based on flexible sigmoidoscopy or colonoscopy for reducing colorectal cancer mortality. Studies based on flexible sigmoidoscopy have provided evidence of a significant reduction (by 50%) of mortality from distal colon cancer, while mortality from proximal colon cancer was unaffected [484,485]. Importantly, the positive impact of flexible sigmoidoscopy on colorectal cancer mortality was confirmed in a large screening carried out in Norway in the context of a randomized clinical trial. After about 11 years of follow-up, the colorectal cancer mortality in the screening group was 31.4 per 100,000 person-years, vs. 43.1 in the control group [486]. Importantly, a recent pool analysis of randomized trials based on flexible sigmoidoscopy screening showed that this procedure was effective in reducing colorectal cancer mortality in both younger and older men, and in women younger than 60 [487]. Interestingly, even in a follow-up after 17 years, a single flexible sigmoidoscopy was confirmed to protect from colorectal cancer diagnosis and death [488]. Colorectal cancer mortality was lower among patients who had low-risk, intermediate-risk adenomas removed by colonoscopy polypectomy [489,490,491]. These studies are important because they indicate that the early monitoring of the colorectal cancer strategy may represent an effective preventive approach and strongly support the disease development from precursor lesions.

The American Society for Clinical Pathology together with the College of American Pathologists, the Association for Molecular Pathology, and the American Society of Clinical Oncology have recently published a guideline for molecular biomarkers in the evaluation of colorectal cancer. The main recommendations contained in this study are the following: testing for *RAS* mutations as a negative predictor of response to EGFR-targeted monoclonal antibodies; *BRAF* mutations and MMR status for their prognostic value and as heralds of Lynch syndrome; there is no sufficient evidence to recommend the systematic mutational analysis of *PI3KCA* (although retrospective studies have suggested improved survival with post-operative aspirin use in patients whose colorectal cancers harbor a *PI3KCA* mutation) and the analysis of *PTEN* alterations [492].

The development of a molecular classification and the understanding of the main pathways of colorectal cancerogenesis were of fundamental importance for attempting new treatment strategies for subsets of patients. Recent clinical studies have suggested a new treatment strategy for colorectal cancers associated with MSI-H. Metastatic MSI-H, MMR-deficient colorectal cancer has a poor prognosis after treatment with conventional chemotherapy and exhibits high levels of tumor neoantigens, tumor-infiltrating lymphocytes, and checkpoint regulators. These tumor features are associated in other tumor types with the response to the PD-1 blockade. On the basis of encouraging preliminary results, in May 2017 the Food and Drug Administration (FDA) approved Pembrolizumab, a PD1 inhibitor, for the treatment of adult and pediatric patients with unresectable or metastatic MSI-H solid tumors (including colorectal cancers), regardless of tumor site or histology. Recently, the results of ongoing clinical trials based on PD1 targeting in MSI-H colorectal cancer patients have been reported. In a first study, in metastatic MSI-H colorectal cancers, Overman and coworkers reported that in a phase II study, with a median follow-up of 12 months, an objective response was found in 31% of treated patients and 69% of patients had disease control for 12 months or longer [125]. In a second study, Overman et al. reported a durable clinical benefit with Nivolumab (anti-PD1) plus Ipilimumab (anti-CTLA4) in MSI-H metastatic colorectal cancer patients, with an overall survival at 12 months of 85% [493].

EGFR inhibitors prevent the growth of colorectal cancer cells and have shown benefit in the treatment of metastatic colorectal cancer patients, whether used as single agents or in combination with chemotherapy. A significant clinical benefit was reported in clinical trials involving the use of EGFR Mab, but not EGFR tyrosine kinase inhibitors. However, there is still debate concerning the patient populations gaining the maximum benefit from EGFR inhibition. The analysis of a large set of data derived from randomized clinical trials allowed several important conclusions to be reached: (i) the addition of EGFR Mab to either chemotherapy or best supportive care improved PFS, OS and tumor response in *KRAS*-WT metastatic colorectal cancer patients; (ii) the use of EGF TKIs was without clinical benefit in KRAS-mutant metastatic colorectal cancer patients; and (iii) EGFR Mab, combined with Bevacizumab, is of no clinical value [494].

The molecular characterization of colorectal cancers provided evidence that about 2% of these patients at all stages display *HER2* overexpression and 5% among *KRAS*-WT metastatic patients [495]. *HER2* overexpression represents a potential therapeutic target in these patients. In the HERACLES-A trial, 33 *KRAS* exon 2 WT, *HER-2* positive metastatic colorectal cancer patients were treated with a combination of lapatinib and trastuzumab, achieving an overall response rate of 30%, with two complete responders [135]. Similar results were reported in the MyPathway trial, which reported a 38% objective response with trastuzumab and pertuzumab in 34 *HER2*-mutant *HER2*-positive colorectal cancer patients [496]. Importantly, patients with concomitant *HER2* amplification and *KRAS* mutations failed to respond to anti-HER2 treatment [497].

The studies carried out in the last years have shown the existence, in addition to intertumor heterogeneity, of a considerable degree of intratumor heterogeneity. The intratumor heterogeneity is related to genetic heterogeneity, epigenetic heterogeneity and functional heterogeneity. The intratumor heterogeneity is a process related to tumor evolution and progression, implying the growth of coexisting clones that vary over time, depending on the mutation rates and on the selective pressures; in consequence of their genetic and epigenetic heterogeneity, the clones have different functional properties, in terms of proliferation, ability to form metastases or to respond to specific therapies. The presence of intratumor heterogeneity poses a great challenge to enabling precision therapy, since not only the absence or presence of a given genetic abnormality, but also its prevalence in tumors are major determinants of the therapeutic response to a specific treatment.

In parallel to the studies on the characterization on molecular abnormalities of colorectal cancers, tremendous progress has been made in the identification and characterization of normal intestinal stem cells and their malignant counterparts. The intestinal epithelium is one of the most frequently renewing tissues in the human body, with a turnover period of 3–5 days in the small intestine. The rapid renewal of the intestinal epithelium is ensured by the stem cells situated at the bottom of crypts. The gene encoding LGR5, identified as the molecular marker of intestinal stem cells, is one of the WNT target genes in intestinal cells and labels the cells at the crypt base of both the small intestine and colon in adults, which were found to be intestinal stem cells by genetic lineage tracing. Several genes have been subsequently identified as markers of intestinal stem cells using genetic lineage tracing or in vitro clonogenetic assays of sorted cells, thus allowing the identification of quiescent Bmi1^+^ intestinal stem cells. These studies have generated a model implying the existence of multiple intestinal stem cells, some ensuring the tissue homeostatic renewal of the intestinal epithelium and the other acting as reserve stem cells ensuring tissue repair following various types of tissue injury. Therefore, there is a considerable hierarchy and plasticity with the intestinal stem cell compartment and these properties governing tissue homeostasis and regeneration and contributing to oncogenic transformation leading to colorectal cancers.

In parallel, dramatic progress has been made in the growth in vitro of intestinal cells. Particularly, organotypic intestinal epithelial cultures have been developed. The development of organoid intestinal cultures did take advantage of the initial observation that when the crypt cells of the mouse intestine are embedded in Matrigel, they are capable of long-term growth in the presence of EGF, Noggin and R-Spondin, forming 3D structures that protrude outward, resembling intestinal crypts. Following this initial study, this type of organotypic epithelial culture was developed for normal and malignant intestinal colonic cells. WNT ligands appeared essential for the growth of human colonic organoids. Organoid technology has enabled the reconstruction and expansion of intestinal tissue and has become a very powerful tool for investigating intestinal stem cell functions in vitro and in vivo in normal and malignant colonic tissue. Particularly, the organoid technology provides the possibility of culturing patient-derived colon tissue and colorectal cancers, while maintaining all functional and phenotypic characteristics.

The organoid technology is continuously improving, and very recently it was possible to obtain the labeling of stem cells in normal, benign and malignant tumor organoids of the human colon, thus offering a unique tool for the study of the contribution of intestinal stem cells to normal and tumoral development [498,499]. Importantly, one of these studies reported an orthoptic xenograft system for human colon organoids, enabling stable reconstruction of the human colon epithelium in vivo and using lineage tracing coupled with CRISP-Cas9, it was possible to demonstrate the self-renewal and multipotency of LGR5^+^ stem cells [499]. Importantly, this study also showed that in contrast to the rapid cycling properties of mouse LGR5^+^ stem cells, human LGR5^+^ stem cells are slow-cycling in vivo [499]. These observations strongly support the view that the organoid technology will rapidly improve our understanding of the dynamics of human intestinal stem cells in normal and malignant colonic tissue.

Most patients with colorectal cancer die as a result of metastatic disease; no prevalent genetic events have been clearly associated with metastatic colorectal cancers. However, several peculiar features of the tumor microenvironment—such as lack/low T cell infiltration, low type 1 T-helper cell response, reduced immune cytotoxic mechanisms or increased TGF-β levels—predict adverse outcomes in colorectal cancer patients. Recent studies strongly support a main role of TGF-β as a main mechanism driving the immune evasion of colorectal cancer cells [500] and attenuating tumor responses to PD-L1 blockade by contributing to the exclusion of T cells [501].

The existence of intestinal stem cells and the evidence accumulated showing that these cells could represent the cellular targets of oncogenic processes in the colon, represent a strong background against which to try to understand the mechanisms underlying colorectal cancer development during human life. Cancers are caused by mutations that may be inherited, induced by environmental factors, or result from DNA replication errors. Cancer risk in tissues within the alimentary tract strongly differ for the different organs, with a lifetime risk of being diagnosed for cancer ranging from 0.2% for the small intestine to 4.8% for the large intestine. In addition to tissue-specific factors, another component is related to inherited genetic variations, accounting for 5–10% if cancers. Finally, a third important factor is the stochastic effect associated with the lifetime number of stem cell divisions within each tissue, a condition exposing these cells to accumulate dangerous mutations over time. In support of this hypothesis, the analysis of 31 different tissues showed the existence of a good correlation between number of stem cell divisions and the lifetime risk of developing cancer [502]. Consequently, tissues with high adult stem cell turnover show higher cancer incidence when compared to tissues with low adult stem cell proliferation rates. This conclusion was reinforced by other analyses exploring the relationship between cancer incidence and the number of normal stem divisions from 17 different cancers in different countries, regardless of their different environments. The results of this study supported a main role for DNA replication errors, responsible for two-thirds of the mutations occurring in human cancers [502]. This observation strongly supports the importance of early detection and intervention to reduce deaths from many cancers, such as colorectal cancer, arising from unavoidable mutations deriving from DNA replication errors [503]. The relevance of these mechanisms in the genesis of human colorectal cancer was directly evaluated through the analysis of the accumulation of tissue-specific mutations in adult colon stem cells during life. Mutations accumulate steadily over time in both adult small intestine and large intestine stem cells, at a rate of approximately 40 new novel mutations per year, despite the large difference of tumor incidence between these two tissues [504]. Mutational signatures can be attributed to the spontaneous deamination of methylated cytosine residues, reflecting their high rate of intestinal adult stem cell division [504]. Importantly, the mutation spectra of driver genes in colon adult stem cells reflect the mutation spectra in colorectal cancers, thus suggesting that the intrinsic mutational process in adult intestinal stem cells can initiate colon cancerogenesis [504]. These observations are particularly important because they indicate that adult intestinal stem cells tend progressively to accumulate mutations with age with a similar rate at the level of the two near tissues—the small and large intestine—but the latter is more prone than the former to develop cancers, thus indicating the existence of a tissue-specific component restricting or favoring the tumoral process.

The human colon contains trillions of bacteria, separated from the colonic epithelium by a mucus layer. The mucus is of fundamental importance because it limits bacterial–epithelial cell contact, promoting tolerance to foreign antigens, limiting inflammatory mucosal responses, and impeding the invasion of the colon epithelia by bacteria. However, the generation of bacterial breaches within the colonic mucosa results in biofilm formation and the development of an inflammatory response. It was suggested that commensal and pathological bacteria may promote colorectal cancer development though defects in the tumor surface barrier occurring during the early stages of cancer formation, thus invading colonic tissue and eliciting an inflammatory response, and by generating compounds genotoxic to colon epithelial cells and, through this mechanism, accelerating the malignant progression [505,506]. Some bacterial species seem particularly prone to favoring colorectal cancer development. In this context, several studies have suggested a positive association between *Fusobacterium* and, particularly, *Fusobacterium nucleatum* and colorectal cancer development [507]. Even stronger associations were observed in colorectal cancers proximal to the splenic flexure and CIMP-high colorectal cancers [507]. Additional data also suggested shorter overall survival in patients with increased *Fusobacterium nucleatum* DNA in the tumoral tissue [507]. The mechanisms responsible for the enrichment of *Fusobacterium nucleatum* in colon cancer tissues are unclear but could be related to changes in the colon microenvironment [507]. However, a recent study reassessed the problem of *Fusobacterium nucleatum* infection prevalence in human colorectal carcinoma and showed that the difference in *Fusobacterium nucleatum* expression between the colorectal cancer tissue and adjacent normal tissues was smaller than previously reported, thus raising some doubts about this issue [508].

Interestingly, a recent study provided evidence that patients with familial adenomatous polyposis harbor colonic biofilms containing tumorigenic bacteria [509]. In fact, Dejea and coworkers identified patchy bacterial biofilms predominantly composed of *Escherichia coli* and *Bacteroides fragilis* in polyps developing in the colon of FAP patients [509]. Genes encoding for cobactin and *Bacteroides fragilis* toxins were highly enriched in FAP patients’ colonic mucosa compared to healthy controls [509]. These observations have suggested a link between early neoplasia and tumorigenic bacteria [509].

## Figures and Tables

**Table 1:** Hereditary syndromes associated with frequent colorectal cancer (CRC).

Syndrome	Inheritance	Genes	Functions	Phenotype	Risk of CRC	Frequency in CRC
Lynch Syndrome	Dominant	*MLH-1*, *MSH-2*, *MSH-6*, *PMS-2*, *EPCAM*	DNA mismatch repair. The mutations determine defects in DNA repair and high microsatellite DNA instability	Early-onset colorectal cancer, high penetrance, increased risk of extra-intestinal cancers. No increase in the frequency of adenomatous polyps. MMR-deficient crypt foci are observed	50–80%	2–4%
Familial Adenomatous Polyposis	Dominant	*APC* (truncating nonsense or frameshift mutations)	WNT signaling	Phenotype strong: >1000 polyps (mutations 1250–12,654); phenotype mild (mutations in 5′ and 3′). APC loss is an initiating event in adenoma development. Polyp formation is preceded by aberrant foci formation	100%	<1%
MUTYH-Associated Polyposis	Recessive	*MUTYH* (biallelic germline mutations)	DNA base excision repair protein for repair of mismatches	Mild phenotype with <100 polyps	43–100%	<1%
Proofreading Polymerase-Associated Polyposis	Dominant	*POLD1* (polymerase δ1), *POLE* (polymerase ε). In mutants, proofreading activity is lost, while polymerase activity is maintained.	Proofreading and repair of polymerase errors during DNA replication	<100 intetsinal polyps; early-onset CRC and extracolonic tumors. CRCs, POLE-mutated are mutually exclusive with MMR deficiency and have increased CD8^+^ lymphocyte infiltration.	High	1%
Familial Juvenile Polyposis	Dominant	*SMAD4*, *BMPR1*	TGF-β signaling	Multiple juvenile polyps found in the colon and stomach. In patients with SMAd4 mutations, association with hereditary hemorrhagic teleangectasia.	39–68%	
Peutz-Jeghers Syndrome	Dominant	*STK11*	Multiple signaling pathways	Mucocutaneous melanin hyper-pigmentation, multiple intestinal hamartomatous polyps (masses of connective tissue, covered by intestinal epithelium).	39%	
Hereditary Mixed Polyposis	Dominant	*GREM1*	Bone morphogenetic protein antagonist; Transforming growth factor-β signaling	Multiple polyps found in the colon. Adenomas are the most frequent, both tubular and villous.	20%	
Cowden Syndrome or PTEN hamartoma tumor syndrome (PHTS)	Dominant	*PTEN* (Decreased PTEN protein expression)	Negative regulation of AKT signaling. PTEN loss causes chromosome instability.	Few colon polyps, frequent hamartomatous polyps, presence of intramucosal lipomas.	9–16%	
CHEK2	Dominant	*CHEK2* (CHEK2 1100 delC variant, observed in 4% on non-FAP-related hereditary polyposis)	Negative regulation of cyclin-dependent kinases	CHEK2 is observed in 4–5% of cases of hereditary nonpolyposis colorectal cancer. CHEK2 is a multiorgan cancer susceptibility gene.	Moderate	
Serrated Polyposis Syndrome	Dominant	*RNF43* (25%)	Negative regulation of the WNT/β-catenin signaling pathway	Few (<5) to >20 serrated polyps occurring proximal to the sigmoid colon.	<50%	<1%
NTHL1-Associated Adenomatous Polyposis	Recessive	*NTHL1* (DNA glycosylase gene)	Base excision repair	1–>50 polyps; development of CRCs mismatch repair-proficient	Unknown	

Full names of genes can be found at Table of Abbreviations.

**Table 2:** The gene expression-based consensus molecular subtypes (CMS) of colorectal cancer.

Tumor Subtype	Proportion	Main Genomic Features	Genetic Drivers	Tumor Location (Proximal (P) or Distal (D))	Precursor Lesions	Gene Expression Signature	Therapeutic Targeting	Prognosis
CMS1 Hypermutated	14%	Hypermutated 95% MSI^+^ 70% CIMP^+^ 65% CANs^+^ 20% Frequent Mutations: BRAF 40% APC 35% TP53 30% KRAS 25%	*BRAF*	74% P 26% D	Serrated	Strong immune activation; High PD1 activation; NK cell and TH1 infiltration; Low stromal infiltration	Immune Response (sensitivity to immune check inhibitors); HSP90	Intermediate (worse prognosis after relapse); these tumors tended to be diagnosed at less advanced stages (I–II)
CMS2 Canonical	40%	Hypermutated 2% MSI^+^ 2% CIMP^+^ 4% CNAs^+^ 96% Frequent Mutations: BRAF 0% APC 80% TP53 70% KRAS 30%	*APC*	20% P 80% D	Tubular	High expression of WNT and MYC targets; epithelial differentiation; upregulation of Src and cell cycle pathways; upregulation of the miR 17–92 cluster (MYC target); very low immune infiltration and activation;	EGFR (sensitivity to anti-EGFR MAbs); HER2	Good (superior survival rates also after relapse, with some long-term survivors)
CMS3 Metabolic	10%	Hypermutated 30% MSI^+^ 15% CIMP^+^ 21% CNAs^+^ 54% Frequent Mutations: BRAF 10% APC 75% TP53 30% KRAS 70%	*KRAS*	55% P 45% D	Unknown	upregulation of multiple metabolic signatures (sugar, amino acids, fatty acids, nitrogen); epithelial differentiation; low immune infiltration and activation; very low stromal infiltration;		Intermediate
CMS4 Mesenchymal	25%	Hypermutated 2% MSI^+^ 3% CIMP^+^ 9% CNAs^+^ 86% Frequent Mutations: BRAF 5% APC 65% TP53 55% KRAS 40%	*miR-200* (downregulation) *TGF-β* pathway	34% P 66% D	Serrated	upregulation of genes implicated in epithelial-to-mesenchymal transition and of signatures associated with the activation of TGF-β signaling, angiogenesis matrix remodeling pathways and the complement-mediated inflammatory system. High stromal infiltration. High VEGF/VEGFR and integrin β3 pathways.	PDGFRA; KIT; HSP90	Poor (worse overall survival and relapse- free survival); these tumors tended to be diagnosed at more advanced stages (III–IV)

Full names of genes can be found at Table of Abbreviations.

## Abbreviations

ACVR2A	Activin A Receptor type 2A
APC	Adenomatous Polyposis Coli
ARID1A	AT-Rich Interaction Domain 1A
ATM	Ataxia Telangiaectasia Mutated
ATOH1	Atonal BHLH Transcritpion Factor 1
bFGF	Basic fibroblast growth factor
BAX	Bcl-2-Associated X protein
BMP	Bone Morphogenetic Protein
BMPR1	Bone Morphogenetic Protein Receptor 1
BRAF	B-RAF
CASP8	Caspase 8
CDKN2A	Cyclin-Dependent Kinase
CDX2	Caudal Homeobox 2
CIN	Chromosomal Instability
CIMP	CpG Island Methylator Phenotype
CHEK2	Checkpoint kinase 2
CMS	Consensus Molecular Subtype
CNA	Copy Number Alteration
CRC	Colorectal Carcinoma
CtBP1	C-terminal Binding Protein 1
CTNNB1	Catenin beta 1
dARE	Depleted in AU-Rich Elements
EGFR	Epidermal Growth Factor Receptor
EIF3E	Eukaryotic Translation Initiation Factor Subunit 3E
EPCAM	Epithelial Cell Adhesion Molecule
ERB	Erythroblastic Leukemia Viral Oncogene Homolog
ERK	Extracellular Signal-Regulated Kinase
FBXW7	F box and WD Repeat Domain Containing 7
FAP	Familial Adenomatous Polyposis
GREM1	Gremlin 1
HLA	Human Leukocyte Antigen
HSP90	Heat Shock Protein 90
HLH	Helix-Loop-Helix
KRAS	Kristen Rat Sarcoma
LGR5	Leucine-Rich Repeat-Containing G-Protein Coupled Receptor 5
MAPK	Mitogen-Activated Protein Kinase
MEK	MAPK Extracellular Signal-Regulated Kinase
MGMT	Methyl Guanine DNA Methyltransferase
MLH1	MUTL Homolog 1
MMR	Mis-Match Repair
MSH-1	MutS Homolog 1
MSH-2	MutS Homolog 2
MSH-6	MutS Homolog 6
MSI	Microsatellite Instability
MSS	Microsatellite Stable
MUTYH	MUTY Homolog
NFkB	Nuclear Factor Kappa-Light
NRAS	Neuroblastoma Rat Sarcoma
NK	Natural Killer
NTHL1	NTH Like DNA Glycosylase 1
P16	Protein 16
PDGRFA	Platelet Derived Growth Factor Receptor Alpha
PGE2	Prostglandin E2
PI3K	Phosphatidyl-Inositol-3 Kinase
PI3KCA	Phosphatidyl-Inositol-3 Kinase Catalytic
PMS2	PostMeiotic Segregation increased 2
POLD1	Polymerase δ1
POLE	DNA Polymerase E subunit
PTEN	Phosphatase and Tensin Homolog
PTGS2	Prostglandin-Endoperoxide Synthase 2
PTPRK	Protein Tyrosine Phosphatase Receptor Type K
RFN43	Ring Finger Protein 43
RSPO2	R-Spondin 2
RSPO3	R-Spondin 3
SCID	Severe Immunodeficiency
SHH	Sonic HedgeHog
SMAD4	Small Mother Against Decapentaplegic 4
SMO	Smoothened, Frizzled Class Receptor
SOX2	SRY-Box 2
SOX9	SRY-Box 9
STK11	Serine/Threonine Kinase 11
TCGA	The Cancer Genoma Atlas
TCF7L2	Transcription Factor 7 Like 2
TGF-β	Transforming Growth Factor-Beta
TGFBR2	Transforming Growth Factor Receptor 2
TIAM1	T Cell Lymphoma Invasion and Metastasis
VEGF	Vascular Endothelial Growth Factor
VEGFR	Vascular Endothelial Growth Factor Receptor
WNT	Wingless-Type MMTV Integration Site Signaling
YAP1	Yes-Associate Protein 1
